# Enhancing reproducibility of gene expression analysis with known protein functional relationships: The concept of well-associated protein

**DOI:** 10.1371/journal.pcbi.1007684

**Published:** 2020-02-14

**Authors:** Joël R. Pradines, Victor Farutin, Nicholas A. Cilfone, Abouzar Ghavami, Elma Kurtagic, Jamey Guess, Anthony M. Manning, Ishan Capila

**Affiliations:** Momenta Pharmaceuticals, 301 Binney Street, Cambridge, Massachusetts, United States of America; University of Ottawa, CANADA

## Abstract

Identification of differentially expressed genes (DEGs) is well recognized to be variable across independent replications of genome-wide transcriptional studies. These are often employed to characterize disease state early in the process of discovery and prioritize novel targets aimed at addressing unmet medical need. Increasing reproducibility of biological findings from these studies could potentially positively impact the success rate of new clinical interventions. This work demonstrates that statistically sound combination of gene expression data with prior knowledge about biology in the form of large protein interaction networks can yield quantitatively more reproducible observations from studies characterizing human disease. The novel concept of Well-Associated Proteins (WAPs) introduced herein—gene products significantly associated on protein interaction networks with the differences in transcript levels between control and disease—does not require choosing a differential expression threshold and can be computed efficiently enough to enable false discovery rate estimation via permutation. Reproducibility of WAPs is shown to be on average superior to that of DEGs under easily-quantifiable conditions suggesting that they can yield a significantly more robust description of disease. Enhanced reproducibility of WAPs versus DEGs is first demonstrated with four independent data sets focused on systemic sclerosis. This finding is then validated over thousands of pairs of data sets obtained by random partitions of large studies in several other diseases. Conditions that individual data sets must satisfy to yield robust WAP scores are examined. Reproducible identification of WAPs can potentially benefit drug target selection and precision medicine studies.

## Author summary

Gene expression studies are commonly used to characterize biological systems. Genes identified in such experiments as expressed at different levels between conditions (e.g. healthy vs. disease) can indicate biological functions that are important in this context. However, it is well-recognized that such findings can vary substantially across independent investigations. We quantified reproducibility here under a conservative control scenario that partitions a given data set in two, independently of experimental conditions, for multiple data sets characterizing several diseases in humans. Furthermore, we have shown that it is possible to obtain more reproducible findings than DEGs, which we term Well-Associated Proteins, characterizing differences in gene expression between healthy and disease states. This was accomplished by combining gene expression and prior knowledge of functional relationships between gene products accumulated over many studies and publications. Resulting Well-Associated Proteins can be computed efficiently enough to enable permutation controls and demonstrate on average higher reproducibility than differentially expressed genes, both within and across data sets. This suggests that Well-Associated Proteins may better reflect differences in biology when comparing disease and healthy states than DEGs, thus representing an important step towards identification of key disease drivers.

## Introduction

Microarrays and RNA sequencing are experimental technologies convenient for generating lists of Differentially Expressed Genes (DEGs) that characterize differences between two conditions, e.g. a disease versus its absence. It is commonly recognized that DEG identification can be highly variable over independent studies [1–3]. Some of the advances aimed at improving reproducibility of the DEG discovery included removal of DEGs with small fold changes between conditions [3, 4], as well as accounting for correlation among DEGs [5, 6], and relying on ranked lists of DEGs [7] when comparing DEGs across independent experiments. It has also been argued that high variability in the identified DEGs is an inherent property of gene expression studies and that development of new metrics of reproducibility might be desirable in this context [8].

The modular vision of biology [9] inspired development of a vast array of systems-based methodologies [10] quantifying differential regulation of gene sets representing known biological functions such as Gene Ontology (GO) categories [11], curated collections of pathways (e.g. KEGG [12] or Reactome [13]) or molecular signatures observed in previous gene expression studies (e.g. MSigDB [14]). These methods can potentially improve sensitivity and interpretability of gene expression experiments and have been benchmarked in several contexts [15–18]. Networks of known interactions between gene products represent a complementary approach for organizing genes by their functional relationships and have been used for differential analysis of gene expression data by multiple methods that have been extensively reviewed [10, 19, 20] and benchmarked [21, 22] as well. Studies using pathway networks in combination with genome-wide patient profiling data include works by [23] that developed permutation tests to demonstrate significant connectedness on PPI network of genomic loci associated with several common diseases and [24] that found higher replicability of the predictions of patient response to treatment in an independent study, when patient-level gene expression data was combined with the network of causal relationships representing transcriptional regulation. These approaches utilize prior biological knowledge, are well established for the analysis of gene expression data and are routinely used within high throughput biology studies [25].

Assessing the gain in reproducibility of the systems-based analyses results due to the use of prior biological knowledge (e.g. functional categories or pathway networks) still remains difficult to address. A survey of the studies evaluating reproducibility of the findings at the level of genes and/or gene sets [26–31] illustrates a variety of challenges associated with this task. They include: the choice of reference gene expression data and gene set knowledgebase, the selection of analyses methodologies to compare, the definition of reproducibility metrics, and, most importantly, the burden of interpreting results when comparing reproducibility metrics for different types of entities (e.g. as calculated for genes vs. those for GO categories).

This paper introduces a novel concept of a Well-Associated Protein (WAP) that quantifies the association of gene (product) on a protein interaction network (STRING [32]) with the genes that are more significantly regulated in the experiment. This development enables comparison of the reproducibility of findings across independent experiments within the same universe of genes represented in the protein interaction network that can be scored both for their individual differential expression (as DEG) or, as a WAP, for their association on the network with the most significantly regulated genes. The significance of the WAP association accounts for the total number of interactions of each protein (protein degree) on the network, and computation of this significance is fast enough to enable permutation controls. This allows for identification of gene products which have a significantly large number of known associations to the genes that are most perturbed in the experiment, without actually choosing a threshold of differential expression (consequently incurring inevitable information loss). Therefore resulting WAPs can attain statistical significance while not being themselves differentially expressed. Thus they can extend standard gene expression analysis results while leveraging the systems-level of information encoded in the protein network and the entire compendium of data obtained in a gene expression experiment.

By considering only genes which are represented both in gene expression data and on the protein interaction network, this approach enables the direct comparison of the reproducibility metrics for DEG and WAP rankings. Comparisons presented in this paper demonstrate that, under easily-quantifiable conditions, higher average reproducibility of WAP identification versus that of DEG is observed over nine types of diseases.

This paper is organized as follows. After presenting computational details, reproducibility of WAP and DEG identification is examined over four large data sets where gene expression in skin samples is compared between Systemic Sclerosis (SSc) patients and non-SSc subjects, demonstrating greater reproducibility of top WAPs as compared to that of top DEGs across these data sets. Superior average robustness of top WAPs versus top DEGs in disease versus normal comparisons is then validated over thousands of data set pairs obtained by random partitions of eighteen large gene-expression studies incorporating eight other diseases (colon cancer, gastric cancer, endometriosis, hepatocellular carcinoma, non-small-cell lung carcinoma, lung adenocarcinoma, oral squamous cell carcinoma and psoriasis). Additionally, conditions for individual data sets contributing to the higher robustness of WAPs are examined. Finally, limitations of the approach and potential for further work are discussed.

In summary, the WAP score is a robust statistic which ranks gene products by the significance of their known interactions with the genes most perturbed in the experiment without having to choose a threshold of differential expression. Because WAP scores can be efficiently computed, false discovery rates can be numerically estimated by permutation techniques that preserve correlation of gene expression [33]. Identification of genes with significant WAP scores complements standard gene expression analysis, and extends it by identifying genes that are not themselves DEGs. Enhanced reproducibility of WAP identification versus traditional selection of DEGs is demonstrated under specific conditions, and holds at least across nine types of disease. Such an increase in reproducibility of the findings from gene expression studies, driven by the prior biological knowledge encoded in a protein network, suggests that the resulting WAPs may represent a robust description of the disease-related biology that is likely to be beneficial to drug target selection and precision medicine approaches.

## Materials and methods

### The concept of well-associated protein

**Fig 1:**
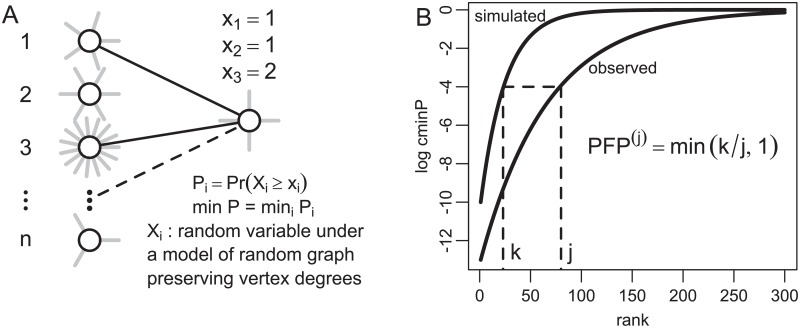
Illustration of WAP scores. A: Gene products are scored for their association *x*_*i*_ (number of interactions) to the top *i* DEGs, where *i* varies from 1 to the total number *n* of genes. Values of *x*_*i*_ are compared via edge-count probabilities *P*_*i*_, and a gene product is scored with its best corrected attachment p-value: c min_*i*_ *P*_*i*_. B: The profile of ranked c min *P* values is compared to profiles estimated after randomly assigning disease and control labels to samples (10^4^ permutations). This yields an estimated Proportion of False Positive (PFP) of WAPs at each rank *j*.

The approach presented herein identifies proteins, termed Well-Associated Proteins (WAPs), which have a large number of known functional relationships (i.e. interactions) in a protein interaction network with the genes that are significantly perturbed in gene expression data. The method utilized to identify WAPs is illustrated with Fig 1A. Consider the *n* genes which are represented both in a gene expression data set and the protein interaction network. Genes are ranked from the most differentially expressed (*i* = 1) to the least (*i* = *n*) using results of gene-level statistical models as warranted by a given study design (e.g. t-test, linear or mixed effects model, etc.).

The association of any gene product *j* to the top *i* DEGs is a function of *x*_*j*,*i*_, the number of known interactions in the protein network between *j* and this set. The value of *x*_*j*,*i*_ depends not only on *i* but also on the degrees (total numbers of interactions) of all considered gene products. A possible approach to account for degrees is to compare *x*_*j*,*i*_ to the expected number of interactions when randomly rewiring edges in the whole network, while exactly preserving the degree *k* of each gene product [34]. This however requires extensive numerical simulations, because explicit derivation of the expected number of interactions under this random graph model is a hard problem [35].

By considering a relaxed random graph model, which only preserves degrees on average over realizations [36], one can easily obtain the distribution of random variable *X*_*j*,*i*_ [37]. Briefly, any possible interaction between two proteins of degrees *k*_*u*_ and *k*_*v*_ is represented by a Bernoulli random variable of parameter *b*_*uv*_ = *k*_*u*_*k*_*v*_/2*M* (*b*_*uv*_ is the probability of this interaction to exist), where *M* is the total number of interactions in the network, and interactions are modeled as independent variables (Section 1 in S1 Text provides additional technical details). For sparse networks (*b*_*uv*_ ≪ 1), counting interactions is then equivalent to summing independent Bernoulli variables of small parameters and the resulting distribution can be approximated by a Poisson distribution [38]. The distribution of random variable *X*_*j*,*i*_ is therefore Poisson with parameter

$$\begin{array}{c} \lambda_{j,i}=\frac{k_{j}}{2M}\sum_{h\leq i,h\neq j}k_{h}. \end{array}$$ (1)

After some normalization [37], the probability of observing at least *x*_*j*,*i*_ interactions is given by

$$\begin{array}{c} \text{Pr}(X_{j,i}\geq x_{j,i})=\frac{e^{-\lambda_{j,i}}}{\alpha_{j,i}}\sum_{h=x_{j,i}}^{i}\frac{\lambda_{j,i}^{h}}{h!};\;\alpha_{j,i}=\sum_{h=0}^{i}\frac{\lambda_{j,i}^{h}}{h!}. \end{array}$$ (2)

Notice that, because λ_*j*,*i*_ increases with *i*, the p-value is conditional not only to the observed number *x*_*i*_ of interactions, but also to the considered number *i* of DEGs. This implies that there is no need for choosing a threshold (bound on *i*) which defines differential expression. The above p-value is referred to as *P*_*j*,*i*_ for short. This type of p-value enables comparison of diverse protein sets for their connectedness in a network, while taking into account protein degrees [37, 39–41]. One can compare values of *P*_*j*,1_, *P*_*j*,2_,…, *P*_*j*,*n*_ and the best association of gene product *j* to DEGs corresponds to min_*i*_ *P*_*j*,*i*_. Again, this quantity does not require choosing a threshold of differential expression. To compare best association scores across proteins one must further correct by their degree, because the operation of taking the minimum can yield bias towards proteins with large degrees and due to lower values of *P*_*j*,*i*_ attainable for them (Section 3 in S1 Text). The corrected best association score for gene product *j* of degree *k*_*j*_ in the protein interaction network used herein is given by

$$\begin{array}{c} \begin{array}{c} \text{c}\;\text{min}\;P_{j}=(\rho (k_{j})/\rho (1))\underset{i}{\text{min}}P_{j,i},\\ \text{with}\;\;\rho (k)=k^{-\alpha }e^{-\beta },\;\;\alpha =0.1799,\;\;\text{and}\;\;\beta =1.056. \end{array} \end{array}$$ (3)

When the total number *n* of proteins in the network is at least a few thousand, the correction depends only on *k*_*j*_. Gene products are next sorted by ascending values of corrected best association score c min *P*:

$$\begin{array}{c} \text{c}\;\text{min}\;P^{\left(1\right)}\leq \ldots \leq \text{c}\;\text{min}\;P^{\left(j\right)}\leq \ldots \leq \text{c}\;\text{min}\;P^{\left(n\right)}. \end{array}$$ (4)

Computation of all c min *P* values can be optimally performed in O(M), where *M* is the total number of interactions in the network (Section 2 in S1 Text).

Fast computation makes it possible to estimate a false discovery rate (FDR; the expected proportion of false positives at a given cutoff) for the observed values of c min *P*^(*j*)^ via permutation techniques in a reasonable time. Permutation, randomizing sample labels (healthy vs. disease) as described below, was employed to estimate proportion of false positives under the null hypothesis of interchangeability of observations in these two groups. The null hypothesis of randomizing sample labels is that of the primary interest for identifying WAPs representing differences between these two groups, unlike the null hypothesis of random rewiring of the pathway network, yielding actual values of minP, that was used to account for the wide ranging disparity of vertex degrees when scoring them for connectedness to more differentially regulated genes. For the sake of clarity, different acronyms will be used to emphasize the distinction between permutation-based estimates of FDR for WAPs and Benjamini-Hochberg [42] estimates of FDR for DEGs. Throughout this paper the former will be referred to as a Proportion of False Positives (PFP) and use of FDR will be reserved to represent the latter—Benjamini-Hochberg FDR for DEGs. To estimate PFP values for WAPs disease states are randomly shuffled between samples (thus preserving gene co-expression), genes are ranked for differential expression and values of c min *P*^(*j*)^ are estimated again. Observed values are compared to simulated ones: for the observed WAP score of rank *j*, the simulated profile yields *k* more significant scores, hence a first estimation of PFP^(*j*)^ = min(*k*/*j*, 1). The estimation is refined by averaging values of PFP^(*j*)^ over at least 10^3^ simulations. This process is illustrated with Fig 1B. WAPs with small PFP values are said to be significantly associated to the most differentially expressed genes.

**Fig 2:**
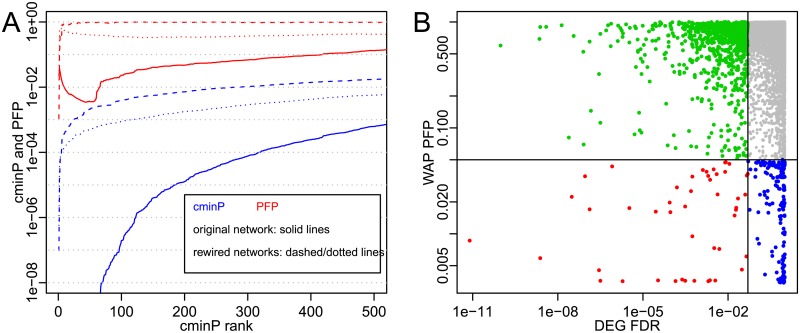
WAP and DEG scores for data set GSE58095. A: c min *P*(*j*) values (blue solid line) and PFP values (red solid line, 10^4^ permutations) as a function of their rank *j*. Dashed lines correspond to values obtained with a randomly rewired protein network and dotted lines to a randomly rewired network preserving the number of triangles. B: Scatterplot of WAP PFP and DEG FDR values. Thresholding DEG FDR and WAP PFP at 5% yields four categories of genes based on their significance: DEG and not WAP (green), DEG and WAP (red), WAP and not DEG (blue) and neither DEG nor WAP (gray).

Fig 2 provides an example of a c min *P* profile obtained with data set GSE58095 [43], where skin samples of Systemic Sclerosis (SSc) patients are compared to skin samples of healthy subjects. Differential expression is assessed by a two-sided t-test. The solid blue line in Fig 2A displays observed values of c min *P*^(*j*)^ as a function of rank *j*. For the sake of display, the vertical scale has been restricted to values between 10^−8^ and 1, and ranks *j* ≤ 70 yield smaller values down to *c* min *P*^(1)^ ≃ 10^−38^. The red solid line shows PFP values for each observed *c* min *P*^(*j*)^ estimated with 10^4^ permutations. There are 215 genes with PFP value less than 0.05, suggesting that DEGs in SSc versus healthy are specifically organized within the network of known protein functional relationships. To illustrate the importance of biological knowledge encoded in the protein network on WAP scores, results are also displayed for a randomly rewired network, as described in [34], which preserves vertex degrees (dashed lines) and for a similarly rewired network but containing the same number of triangles as the original network (dotted lines). The utilized method to create random triangles while preserving vertex degrees is described in [44]. Randomly rewired networks do not yield small PFP values, except for one gene: Ubiquitin C. This is because it connects to nearly half of the vertices on the network, so that the probability of the removal by random rewiring of all of the existing ubiquitin C interactions is negligibly small. All other proteins yield PFP values greater than 0.1 after rewiring, showing that true protein functional relationships are required to obtain significant WAP scores.

In Fig 2B, genes are partitioned into four categories by choosing a threshold of 0.05 on WAP PFP and/or DEG FDR, so as to yield an expected false-positive rate of only 5% for both DEGs and WAPs that are deemed significant. Gray dots correspond to genes which are neither significant DEGs (FDR > 0.05) nor significant WAPs (PFP > 0.05). Significant DEGs are represented by the union of green and red dots, for a total of ≈1100 genes. Among those, ≈50 are significant WAPs as well (red dots). These genes are not only differentially expressed, but also significantly connected to other DEGs via known interactions and this can be seen as additional evidence for their potential involvement in SSc. Lastly, significant WAPs which are not significant DEGs are represented by blue dots (≈160 genes). These genes can only be identified by combining gene expression and protein network knowledge: even though they are not significantly perturbed at the mRNA level, their significant connectedness on the network to the most perturbed genes in the SSc-normal comparison suggests their potential involvement in SSc.

### Quantification of gene scores reproducibility across data sets

In order to compare rankings of WAP and DEG scores for their robustness across data sets, it is required to define a measure of reproducibility. Reproducibility is quantified with the Jaccard index, which is a well-accepted measure of overlap [45]. Call $A_{i}$ and $B_{i}$ the top *i* genes, based on WAP or DEG scores, in two data sets A and B. The two data sets have been reduced to their common *n* genes, which are also represented in the protein network. The relative overlap of sets $A_{i}$ and $B_{i}$ is the Jaccard index:

$$\begin{array}{c} R_{i}=\frac{I_{i}}{U_{i}},\;\text{with}\;I_{i}=|A_{i}\cap B_{i}|,\;U_{i}=|A_{i}\cup B_{i}|, \end{array}$$ (5)

and where |S| stands for the number of genes in set S. Plotting *R*_*i*_ as a function of *U*_*i*_ provides a display of overlap between two data sets. The two curves obtained with WAP and DEG scores can then be visually compared. For *i* → *n* one trivially has *R*_*i*_ → 1. But local maxima of *R*_*i*_ for values of *U*_*i*_ less than *n* are indicative of remarkable overlap.

**Fig 3:**
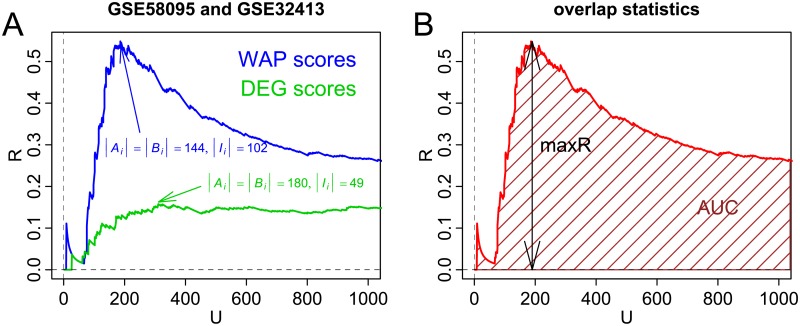
Illustrations of overlap profiles. A: Overlap profiles of WAP (blue) and DEG (green) scores across two gene expression data sets, where skin samples of SSc patients are compared to samples of non-SSc subjects. Values of $\left|A_{i}|=|B_{i}\right|$ and $\left|I_{i}\right|$ indicate sizes of gene sets and intersection between them corresponding to maxR values for WAP and DEG score profiles. B: The two summary statistics of an overlap profile.

To illustrate the quantification of reproducibility, two Systemic Sclerosis (SSc) data sets are considered: GSE58095 [43] and GSE32413 [46]. Gene expression was measured in skin samples of both SSc patients and non-SSc subjects. Differential expression is assessed by an absolute t-statistic between the two groups. Fig 3A displays overlap profiles across the two data sets, i.e. graphs of *R*_*i*_ as a function of *U*_*i*_, for WAP (blue) and DEG (green) scores. Relative overlap *R*_*i*_ tends to be higher with WAP scores than with DEG scores.

Overlap profiles provide visual representation of reproducibility across two data sets. To facilitate assessment over many pairs, two summary statistics are defined and illustrated with Fig 3B. The first statistic is called maxR and represents the height of the peak in the overlap profile:

$$\begin{array}{c} \text{maxR}=\underset{a\leq U_{i}\leq b}{\text{max}}R_{i}. \end{array}$$ (6)

Lower bound *a* = 50 is chosen to avoid large values due solely to the discrete nature of *R*_*i*_ at small *i* values. Upper bound *b* = 1, 000 is chosen, so that *R*_*i*_ does not trivially increase with *i* (*R*_*n*_ = 1 and *n* ≃ 14, 000). The second statistic is the area under the curve (overlap profile) between *i* = 1 and *i* = *b*:

$$\begin{array}{c} \text{AUC}=\frac{w_{1}R_{1}^{\prime }+w_{b}R_{b}^{\prime }}{2}+\sum_{i=2}^{b-1}w_{i}R_{i}^{\prime }\;\text{with}\;w_{i}=U_{i+1}^{\prime }-U_{i}^{\prime }. \end{array}$$ (7)

Notice that lower bound *a* = 50 on *i* is not utilized, because contribution of *i* ≤ *a* to the sum is small (*a*/*b* = 0.5%). The prime notation is used with *U* and *R* to reflect that these variables need to be modified when two or more successive values of *U*_*i*_ are equal: $R_{i}^{\prime }$ is then the average of the *R*_*i*_ values and $U_{i}^{\prime }$ is the smallest *U* value. As empirically demonstrated below using random partitioning of gene expression datasets, maxR and AUC statistics, although strongly correlated, are far from being entirely collinear, therefore representing complementary metrics for quantifying reproducibility of ranked lists of genes.

### Random partitions of a data set in two

The number of available pairs of independent gene expression studies which describe the same disease is limited. To enable larger sampling, an approach consists in partitioning a large data set in two. Namely, a data set having *n* disease samples and *m* control samples is randomly split in two data sets of sizes ⌊*n*/2⌋ + ⌊*m*/2⌋ and ⌈*n*/2⌉ + ⌈*m*/2⌉. Even with *n* = *m* = 10 there are more than 3 × 10^4^ possible partitions. This enables reasonable sampling and thus accurate estimation of overlap statistics distributions for DEG and WAP scores. In addition to being partitioned, data sets can also be perturbed for their disease/control composition. This enables sampling of data set pairs in which individual data sets have tunable characteristics (Section 4 in S1 Text).

### A multivariate statistic based on sample dissimilarities

A statistic to summarize the entire differential expression (i.e. over all genes) between two groups of samples is estimated as follows. Call *d*_*ij*_ the Pearson dissimilarity [47] between two samples *i* and *j*. Values of *d*_*ij*_ close to 0 mean that the two samples have similar expression values across all genes, and values close to 1 indicate dissimilarity. Such dissimilarities are commonly used, for instance in cluster analysis [48]. Call D and C disease and control groups of samples. A statistic called MVT, for Multi-Variate T [49, 50], is defined by

$$\begin{array}{c} \begin{array}{c} \text{mvt}\;(D,C)=\frac{d(D,C)}{s(D)+s(C)}\;\;\text{with}\;\;d(D,C)=\frac{\sum_{i\in D,j\in C}d_{ij}}{\left|D||C\right|}\\ \text{and}\;\;s(D)=\frac{2\sum_{i<j\in D}d_{ij}}{\left|D|(|D|-1\right)}. \end{array} \end{array}$$ (8)

Much like a t-statistic, mvt is the ratio of difference between groups (*d*) to their spread (*s*). Large values of mvt therefore indicate separation of the two groups. To define what large is, permutation testing is utilized. The observed value (mvt) is compared to values (MVT) obtained when randomly shuffling samples between groups D and C, and the numerically estimated p-value

$$\begin{array}{c} p=\text{Pr}(\text{MVT}\geq \text{mvt}) \end{array}$$ (9)

is small compared to 1 if the two groups are markedly different.

### Protein networks and gene expression data sets

The network of protein functional relationship utilized throughout this study is based on STRING V10 [32]. Interactions were restricted to those having a confidence score of at least 0.7 [51]. Results presented next for Systemic Sclerosis data sets are shown to be robust to changing this threshold (Section 4.2 in S1 Text). Additionally, PPI networks evaluated and/or derived in [52] and deposited to the Network Data Exchange (NDEx) repository [53–55] have been retrieved from NDEx using their identifiers provided by [52] (“Deposited Data” in “Key Resources Table” therein) using the Bioconductor package “ndexr” [56]. All utilized gene expression data sets were downloaded from the Gene Expression Omnibus database [57].

## Results

### Robustness of WAP scores across Systemic Sclerosis studies

Greater robustness of WAP scores as compared to that of traditional DEG scores is first illustrated with four gene expression Systemic Sclerosis (SSc) data sets (GSE58095 [43], GSE32413 [46], GSE9285 [58] and GSE45485 [59]). Skin samples of SSc patients are compared to skin samples of healthy subjects.

**Fig 4:**
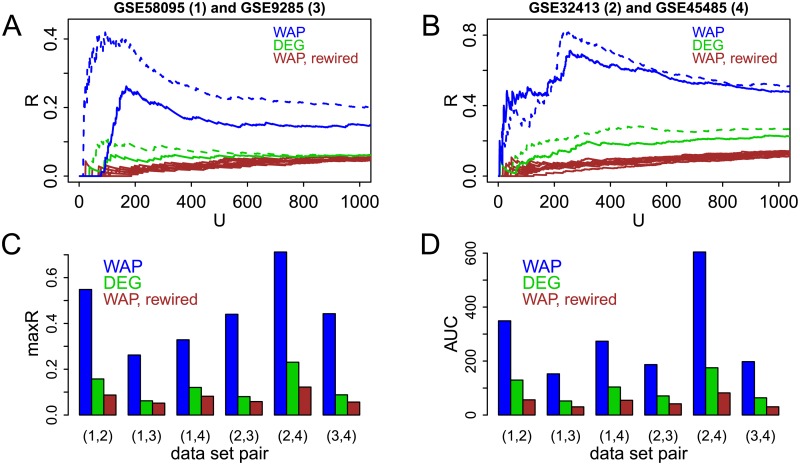
Reproducibility of WAPs and DEGs among four SSc data sets. Four gene expression data sets comparing skin samples between SSc patients and non-SSc subjects—GSE58095 (1), GS32413 (2), GSE9285 (3) and GSE45485 (4)—are used to obtain WAP and DEG scores. Blue and green colors represent overlaps between top scoring WAPs and DEGs respectively. A-B: Overlap profiles for two pairs of data sets. Solid profiles correspond to genes ordered by a two-sided t-test and dashed profiles—to ordering which further accounts for fold change (a gene rank is the average of its t-statistic and fold-change ranks). Brown profiles display results for ten randomly rewired protein networks. C-D: Overlap statistics maxR and AUC for all six data set pairs, when genes are ordered by a two-sided t-test. For randomly rewired networks, displayed values are averages.

To first assess reproducibility in a visual way, overlap profiles over the entire set of the top 1000 DEGs and WAPs are utilized. Panels A and B of Fig 4 display overlap profiles obtained with two pairs of data sets. Solid green lines show results obtained when ordering genes by their differential expression (SSc vs. healthy) based on a two-sided t-test. Solid blue lines display overlap profiles over the two data sets for WAP scores and green lines correspond to DEG scores. Ranks of WAP scores tend to be more reproducible than those of DEG scores, as attested by larger values of *R* over most of the overlap profile. WAP scores obtained with randomly rewired networks (brown) are less reproducible than DEG scores, illustrating that superior robustness of WAP scores over DEG scores relies on the existence of true biological interactions between gene products and is not a trivial artifact of different scoring between WAPs and DEGs.

Dashed lines in Fig 4A correspond to the results obtained by combining two-sided t-test and average fold change to account for the observation that “ranking and selecting differentially expressed genes solely by the t-test statistic predestine a poor concordance in results” made by one of the early community-wide cross-institutional assessments of the quality of microarray data [4]. Namely, genes are ordered for differential expression by the average of their absolute values of t-statistic and fold-change ranks. Green dashed lines tend to be higher than solid green lines. This is in agreement with the fact that combining fold change and t-test tends to increase reproducibility between data sets [4]. It is also informative to notice that blue dashed lines tend to be higher than solid blue lines. That is, an increase in DEG score reproducibility tends to yield a larger WAP score reproducibility, and it remains above that of DEG scores.

Panels C and D of Fig 4 summarize score reproducibility over the six possible pairs of SSc data sets with overlap statistics maxR and AUC (as explained in “Materials and methods”). Both statistics are larger with WAP scores than DEG scores, hence demonstrating the larger reproducibility of WAP scores’ rankings over the six data set pairs. WAP scores obtained with randomly rewired networks on average tend to yield lower reproducibility than DEG scores, showing again that true biological knowledge encoded in the protein network is a likely driver of WAP score robustness. Additionally, results shown in Sections 4.3 and 4.4 of S1 Text illustrate that such higher reproducibility of the ordering of WAP scores is relatively robust with respect to: a) removal of interactions between co-expressed genes in the network, b) partial random rewiring of the network (as approximation for simultaneous introduction of both false negative and false positive interactions to the graph), and c) reproducibility of WAP findings is less sensitive to false positives as compared to false negatives randomly added to the pathway networks.

While higher reproducibility of WAP findings as compared to that of DEGs is observed with the considered SSc data sets, it is obviously not a feature universal to all possible data set pairs. Robustness actually requires that a data set contains differential expression for disease versus control which is specifically organized within the protein network. This topic will be explored later. First, higher robustness of top WAPs than that of top DEGs is shown to hold over diseases others than SSc.

### Validation of WAP score robustness in other diseases

To further evaluate robustness of the rankings of WAP scores versus those of DEG scores, a large number of data set pairs is required. There however exists only a limited number of available independent gene expression studies which focus on the same disease and the same tissue. This limitation can be alleviated by partitioning individual data sets with a sufficiently large number of samples.

Namely, a data set can be randomly split in two data sets with the goal of comparing disease and control samples. If *n* is the smallest number of disease or control samples, then a lower bound for the possible number of random partitions is *n*!^2^/(*n*/2)!^4^/2. With *n* as small as 10 this gives over 3 × 10^4^ possible splits, and thus enables reasonable statistical estimation. Note that comparing robustness of WAPs’ rankings against that of DEGs in partitions of individual data sets is actually a quite conservative approach, because reproducibility of gene expression findings is expected to be the highest within individual studies.

**Fig 5:**
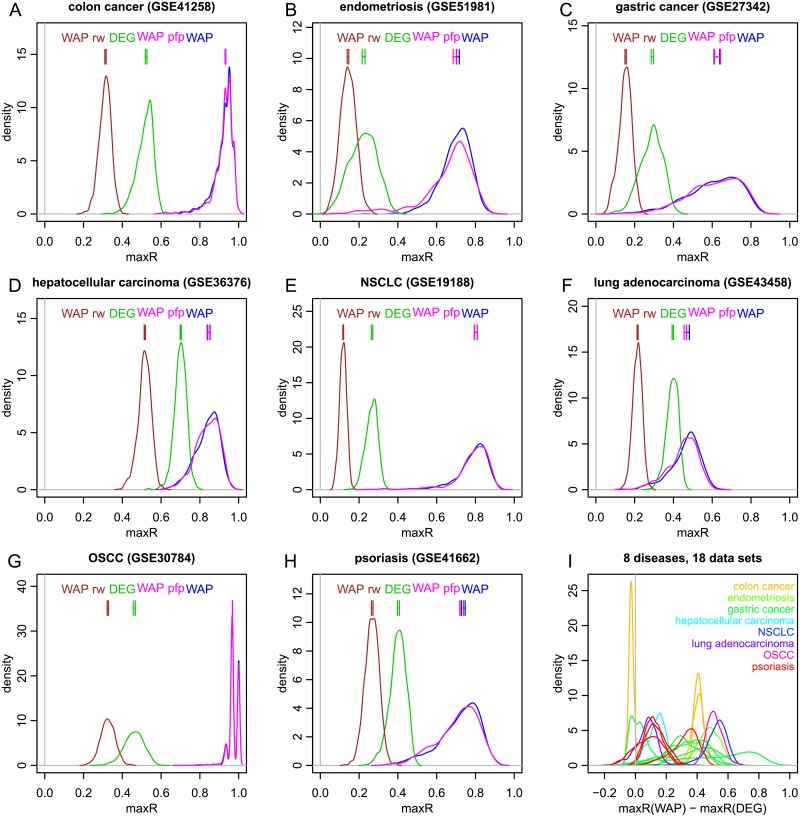
Comparison of WAP and DEG scores reproducibility in eighteen large gene expression data sets representing eight diseases. A-H: Each data set is randomly split in two sets one thousand times, and the resulting distributions of overlap statistic maxR are estimated for WAP scores (blue), PFP values of WAPs (pink), DEG scores (green) and WAP scores with randomly rewired protein network (brown). Vertical bars display 99% confidence intervals on median values estimated by bootstrapping with 10^6^ samples. I: summary of results over 18 data sets for distributions of maxR differences between DEG and WAP scores (paired values of maxR in each partition (WAP versus DEG scores)).

In the following random data-set partitions, DEGs are defined by comparing disease and control samples via a two-sided t-test. Included diseases are colon cancer (GSE41258 [60], GSE44076 [61], GSE44861 [62]), endometriosis (GSE51981 [63]), gastric cancer (GSE13195 [64], GSE19826 [65], GSE27342 [66], GSE30727 [67], GSE63089 [68], GSE79973 [69]), hepatocellular carcinoma (GSE36376 [70]), non-small cell lung carcinoma (NSCLC; GSE19188 [71]), lung adenocarcinoma (GSE43458 [72]), oral squamous cell carcinoma (OSCC; GSE30784 [73]) and psoriasis (GSE13355 [74], GSE30999 [75], GSE34248 [76], GSE41662 [76]). Distributions of overlap statistic maxR which are estimated over one thousand random partitions of each data set are displayed in Fig 5. Panels A to H detail results over eight individual data sets and diseases. Vertical bars near the top display 99% confidence intervals on median values of maxR. Reproducibility of the top WAPs (blue) tends to be on average significantly higher than that of the top DEGs (green) and approximately comparable to the reproducibility of the WAPs ordered by their PFP values (pink). Similar plots for the entire collection of the data sets cited above are shown in Section 5 of S1 Text that also includes using AUC to quantify reproducibility of top WAPs and DEGs (median Spearman correlation of maxR and AUC values for WAP score profiles over 1,000 random partitions of each dataset in two across all 22 NCBI-GEO datasets analyzed in this study is *ρ* = 0.86, interquartile range 0.27). For reference, plots shown therein also include results obtained on the network rewired to preserve on average 50% of the original edges.

As was already observed with SSc data sets, rankings of WAP scores with randomly rewired protein networks (brown) tend to have lower reproducibility than those of DEG scores. This, along with demonstrated lower informativeness of recently introduced differential expression (DE) prior [77] for predicting significant WAPs (as compared to that for DEGs—Section 7 of S1 Text), demonstrates again that true biological knowledge encoded in the protein network is required for robustness of WAP scores. Furthermore, comparison of the reproducibility of gene ranking by their WAP and DEG scores for a selected subset of these datasets that represent the same disease (and therefore are also impacted by inter-study variability) across 23 different PPI networks made available through NDEx [53–55] by Huang et al. [52] (see Section 6 in S1 Text) illustrates higher robustness of WAP score ranks as compared to that of DEGs for multiple PPI networks, especially for those with higher information content.

Fig 5I shows distributions of maxR differences (WAP maxR minus DEG maxR, values being paired in each partition) that are obtained with all eighteen data sets. Based on the raw WAP score, but not its PFP value, all data sets but one yield distributions which are shifted towards positive values, indicating higher reproducibility of WAP scores versus DEG scores. The only exception is colon-cancer data set GSE44861. Values of maxR in GSE44861 are small (less than 0.1) for both WAP scores and DEG scores. Moreover, for this data set overlap statistics on WAP scores have similar distributions with original and randomly rewired protein networks and even lower for WAP scores ordered by their PFP values (Figure 18, bottom row, in S1 Text). Differential expression in this data set is therefore not specifically organized within the protein network and the smallest PFP value is quite large (0.88). The other seventeen data sets, representing eight diseases, show that reproducibility of WAP scores tends to be on average higher than that of DEG scores. In summary, even though the random partition of a data set in two is a rather conservative control that disregards between studies variability, WAP scores tend to be on average significantly more robust than DEG scores over such partitions in seventeen data sets which represent eight diseases.

### Utility of WAP scores for small data sets

**Fig 6:**
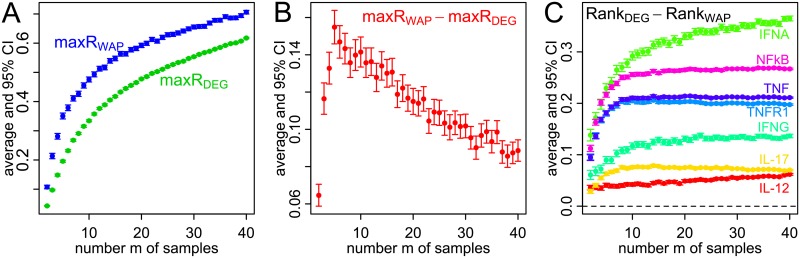
Comparison of gene expression in *m* skin samples with psoriasis lesions to *m* healthy skin samples. Statistic maxR is estimated with 10^3^ draws of two data sets of size 2*m* from GSE30999. Differences between average ranks of pathway members by WAP and DEG are estimated for 10^3^ data sets of size 2*m* randomly drawn from GSE30999. Confidence Intervals (CI, 95%, bootstrapping with 10^6^ trials, vertical lines) are estimated for average maxR values (dots) based on WAP scores, DEG scores (A), their differences (B) and average difference between pathway member ranks based on DEG and WAP scores (C). Color and labels represent the following Reactome pathways—IFNA (green): Regulation of IFNA signaling, NFkB (magenta): TNFR1-induced NFkappaB signaling pathway, TNF (dark blue): TNF signaling, TNFR1 (light blue): Regulation of TNFR1 signaling, IFNG (teal): Regulation of IFNG signaling, IL-17 (yellow): Interleukin-17 signaling, IL-12 (red): Interleukin-12 family signaling.

The potential value of the WAP score when a data set has a limited number of samples is illustrated next with Psoriasis data set GSE30999 [75], which has a rather large number of samples (83 lesions and 81 no-lesion samples). Pairs of data sets of varying sizes 2*m* (*m* lesions and *m* no-lesion) are drawn one thousand times for each value of *m*, and average values of maxR are estimated for both DEG and WAP scores. Bootstrapping with 10^6^ trials is then utilized to estimate 95% confidence intervals on these averages. Results are presented in Fig 6. Panel A shows that for large values of *m* (*m* ≥ 30) average values of maxR are quite large (e.g. at least 0.5) for DEG scores, with even larger values for WAP scores. Large DEG reproducibility is less likely to happen with small sample sizes *m*. This can be seen in Fig 6A: values of maxR become smaller for both DEG and WAP scores when *m* decreases.

Furthermore, it is instructive to examine the difference of maxR between WAP and DEG scores. Results are displayed in Fig 6B. Because WAP and DEG scores are not independent, confidence intervals on maxR differences are estimated via bootstrapping on paired values for WAPs and DEGs. One can see that even though maxR values of WAP and DEG scores decrease as *m* becomes smaller (A), the maxR difference (WAP minus DEG) instead increases until *m* becomes less than 5 (B). This clearly shows that WAP scores are less sensitive than DEG scores to reduction of sample size *m*. When DEG score reproducibility is small due to a small number of samples, WAP scores can in some cases yield a significantly more robust gene ranking than DEG scores.

Finally, it is interesting to note that for this psoriasis dataset besides achieving higher reproducibility as quantified by maxR statistic, WAP scores also result in more prominent, as compared to that by DEG, ranking of gene sets that are representative of pathways known to be involved in the psoriasis pathogenesis. Fig 6C depicts average differences between the ranks of the significance of DEG and WAP scores (where lower rank represents greater significance, i.e. smaller p-value) for the genes included in Reactome [13] pathways selected for their importance in, and history of clinical development for, this disease (e.g. signaling by TNF, IFN-gamma, IL-12, etc.) [78].

Across all sample sizes evaluated here, the average ranks of the members of these pathways are consistently lower (representing smaller, more significant, p-values) by WAP than by DEG scores. Here the rank of zero corresponds to the most significant DEG or WAP score, and the rank of one represents the least significant WAP or DEG score across the entire set of genes included in the analysis. On average, in case of this psoriasis dataset, WAP scores of the members of Reactome pathways depicted in Fig 6C rank more significantly than DEG scores by about 5% to 35% of the size of the entire gene set. Such more prominent ranking by WAP scores of pathways well recognized for their involvement for psoriasis pathogenesis further emphasizes potential merits of the WAP score for identification of new molecular targets for therapeutic intervention.

### Necessary conditions for WAP score robustness

Robustness of the gene ranking by their WAP scores is obviously not guaranteed for an arbitrary data set. Conditions which are required for robustness of WAP scores are now examined. Robustness relies on meaningful signal contained in differential expression with respect to biological information encoded in the protein network. This was first demonstrated by showing that WAP scores obtained with randomly rewired networks yield lower reproducibility than with the original network and on average lower than reproducibility of DEG scores (Figs 4 and 5). An already-introduced method for quantifying how specifically differential expression is organized within the protein network is estimation of a PFP profile (Fig 2). Intuitively, small PFP values of the top ranking WAPs suggest that the top WAPs might be robust to small changes of DEG ranks.

To test this conjecture, simulations based on SSc data set GSE58095 [43] are utilized. GSE58095 is chosen because it yields large differences in overlap statistics (maxR and AUC) between genes ordered by their WAP and DEG scores (skin samples of SSc patients versus non-SSc subjects), when this large data set is randomly partitioned in two (Section 4.4 in S1 Text). Partitions tend to yield two data sets which have small WAP PFP values, as measured for instance by the average PFP value over the top 200 WAPs. In order to explore a wider range of average PFP values, the two data sets of a partition are randomly perturbed for their composition in disease/control samples. Perturbations are controlled by a parameter 0 ≤ *ρ* ≤ 1, which can be seen as the probability of swapping disease/control state between two samples. Setting *ρ* = 0.41 yields a close to uniform distribution of PFP values averaged over the 200 top WAPs (Section 8.1 in S1 Text). With this value of *ρ*, 9 × 10^4^ pairs of data sets are sampled, so as to approximately draw 100 pairs in each cell of a 30 × 30 uniform grid. An additional 9 × 10^4^ partitions are generated with *ρ* = 1 for reasons which will be explained next, and 2 × 10^4^ partitions are also sampled with *ρ* = 0.

**Fig 7:**
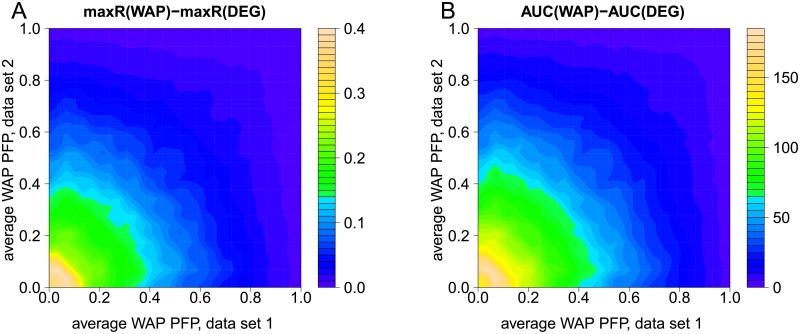
Differences in overlap statistics between WAP and DEG scores as a function of the average PFP value. A: Differences between maxR values for WAP and DEG scores. B: Differences between AUC values for WAP and DEG scores. Top 200 WAPs in each data set of a simulated pair are used to calculate maxR, AUC and PFP. Plots summarize results obtained with 2 × 10^5^ random partitions of GSE58095.

Fig 7 summarizes results obtained with the 2 × 10^5^ sampled partitions of GSE58095. On average, both overlap statistics (maxR and AUC) tend to be higher for WAP scores as compared to DEG scores, when both data sets are rich in WAPs having small PFP values (lower left corners). Absence of small PFP values in both data sets yields similar reproducibility of WAP and DEG scores (upper right corners). Notice that if one of the data sets has a small average PFP value (e.g. 0.05), then it might still yield better reproducibility of WAP scores versus DEG scores even if the second data set has a larger average PFP value (e.g. 0.3). One can therefore state that a necessary condition for larger reproducibility of WAP scores versus DEG scores between two data sets is that at least one of the data sets yields WAP scores having small PFP values. A large proportion of small PFP values indicates that observed differential expression is specifically organized within the protein network, as compared to differential expression obtained when randomly reassigning disease states to samples.

One can also characterize differential expression just for its magnitude, independently of the protein network. This can be done, for instance, with the average FDR value [42] over the top 200 DEG scores. This would however be too computationally expensive given the very large number of sampled data set pairs. Instead, a more efficient approach is based on a statistic of Pearson dissimilarities between entire samples, i.e. dissimilarities based on all genes. The statistic is called Multi-Variate T (MVT) and significance of its value is assessed via permutation testing (as explained in “Materials and methods”). Small MVT p-values correlate well with small average FDR values over the top 200 DEGs (Section 8.3 in S1 Text). The advantage of the MVT p-value is that the control distribution can be estimated once and then rapidly utilized with all simulated data set pairs (a similar approach was utilized to rapidly estimate average PFP values over the top 200 WAPs). The disease/control mixing parameter is now set to *ρ* = 1, and 9 × 10^4^ pairs of data sets are sampled. The rationale is to sample approximately 100 data set pairs in each cell of a 30 × 30 uniform grid for MTV p-values between 0 and 1 (Section 8.1 in S1 Text).

**Fig 8:**
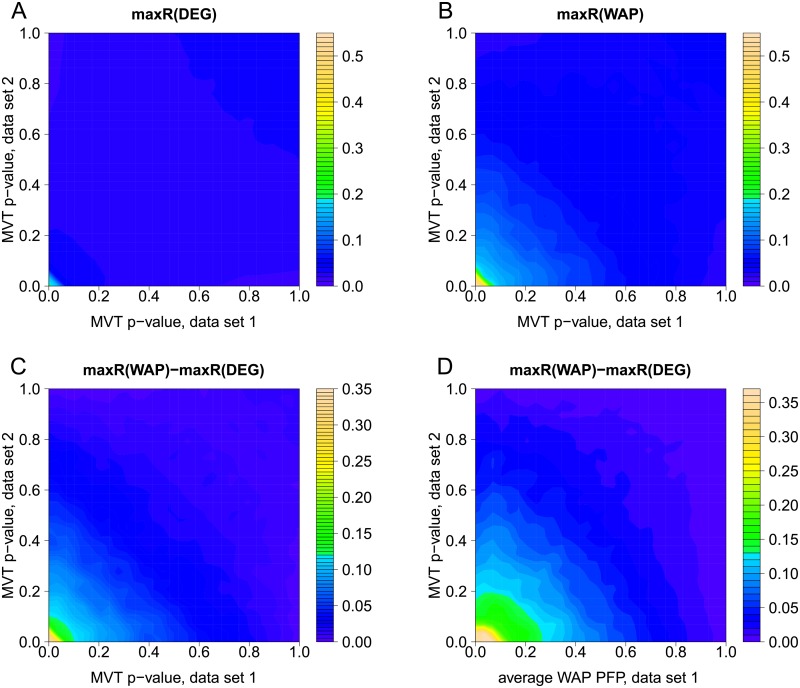
Overlap statistic maxR for DEG and WAP scores as a function of MVT p-values. A-B: Overlap statistic maxR for DEG and WAP scores as a function of MVT p-values in each data set of a simulated pair. C: Difference of maxR statistic (WAP minus DEG) as a function of MVT p-values in each data set of a simulated pair. D: Difference of maxR statistic (WAP minus DEG) as a function of MVT p-value in one data set and average WAP p-value in the other. Plots summarize results obtained with 2 × 10^5^ random partitions of GSE58095.

Average overlap statistics maxR obtained with all 2 × 10^5^ random partitions of GSE58095 are displayed in Fig 8 as a function of MVT p-values. Panel A indicates that average maxR of DEG scores never reaches values higher than 0.2. Even when the two data sets of a pair both have small MVT p-values (lower left corner), DEG reproducibility tends to remain low, and this occurs with data set pairs extracted from the same study. Fig 8B reveals a stronger effect of small MVT p-values on reproducibility of WAP findings. Average values of maxR go up to 0.55 (bottom left corner). Increase of average maxR values of WAP scores with small MVT p-values is larger than that of maxR of DEG scores, as can be seen in Fig 8C. Finally, Fig 8D illustrates that small PFP values of WAP scores are better predictors of superior robustness of top WAP ranks versus top DEG ranks than small MVT p-values.

In summary, robustness of WAP scores across data sets tends to increase when data sets exhibit significant degree of differential gene expression, as measured for instance by a small MVT p-value or a large proportion of small FDR values on DEG scores. Most importantly, higher reproducibility of top WAPs versus top DEGs is more pronounced when differential expression appears to be specifically organized within the protein network, i.e. when WAP scores yield small PFP values.

## Discussion

The importance of interactome annotation for identification and prioritization of targets for therapeutic strategies [79–81] and disease characterization [82–84] is well established with growing evidence for distinct properties of network connectivity associated with drug targets [85, 86] and disease genes [87]. The need for improving reproducibility of the findings from genome-wide studies characterizing differences between healthy state and disease remains an important analytical challenge for the field [88].

Results presented in this paper have rigorously demonstrated that utilizing prior biological knowledge in the form of known protein functional relationships can significantly enhance reproducibility of the findings from gene expression analysis, at least in the context of characterizing a disease, therefore potentially providing more robust description of the phenomenon under study. Demonstration was performed with thousands of data set pairs, which were generated with twenty-two large gene expression data sets representing nine different diseases. By scoring the same universe of genes with both WAP and DEG scores, comparison of the reproducibility of the findings by these two approaches could be made in a rigorous way. This focus on making the reproducibility of the findings made with and without prior biological knowledge in the form of the PPI network directly comparable sets it apart from other methodologies that enable analysis of genome-wide molecular characterization data with pathway networks (e.g. [23, 24] and others reviewed in [10, 19, 20]).

The method to score gene products for their association to differentially expressed genes, i.e. the WAP score, is well-conditioned for the total number of interactions of each gene. This was achieved by combining published methods [37] and novel correction factors (Section 3 in S1 Text). Besides not requiring to choose a threshold which defines differential expression, another advantage of the WAP score is that its estimation can be implemented efficiently, i.e. in O(M) where *M* is the total number of interactions in the protein network. This means that false discovery rates can be numerically estimated via permutation techniques in reasonable time. It also implies that several hundred thousand pairs of data sets can be rapidly scored, so as to provide statistical evidence for higher reproducibility of top WAPs as compared to that of top DEGs. Throughout this study, for illustration purposes, the DEGs were identified by a two-sided t-test (disease vs. healthy) for the reasons of clarity and simplicity. However, ranking of genes in protein interaction network by their WAP scores can be readily obtained for genome-wide ranking of genes by their statistical significance as estimated by more sophisticated approaches [89–91], when necessary for the studies with more complex designs. Evaluating the impact of WAP methodology on the reproducibility of the findings from such more advanced models represents one of the exciting possibilities for follow-up investigations.

Higher reproducibility of WAPs rankings as compared to those of DEGs is obviously not a given fact. Such robustness relies upon sound biological knowledge in the protein network, as attested by the fact that WAPs computed with randomly rewired networks are less reproducible than DEGs. Significant coverage of the interactome is potentially an important factor, even though reducing interactions down to a few tens of thousands having the highest confidence levels [41] can still yield robust WAP findings in the case of systemic sclerosis data sets (Section 4.2 in S1 Text). Evaluation of the impact of edge confidence score on the robustness of WAP score rankings did not yield compelling evidence for choosing a specific threshold for using a subset of STRING network for the analysis of these datasets. This in combination with the observed higher sensitivity of WAP procedure to false negatives than false positives and its tendency to result in greater gain of reproducibility for larger networks among those evaluated in [52], calls for a generic recommendation of using larger compendiums of PPI data when possible. However, when the size of gene expression data enables evaluation of its intra-study reproducibility by random partitioning, it could be worthwhile verifying the lack of drastic sensitivity of the reproducibility of the findings on the edge confidence threshold if/when available by following a procedure demonstrated in Section 4.4 in S1 Text.

Similar to the recent report [52] that surveyed available genome-wide interaction networks for their ability to recover known disease gene sets, and concluded that the larger PPI networks perform better in this context, greater gain in the reproducibility of WAP findings for larger networks was also observed here as well (Section 6 in S1 Text). Additionally, it could be instructive to evaluate relative contributions of different types and/or source of interactome annotation to WAP score robustness in the future. Preliminary work suggests that, while including co-expression knowledge in interactions is beneficial to the robustness of WAPs versus DEGs, it is unlikely to be the critical element (Section 4.3 in S1 Text). Furthermore, comparison of the rankings of the significant WAPs and DEGs by a recently introduced DE prior [77] across NCBI-GEO datasets evaluated in this study did not detect increased enrichment of WAPs for the genes that are more likely to be differentially expressed across large compendium of transcriptional profiling studies (Section 7 in S1 Text). Finally, the observed tendency of WAP scores to yield more reproducible ranking of genes as compared to that by their DEG scores has been shown to be robust to moderate amounts of random noise introduced in the protein interaction network, especially to false positives (Sections 4.4 and 5 in S1 Text) and to hold for multiple PPI networks evaluated and/or derived by [52] (Section 6 in S1 Text).

Besides being dependent on quality of protein interactions, the robustness of WAP findings also relies upon the signal encoded in gene expression data. Top WAPs are more robust when differential expression is significantly high, e.g. when it yields a large proportion of DEGs with small FDR values. More importantly, it was shown that robustness requires differential expression to be specifically organized within the protein network, i.e. it must yield small PFP values on WAP scores. When this easily-testable condition is satisfied, identified WAPs with small PFP values have the potential to be more robust than top DEGs across data sets, and this can be valuable in the case of small studies, as was demonstrated with a psoriasis data set.

Such an increase in the reproducibility of the findings from gene expression studies with smaller sample sizes by utilizing PPI information with the WAP framework may provide an appealing and cost-effective alternative to increasing reproducibility of DEGs by increasing the number of samples characterized by gene expression. The positive impact of the increase of sample size on the reproducibility of the DEGs observed in gene expression studies is well-recognized and has been extensively studied in the context of microarray and RNA-seq technology (e.g. [92, 93] and references therein). More detailed evaluation of the gains in reproducibility of the findings from gene expression findings due to the use of PPI data and WAP methodology and comparing it to that solely due to the increase in the sample size of gene expression datasets across broad number of biological phenomena and experimental designs represents another promising area of future research.

Limiting gene expression data used by WAP methodology to that for the genes represented in PPI network is an inherent source of information loss for this approach. In the light of observed lower impact of false positive interactions on the robustness of WAP findings and its tendency to yield more robust findings (as compared to those of DEGs) for larger networks, this shortcoming could be partly alleviated by using larger compendiums of PPI data for WAP analyses. Additionally, the analyses reported herein were purposely limited to the overlap of genes represented both in the gene expression data and PPI network for the reasons of clarity and direct comparability of the robustness of WAP and DEG rankings that was the main focus of this study. It is straightforward to extend WAP calculation to score nodes in pathway network that are not in gene expression data themselves and/or are not reliably detected due to low levels of expression, cross-hybridization, etc., but their network neighbors are. Such an extension, that is technically trivial, would further advance the potential of WAP methodology to reveal important biological aspects of phenomena studied in a manner complementary to the conventional differential expression analysis.

Although the comparison of disease vs. lack thereof was employed in the examples used in this study, the same methodology can be readily applied to characterization of a broader variety of biological systems for which genome-wide measurements of gene expression data and corresponding interactome information are available. For instance, it would be interesting to apply this approach to the data sets characterizing chemosensitivity of cancer cell lines [94] as recent publication by [95] suggests relevance of interactome information in this context. Findings from this type of analysis might be particularly amenable for experimental follow-up to directly test the hypothesis of high pertinence of top WAPs for the phenomenon studied. Extending WAP methodology to the generation of the predictions about individual patient outcomes, similarly to the approach presented in [24], represents another promising direction of future research.

In conclusion, this paper has rigorously demonstrated that utilizing systems-level knowledge about protein functional relationships can significantly enhance reproducibility of disease description via gene expression analysis. Such enhanced reproducibility, which soundly makes use of accumulated prior biological knowledge of diverse types, is likely to be beneficial to devise targeted therapeutic interventions, drug repurposing and potentially to benefit precision medicine investigations.

## Supporting information

S1 Text Supplementary material. Algorithmic details and numerical results further characterizing comparative reproducibility of WAPs and DEGs.
(PDF)
