# Supplementary material for: Enhancing reproducibility of gene expression analysis with known protein functional relationships: The concept of well-associated protein
Source: PLoS Comput Biol. 2020 Feb 14;16(2):e1007684. doi: 10.1371/journal.pcbi.1007684 (PMC7046299; doi:10.1371/journal.pcbi.1007684)
Supplement: S1 Text — Algorithmic details and numerical results further characterizing comparative reproducibility of WAPs and DEGs. (PDF) [file pcbi.1007684.s001.pdf]

Enhancing reproducibility of gene-expression analysis with known protein functional relationships: the concept of well-associated protein

Supplementary Material

Joël R. Pradines, Victor Farutin, Nicholas A. Cilfone, Abouzar Ghavami, Elma Kurtagic, Jamey Guess, Anthony M. Manning & Ishan Capila

February 4, 2020

Contents

1 Edge-count probabilities **2**

2 Efficient computation of association profiles **3**

3 Bias by degree **4**
3.1 Numerical results . . . 4
3.2 Poisson model to study bias by degree . . . 5
3.3 Exact calculation of $\langle \min_i P_i \rangle$ under the Poisson model . . . 7
3.4 Simple model of correction factor . . . 10
3.5 Effect of the correction for permuted and original sample annotation . . . 11

4 Systemic Sclerosis data sets **13**
4.1 Overlap statistics for WAP scores and DEG scores . . . 13
4.2 Effect of edge confidence level . . . 16
4.3 Study of co-expression effect on WAP scores and their reproducibility . . . 18
4.3.1 Introduction . . . 18
4.3.2 Co-expressed gene pairs and edges in STRING . . . 18
4.3.3 Removal of edges having highly co-expressed genes . . . 19
4.3.4 Conclusions . . . 20
4.4 Partitions of GSE58095 . . . 21

5 Cancer, endometriosis and psoriasis data sets **23**

6 Reproducibility of WAP findings across pathway networks **32**

7 Ranking of WAPs and DEGs by differential expression prior **34**

8 Necessary conditions for WAP score robustness **36**
8.1 Sampling of perturbed data set pairs . . . 36
8.2 Overlap statistics as a function of data set pair characteristics . . . 37
8.3 Correlation between MVT p-value and average gene FDR value . . . 40

1 Edge-count probabilities

Consider a large and sparse undirected graph. Large means that the graph order (number n of vertices) is at least a few thousands. Sparse means here that the average vertex degree $\langle k \rangle$ is small compared to the graph size m (total number of edges): $\langle k \rangle / m \ll 1$. Call $x_{u,i}$ the observed number of edges between a vertex v_u and a prescribed set $\mathcal{S}_i$ of i vertices. Call $x_{t,i}$ the similar quantity for another vertex v_t and $x_{u,j}$ that for another vertex set $\mathcal{S}_j$. The problem at hand is that of comparing $x_{u,i}$, $x_{t,i}$ and $x_{u,j}$.

Fair comparison should take into account vertex degrees k_t and k_u, as well as numbers of vertices in $\mathcal{S}_i$ and $\mathcal{S}_j$, and degrees of their elements. This can be achieved by replacing quantity $x_{u,i}$ with the probability of observing at least that many edges under a model of random graph which exactly preserves vertex degrees (Random Graph with Fixed Degree Sequence, or RGFDS). Namely, quantity $x_{u,i}$ is replaced with $\Pr(X_{u,i} \geq x_{u,i})$, where $X_{u,i}$ is the corresponding random variable, and the concept of probability is defined by counting over all possible realizations of the RGFDS. Individual realizations of the RGFDS can be obtained numerically by rewiring edges: two edges $\{v_a, v_b\}$ and $\{v_c, v_d\}$ are randomly selected and transformed into $\{v_a, v_d\}$ and $\{v_c, v_b\}$, provided $v_a \neq v_d$, $v_c \neq v_b$ and the new edges do not already exist in the graph. A similar numerical process was for instance utilized to identify subgraphs which are overrepresented in some networks [24]. This numerical process is however costly, as drawing one realization of the RGFDS required rewiring $10m$ pairs of edges. This is illustrated with Figure 1.

Figure 1: Relative overlap of edges between original and rewired networks (ratio of cardinalities of intersection to union of edges) as a function of the number of pairs of edges that are rewired relative to graph size m. Dots display average values over 100 random graphs and vertical lines correspond to plus/minus one standard deviation. Red curve represents local polynomial regression fit (LOESS, [8, 31]) used to estimate counts of rewired edge pairs that on average preserve approximately 80%, 50% and 20% of the original edges in the network as indicated by varying shades of blue on the plot. The network is here STRING [13] restricted to edges of confidence level $c \geq 0.7$.

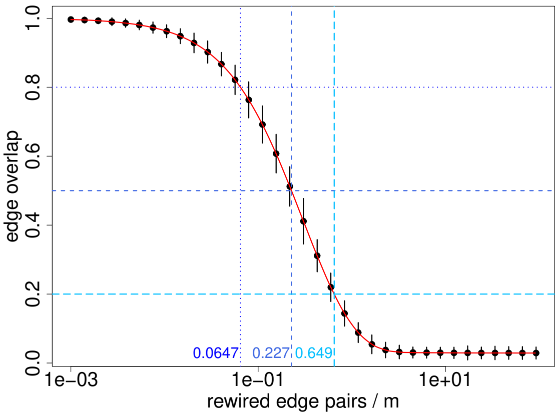

Analytical approximations for the probability or certain small subgraphs under the RGFDS have been obtained [18]. The problem of finding some of these expressions is greatly simplified by considering a different model of random graph, where vertex degrees are only preserved on average over the set of all random graph realizations. This null model of network is known as the Random Graph with Given Expected Degrees (RGGED) [7]. In this model, an edge between vertices v_t and v_u is represented by a Bernoulli random variable of parameter $p_{tu} = \min(1, k_t k_u / 2m)$. The key

advantage of the RGGED model is to treat different edges as independent random variables. This both greatly simplifies analytical treatment and acknowledges the fact that exactly preserving k_t and k_u is not the most conservative model. In the RGGED, degree of vertex v_t is now a random variable with expected value k_t and standard deviation $\sqrt{k_t}$ [28], thus introducing a reasonable uncertainty on k_t.

Call $X_{u,i}$ the RGGED random variable corresponding to association (number of edges) between vertex v_u and vertex set $\mathcal{S}_i$. This random variable is the sum of i independent Bernoulli variables, one for each possible edge. Because Bernoulli variables have small parameters, $X_{u,i}$ has approximately Poisson distribution [20] and one obtains [28, 27]:

$$\Pr\left(X_{u,i} \geq x_{u,i}\right) \simeq \frac{e^{-\lambda_{u,i}}}{\alpha_{u,i}} \sum_{h=x_{u,i}}^{i} \frac{\lambda_{u,i}^h}{h!} \quad \text{with} \quad \alpha_{u,i} = e^{-\lambda_{u,i}} \sum_{h=0}^{i} \frac{\lambda_{u,i}^h}{h!} \quad \text{and} \quad \lambda_{u,i} = \frac{k_u}{2m} \sum_{t \in \mathcal{S}_i} k_t. \quad (1)$$

Note that the upper bound i is utilized when summing Poisson terms, even if $i > k_u$. This is because vertex degrees are only fixed on average in the RGGED.

Computation of the above edge-count p-values is performed based on the following pseudo-code:

```
EDGE-COUNT-PVALUE(x, λ, z)
if z = 0
   then return 1
lp ← −λ
p ← exp (lp)
P ← 0
α ← p
if λ ≥ 100
   then t ← (x − λ) / √λ
        return GAUSSIAN-PVALUE (t)
for y ← 1 to m
     do lp ← lp + log (λ) − log (y)
        p ← exp (lp)
        α ← α + p
        if y ≥ x
          then P ← P + p
               if p/P < 10^−16
                 then break
P ← P/α
return P
```

2 Efficient computation of association profiles

Call P_i the edge-count p-value $\Pr\left(X_{u,i} \geq x_{u,i}\right)$, where $x_{u,i}$ is the observed number of edges (association) between vertex v_u and the i vertices of vertex set $\mathcal{S}_i$. Since values of P_i are conditional to all vertex degrees they can be compared, and one can state that the best association of vertex v_u to Differentially Expressed Genes (DEGs) is given by $\min_i P_i$, when DEGs are ordered by ascending differential expression.

A naive approach to compute all values of P_i for all n vertices would be $\mathcal{O}\left(n^2 m\right)$, where m is the graph size. However, computation of all P_i values for all vertices can be done in $\mathcal{O}\left(m\right)$:

```
Best-Attachment-PValues
for i ← 1 to n                                    ▷ initialization
    do P[i] ← 1
       x[i] ← 0
sK ← 0                                            ▷ running sum of degrees
for i ← 1 to n                                    ▷ all vertices
    do sK ← sK + k[i]
       for j ← 1 to |H_i|                         ▷ neighborhood of vertex i
           do x[H_i[j]] ← x[H_i[j]] + 1
              t ← sK
              z ← i                               ▷ the max attachment is |S_i|
              if H_i[j] ≤ i                       ▷ avoids counting a vertex twice
                 then t ← t − k[H_i[j]]
                      z ← z − 1
              λ ← t k[H_i[j]] / 2m
              d ← Edge-Count-PValue(x[H_i[j]], λ, z)
              if d < P[H_i[j]]
                 then P[H_i[j]] ← d
```

where $H_i[j]$ is the index of the vertex which is the jth neighbor of vertex i and $k[i]$ is the degree of vertex i.

3 Bias by degree

3.1 Numerical results

Figure 2: Average vertex degree, over a set of 10^4 random orders of DEGs, as a function of WAP score rank; the network is here restricted to edges of confidence level $c \geq 0.7$. The horizontal line corresponds to the average vertex degree.

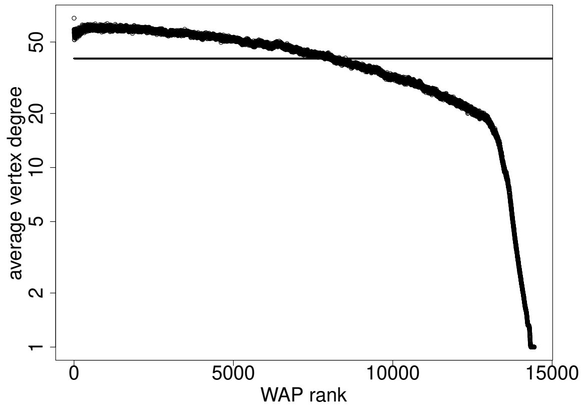

While edge-count p-values P_i are conditional to vertex degrees and thus can be compared to each other, possible values of $\min_i P_i$ are significantly affected by vertex degree. One reason for this is that the vertices with larger degrees present larger set of values to choose minimum from. For instance, a vertex of degree $k = 100$ has 100 more possible values of $\min_i P_i$ than a vertex of degree

Figure 3: Average values μ of $\min_i P_i$ (crosses), over a set of 10^4 random orders of DEGs, as a function of vertex degree k. Lines show fitted linear models for $\log \mu = f(\log k)$.

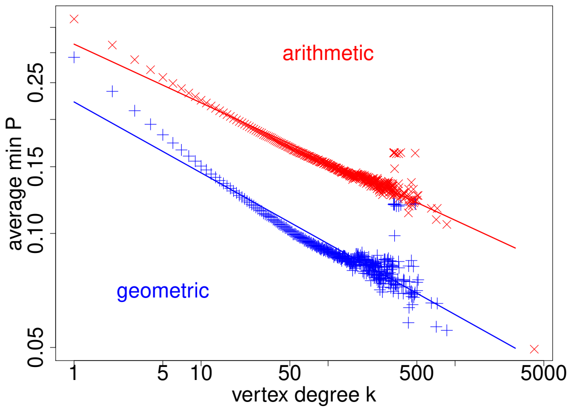

$k = 1$. Additionally, vertices with larger degrees can attain lower values of P_i than vertices with smaller numbers of neighbors on the graph. For example, for a vertex of degree $k = 1$ in the graph with m edges the lowest possible value of P_i is $1/(2m)$ when/if this vertex is connected to another vertex of degree $k = 1$ that is at the very top ($i = 1$) of the list of vertices ordered by their differential expression. For a vertex of degree k the lowest possible value of P_i is achieved when/if it is connected to k other vertices, all of degree $k = 1$, all among top k in the list of vertices ordered by their differential expression ($i = k$). In this case, such value of $P_k = \min_i P_i$ can be approximated (disregarding $e^{-\lambda_{u,k}} \approx 1$ and $\alpha_{u,k} \approx 1$ when $\lambda_{u,k} = \frac{k^2}{2m} \ll 1$) as $P_k \simeq \left[k^2/(2m)\right]^k / k!$ that even for moderate values of k is geometrically smaller than $1/(2m)$. More informally, vertices with higher degrees can potentially achieve much more striking (with respect to the RGGED null model) connectedness to the sets of other vertices as compared to the vertices with smaller numbers of neighbors in the graph.

To study this bias, vertices (DEGs) are randomly and uniformly ordered and values of $\min_i P_i$ estimated. Figure 2 shows vertex degree averaged over 10^4 random orders and as a function of WAP score rank. There is a clear correlation between WAP score rank and vertex degree, vertices with large degrees being more likely to yield small values of $\min_i P_i$.

While clearly visible with WAP score ranks, bias by degree does not result in very large difference of $\min_i P_i$ values on average. This is shown with Figure 3, which displays values of $\min_i P_i$ averaged over the same 10^4 random orders and as a function of vertex degree k. Average values vary by a factor 3 to 5 from $k = 1$ to $k \simeq 4000$. Further estimation of these factors is provided next.

3.2 Poisson model to study bias by degree

Bias of $\min_i P_i$ by vertex degree was illustrated by randomly and uniformly ordering DEGs. While this model does not preserve co-expression between genes, it is very general, i.e. data set independent. It also has the advantage of enabling fast simulations and exact calculation of average $\min_i P_i$ values.

When considering random uniform ordering of DEGs and the average value of $\min_i P_i$ over orders, the exact sequence of DEG degrees no longer matters. The probability that a vertex of degree k connects to any DEG simply becomes k/n where n is the total number of DEGs. Each potential connection can now be seen as a Bernoulli random variable of parameter $p = k/n \ll 1$. The

Figure 4: (a) Approximation of $\min_i P_i$ averaged over 10^4 random DEG orders (crosses) with simulations based on a Poisson model (dots). (b) Relative difference is defined as the ratio of absolute value difference between the two methods to their average.

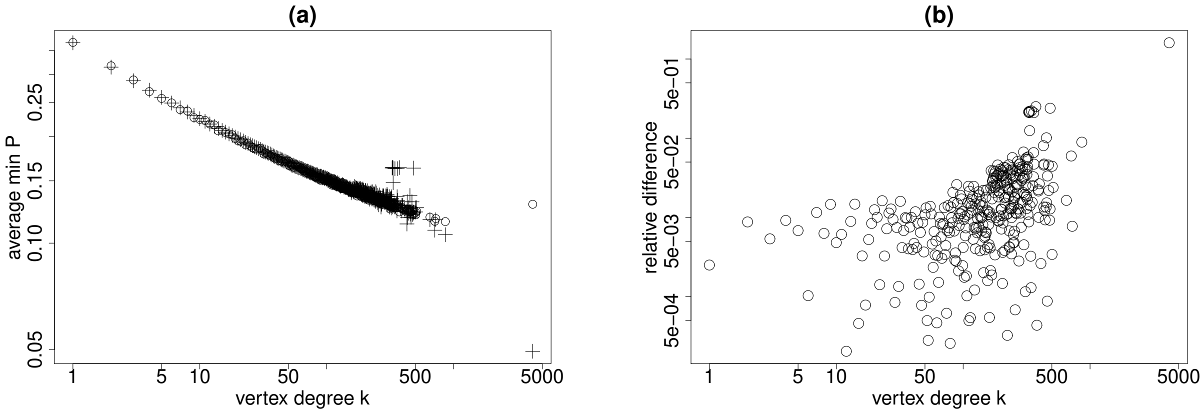

attachment X_i of a vertex of degree k to the top i DEGs is therefore modeled as the sum of i Bernoulli variables of same parameter p. This sum has probability generating function:

$$U_{X_i}(t) = (1 - p(1-t))^i \tag{2}$$

Because $p \ll 1$, $U_{X_i}(t)$ can be approximated with the probability generating function of a Poisson distribution:

$$\log(U_{X_i}(t)) = i \log(1 - p(1-t)) \simeq -\lambda_i (1-t) \quad \text{with} \quad \lambda_i = ip = ik/n \tag{3}$$

This means that the following Poisson p-value

$$\Pr(X_i \geq x) = e^{-\lambda_i} \sum_{y=x}^{i} \frac{(\lambda_i)^y}{y!} \tag{4}$$

is equivalent to edge-count probability $P_{j,i}$ (if $k_j = k$) but **only** in the context of averaging over random uniform DEG orders. The advantage of the above model is not only to simplify calculation of attachment p-values but also that it allows for fast simulation: randomly select k indices between 1 and n, sort them in increasing order $i_1 < \ldots < i_j < \ldots < i_k$ and the best attachment is $\min_j \Pr\left(X_{i_j} \geq j\right)$. All Poisson p-values can be pre-computed once in $\mathcal{O}(nk)$. The estimation of average attachment p-value with N simulations can then be done in $\mathcal{O}(Nk \log k)$, where $k \log k$ accounts for sorting of i_j values.

Panel (a) of Figure 4 visually illustrates that simulations with the Poisson model seem to provide good approximations of average $\min_i P_i$ values, except for very large degrees k. Panel (b) provides a more quantitative assessment. The absolute value of the difference between the two estimates of average $\min_i Pi$ over 10^4 random orders is divided by the average of the two estimates and displayed as a function of vertex degree k. One can see that relative differences based on 10^4 simulations are of the order of 1% when $k \leq 500$. This result is in agreement with the hypothesis that the Poisson model provides good approximations of average $\min_i P_i$ values when DEGs are randomly and uniformly ordered. Under the Poisson model it is also possible to exactly estimate values $\langle \min_i P_i \rangle$ of average $\min_i P_i$.

3.3 Exact calculation of $\langle \min_i P_i \rangle$ under the Poisson model

Figure 5: Illustration of the calculation of $\langle \min_i P_i \rangle$ for $k = 2$. Left: $\min_i P_i$ occurs at position i_1 and for the first edge. For this to happen, the second edge must occur at position $i_2 \geq b_2$, where b_2 is the smallest value of i such that $\Pr(X_{b_2} \geq 2) > \Pr(X_{i_1} \geq 1)$. Bound b_2 therefore determines the number of graphs for which $\min_i P_i$ can occur at $i = i_1$. Right: for $\min_i P_i$ to occur at position i_2 and for the second edge, the first edge must occur at $i \geq b_1$, where b_1 is the smallest integer such that $\Pr(X_{b_1} \geq 1) > \Pr(X_{i_2} \geq 2)$.

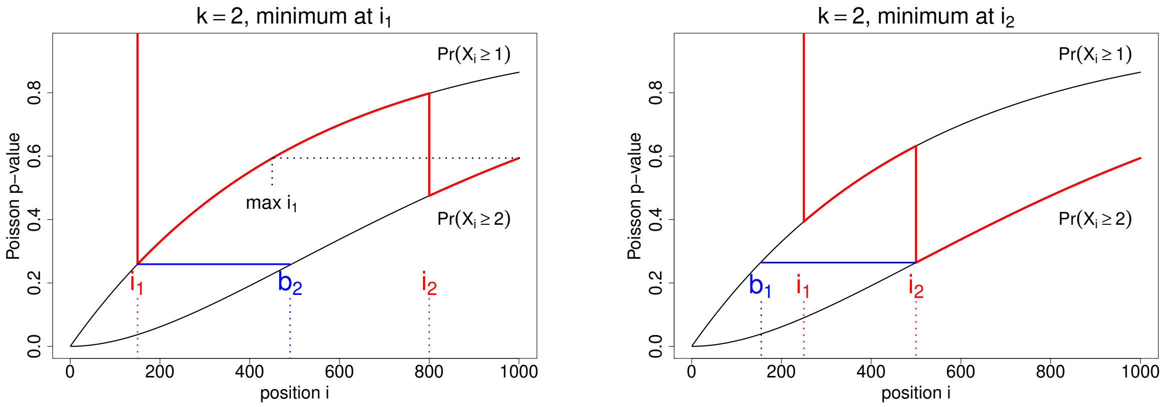

For vertex degree $k = 1$, the average value of $\min_i P_i$ is

$$\begin{aligned} \left\langle \min_i P_i \right\rangle &= \frac{1}{n} \sum_{i=1}^{n} \Pr(X_i \geq 1) \\ &= \frac{1}{n} \sum_{i=1}^{n} \left(1 - e^{-i/n}\right) \\ &= \frac{e^{-1/n}}{n} \frac{1 - e^{-1}}{1 - e^{-1/n}}. \end{aligned}$$

For $k \geq 2$, calculation of $\langle \min_i P_i \rangle$ becomes more complex. This is first illustrated with $k = 2$ and Figure 5. The strategy consists in enumerating the number w of graphs for which $\Pr(X_{i_x} \geq x)$ corresponds to $\min_i P_i$ for $x = 1$ or 2 and for all possible edge positions i_x. The left panel of Figure 5 illustrates the case where $\min_i P_i$ occurs at position i_1 and for the first edge. For i_1 to correspond to a minimum, the second edge must occur at position $i_2 \geq b_2$, where b_2 is the smallest integer such that $\Pr(X_{b_2} \geq 2) > \Pr(X_{i_1} \geq 1)$. Because $\Pr(X_i \geq 2)$ is a monotonic function of i, bound b_2 can be efficiently found via binary search in $\mathcal{O}(\log n)$. Note that if binary search returns $b_2 > n - 1$, it means that i_1 cannot correspond to $\min_i P_i$, and the number of graphs realizing a minimum at i_1 is then $w = 0$. Otherwise, the number of graphs is $w = n + 1 - b_2$, and the contribution to $\langle \min_i P_i \rangle$ via i_1 is $w \Pr(X_{i_1} \geq 1)$. The right panel of Figure 5 depicts the case where $\min_i P_i$ occurs at position i_2 and for the second edge. For the minimum to occur at i_2, the first edge must occur at position i_1 with $b_1 \leq i_1 < i_2$ and where b_1 is the smallest integer such that $\Pr(X_{b_1} \geq 1) > \Pr(X_{i_2} \geq 2)$. The value of b_1 can be found via binary search in $\mathcal{O}(\log n)$. The contribution of i_2 to $\langle \min_i P_i \rangle$ is then $w \Pr(X_{i_2} \geq 2)$ with $w = i_2 - b_1$. Performing these operations for all possible values of i_1 and i_2, and dividing by the sum of weights w yields $\langle \min_i P_i \rangle$.

For $k > 2$ the problem is summarized by the following array:

$$\begin{array}{llllll}
1 & \Pr(X_1 \geq 1) & & & & \\
2 & \Pr(X_2 \geq 1) & \Pr(X_2 \geq 2) & & & \\
\vdots & \vdots & & & & \\
k & \Pr(X_k \geq 1) & \dots & \Pr(X_k \geq j) & \dots & \Pr(X_k \geq k) \\
\vdots & \vdots & & & & \\
i & \Pr(X_i \geq 1) & \dots & \Pr(X_i \geq j) & \dots & \Pr(X_i \geq k) \\
\vdots & \vdots & & & & \\
n & \Pr(X_n \geq 1) & \dots & \Pr(X_n \geq j) & \dots & \Pr(X_n \geq k)
\end{array}$$

The array can be computed in approximately $\mathcal{O}(nk)$ by utilizing the fact that $\Pr(X_i \geq j-1) = \Pr(X_i \geq j) + e^{-\lambda_i}\lambda_i^{j-1}/(j-1)!$. Binary search can be utilized within columns since values are strictly increasing when going down.

Say event i_j (j^{th} edge at position i) yields $\min_i P_i$. As previously illustrated for $k = 2$, this can happen only under certain conditions. Going left ($j \leftarrow j-1$) and up ($i \leftarrow i-1$) we must be able to find a bound b_{j-1} on i: the largest positive integer such that $\Pr\left(X_{b_{j-1}} \geq j-1\right) > \Pr\left(X_{i_j} \geq j\right)$. If such a bound does not exist, then i_j cannot realize a minimum. Bounds need to be found for $j-1, j-2, \dots 1$. Likewise, going right ($j \rightarrow j+1$) and down ($i \rightarrow i+1$), we look for bounds $b_{j+1}, b_{j+2}, \dots, b_k$. Again, if they do not exist, then i_j cannot realize a minimum. Because of monotonicity within columns, binary search is utilized and the cost is $\mathcal{O}(\log n)$ for each bound. So, finding all bounds is $\mathcal{O}(nk\log n)$. Given i_j, estimated bounds yield a set of conditions on edge positions I_l:

$$\begin{array}{l}
I_1 \geq b_1, I_2 \geq b_2, \dots, I_{j-1} \geq b_{j-1}, I_j = i_j, I_{j+1} \geq b_{j+1}, \dots, I_k \geq b_k \\
\text{with } I_1 < I_2 < \dots < I_{j-1} < i_j < I_{j+1} < \dots < I_k
\end{array}. \tag{5}$$

The remaining task is then to efficiently enumerate the number of graphs which satisfy these conditions.

As a first example, consider bounds for minimum at i_1 with $k = 3$, $b_2 = 1$, $b_3 = 4$ and $n - i_1 = 8$, and where values of b_2 and b_3 are relative to i_1. Enumeration is summarized by the following array:

			3	7	12	18	25
1	2	3	4	5	6	7	
b_2			b_3				

			$f(2,4)$	$f(2,5)$	$f(2,6)$	$f(2,7)$	$f(2,8)$
$f(1,1)$	$f(1,2)$	$f(1,3)$	$f(1,4)$	$f(1,5)$	$f(1,6)$	$f(1,7)$	
b_2			b_3				

Enumeration goes from left to right and bottom to top, calling $f(j,i)$ the total number of graphs, given i_1, that can be created up to position i from $b_2 - 1$ and with j edges. For $j = 2$ and $i = 1$ there is only one graph. For $j = 2$ and $i = 2$ there are two graphs... up to $i = 4$ there are 4 graphs with only one edge ($j = 2$). But then the third edge might appear at $i = 4 = b_3$, if so the second edge must have been made before, so that the number of possibilities is $f(3,4) = f(2,3) = 3$. At $i = 5$ the third edge might also appear, in which case the number of possible graphs is $f(2,4)$ and the total number of graphs is $f(3,5) = f(3,4) + f(2,4)$.

As a second example, consider now the same problem with one more edge and bound: $k = 4$, $b_2 = 1$, $b_3 = 4$, $b_4 = 6$ and $n - i_1 = 9$:

					7	19	37	62
			3	7	12	18	25	
1	2	3	4	5	6	7		
b_2			b_3		b_4			

More generally, the total number $f(j,i)$ of graphs which can be created up to position i from $b_2 - 1$ and with j edges is given by the recurrence

$$f(j,i) = f(j,i-1) + f(j-1,i-1), \text{ with } f(1,i) = i \text{ and } f(j,i) = 0 \text{ if } i \le b_j. \tag{6}$$

Applying it up to $i = n - b_2 + 1$ yields the number w_r of possible graphs on the right of position i. The same method is utilized to calculate the number w_l of graphs on the left by replacing the first bound with b_1 and n with i, and the number of graphs is $w = w_l w_r$.

Figure 6: Comparison of calculated (c) and simulated (s) average values of $\min_i P_i$ under the Poisson model (10^6 simulations for each vertex degree k and $n = 10^4$). (a) visual comparison. (b) relative error $r = |c - s|/c$ as a function of vertex degree k.

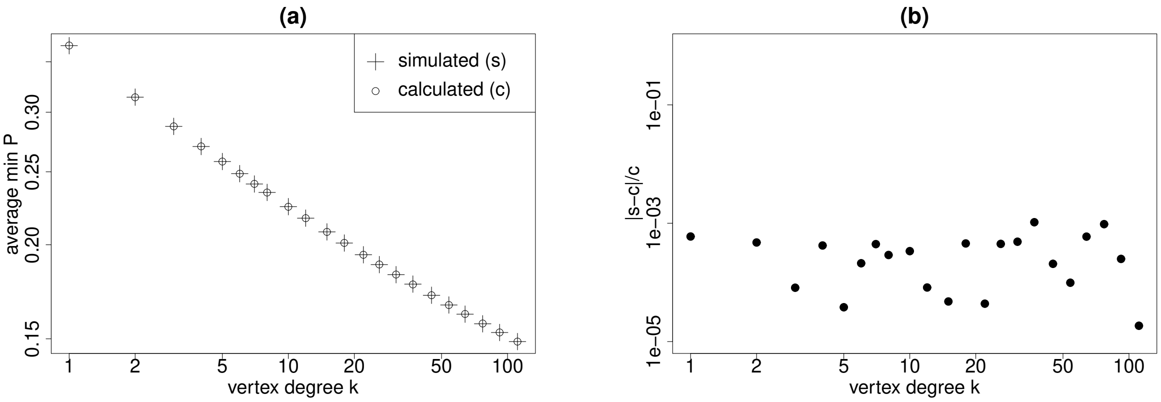

Figure 6 compares values of average $\min_i P_i$ values estimated with 10^6 simulations of the Poisson model (s) for each vertex degree k to values calculated (c) by enumerating graphs. Both sets of values are in good agreement, since the relative error $r = |s - c|/c$ (panel (b)) tends to be less than 10^{-3}, which is the expected standard deviation for estimations based on 10^6 simulations.

Exact calculation of $\langle \min_i P_i \rangle$ under the Poisson model is approximately $\mathcal{O}(n^2 k^2)$ and thus becomes prohibitive for $n > 100$ and $k > 100$.

There is another way of exactly calculating the number w of graphs given bounds $b_1, b_2, \ldots, b_k$. This method is based on combinatorics and briefly explained as follows. Call z_l the number of edges between bounds b_l and b_{l+1}, with $l < k$. Call $F(l,j)$ the number of possible graphs with j edges and with l bounds after b_1, i.e. up to b_{l+1} with $b_{k+1} = n$. Then, it can be shown that

$$F(l,j) = \sum_{\substack{z_1 + \ldots + z_l = j \\ 0 \le z_1 + \ldots + z_r \le r \\ 1 \le r \le l-1}} \prod_{r=1}^{l} \binom{b_{r+1} - b_r}{z_r}. \tag{7}$$

Quantities $F(l,j)$ can then be shown to obey the following recurrence relation:

$$F(l,j) = \sum_{r=0}^{j} F(l-1, j-r) \binom{b_{l+1} - b_l}{r}. \tag{8}$$

Because there must be at least one edge to a vertex in in $\{b_k, \ldots, n\}$, the total number of graphs given bounds $b_1, \ldots, b_k$ is:

$$w(k) = \sum_{r=1}^{k} F(k-1, k-r) \binom{n - b_k}{r}. \tag{9}$$

It can be shown calculation of $w(k)$ is of the order of $\mathcal{O}(k^3)$.

Figure 7: Linear model for $\langle \min_i P_i \rangle$ correction based on 10^6 simulations of the Poisson model. (a) Utilized degrees k (dots) and linear models (lines) of $\log(\langle \min_i P_i \rangle)$ as a function of $\log k$ for $n = 1000, 2000, \ldots, 20000$. (b) values of linear model R^2 as a function of n (dots) and numbers of different degrees k used for fitting.

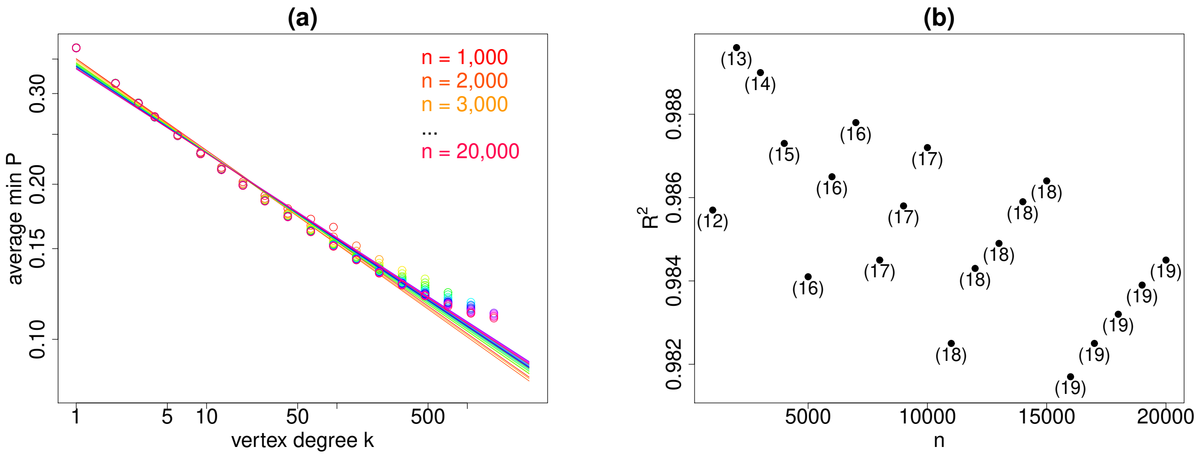

3.4 Simple model of correction factor

Since computation of exact values of $\langle \min_i P_i \rangle$ under random and uniform ordering of DEGs remains computationally expensive for large values of k even under the Poisson model ($\mathcal{O}(n^2k^2)$ or $\mathcal{O}(k^3)$), the choice is made to provide a simple linear model approximation, which is estimated with 10^6 simulations of the Poisson model for each utilized pair (n, k).

Values of n vary from 1,000 to 20,000 by step of 1,000. Values of k are 1,2,3,4 and then are based on a geometric sequence of factor $\rho = 1.5$, i.e. $k_{i+1} = \lfloor \rho k_i \rfloor$. Given n, only values of k such that $k/n < 0.1$ are utilized, so that numbers k values vary from 12 to 19.

Panel (a) of Figure 7 shows utilized values of k and n, and displays estimated values of $\langle \min_i P_i \rangle$ (dots). Lines correspond to fitted linear models of $\log(\langle \min_i P_i \rangle)$ as a function of $\log k$. Estimated parameter values are given in table 1. Panel (a) of Figure 7 suggests that linear models provide good approximations of $\log(\langle \min_i P_i \rangle)$.

Table 1: Parameters of fitted linear models of $\log(\langle\min_i P_i\rangle)$ as a function of $\log k$ and for different values of n.

n	α	β	R^2	$\max k$
1000	-0.1779	-1.048	0.9857	94
2000	-0.1799	-1.049	0.9896	141
3000	-0.1778	-1.056	0.9890	211
4000	-0.1742	-1.064	0.9873	316
5000	-0.1699	-1.074	0.9841	474
6000	-0.1720	-1.071	0.9865	474
7000	-0.1734	-1.068	0.9878	474
8000	-0.1687	-1.079	0.9845	711
9000	-0.1699	-1.077	0.9858	711
10000	-0.1709	-1.075	0.9872	711
11000	-0.1658	-1.087	0.9825	1066
12000	-0.1668	-1.085	0.9843	1066
13000	-0.1675	-1.083	0.9849	1066
14000	-0.1680	-1.082	0.9859	1066
15000	-0.1686	-1.081	0.9864	1066
16000	-0.1634	-1.094	0.9817	1599
17000	-0.1640	-1.093	0.9825	1599
18000	-0.1645	-1.092	0.9832	1599
19000	-0.1649	-1.091	0.9839	1599
20000	-0.1656	-1.090	0.9845	1599

This is confirmed by panel (b), which shows that values of R^2 are all greater than 0.981. Note that for $n \geq 2000$ values of R^2 follow a regular pattern when n or the number m of utilized vertex degrees increase. Increasing m decreases R^2, i.e. introduces more deviation from a linear model. However, at a given value of m, increasing n increases R^2. From this one concludes that deviation from a linear model is caused by large values of k/n.

Based on the above it is reasonable to utilize the linear model estimated for $n = 2000$. This model provides conservative (small) values of $\log(\langle\min_i P_i\rangle)$ for large values of k. Values of $\langle\min_i P_i\rangle$ estimated under the linear model approximate values that are expected under random uniform order of the DEGs. Therefore, an actual observed value of $\min_i P_i$ can be corrected as follows

$$\begin{aligned} &\min_i P_i \leftarrow \min_i P_i \times c(k)/c(1), \quad \text{with} \;\; c(k) = \left(k^\alpha \times e^\beta\right)^{-1}, \\ &\alpha = -0.1799, \quad \text{and} \;\; \beta = -1.056. \end{aligned} \tag{10}$$

Values of $\langle\min_i P_i\rangle$ estimated with 10^4 random uniform orders of DEGs and after correction (10) are displayed with blue dots as a function of vertex degree k in Figure 8. Unlike values without correction (red crosses), corrected values change very little with vertex degree k. One can discern the slight over-correction for values of k near 500, which is caused by the approximation via a linear model.

3.5 Effect of the correction for permuted and original sample annotation

To demonstrate numerically the utility of the correction factor, average ranks of $\min_i P_i$ with and without correction have been calculated for all NCBI-GEO datasets that were analyzed herein (as described in Sections 4 and 5) under the null model of randomizing the assignment of sample annotation to gene expression profiles. Unlike the model randomizing order of DEGs, the randomization of sample annotation preserves correlation structure of gene expression data and represents

Figure 8: Values of $\langle \min_i P_i \rangle$ estimated with 10^4 random uniform orders of DEGs with (blue dots) or without (red crosses) correction (10) and as a function of vertex degree k.

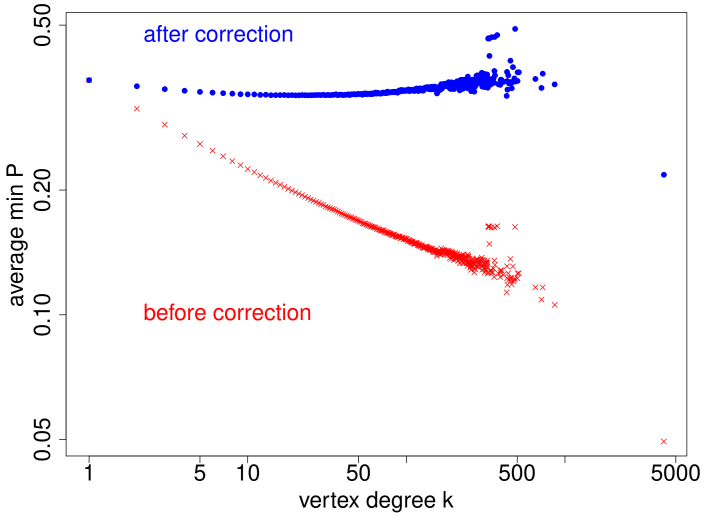

permutation test under the assumption of interchangeability of the individual samples. Therefore, average ranks of genes or vertices tested under this permutation are expected to be close to 0.5 (for ranks scaled to be between 0 and 1) for well-behaving methods and deviations from this value indicate genes or vertices that tend to be scored higher or lower than average in the comparisons of random sets of samples.

Figure 9 a) depicts average ranks of genes and vertices across 22 NCBI-GEO datasets evaluated in this study comparing random groups of samples in each dataset (10^4 sample permutations per dataset) binned by their degree on the network. Genes and vertices were ranked both by their differential expression scores regardless of their network connectivity (DEG) and by $\min_i P_i$ with and without correction for the vertex degree using the model introduced above. Ranks of zero and one represent respectively the most and the least significant $\min_i P_i$ or DEG scores. As expected, average ranks of genes scored only by their differential expression (DEG) between random groups of samples are close to 0.5 regardless of their degree in the pathway network. Genes (vertices) ranked by their $\min_i P_i$ without the correction for vertex degree manifest clearly discernible bias with the vertices of degree $k = 1$ on average falling near the least significant tertile or quartile of the ranked list, while the vertices with high numbers of neighbors on average falling near the most significant tertile or quartile. The correction for vertex degree markedly alleviates this bias with the average ranks now falling within 0.45 – 0.55 range regardless of the vertex degree so that the ranking of the genes by their $\min_i P_i$ when comparing random sets of samples is far less influenced by their degree.

Despite this systematic effect of vertex degree on average rank of $\min_i P_i$ value under the null model of randomly scrambled sample annotation, the genes that are scored the most prominently in the original comparison of healthy and diseased samples in the NCBI-GEO datasets analyzed in this study tend to appear in approximately the same order when sorted by the corrected and uncorrected values of $\min_i P_i$. Figure 9 b) depicts the ranks of $\min_i P_i$ as ordered by their corrected (Y axis) vs. uncorrected (X axis) values. Ranks of zero and one represent the lowest and the highest values of $\min_i P_i$ respectively. Ranks shown in Figure 9 b) are pooled over 22 NCBI-GEO datasets used in this publication – the top 1-10% of the most striking WAPs appear nearly in the

Figure 9: Ranks of $\min_i P_i$ with and without correction for degree bias in NCBI-GEO datasets. Ranks of zero and one correspond to the lowest and highest values of $\min_i P_i$ respectively. Error bars represent standard deviations across all datasets of the within-dataset averages of ranks binned by their degree. (a) Average $\pm$ standard deviation of gene ranks binned by vertex degree for two-groups comparison with randomized sample annotation (10^4 permutations). (b) Ranks of corrected versus uncorrected $\min_i P_i$ pooled over the same datasets with the original sample annotation. Lighter color indicates higher density of the points in the plot.

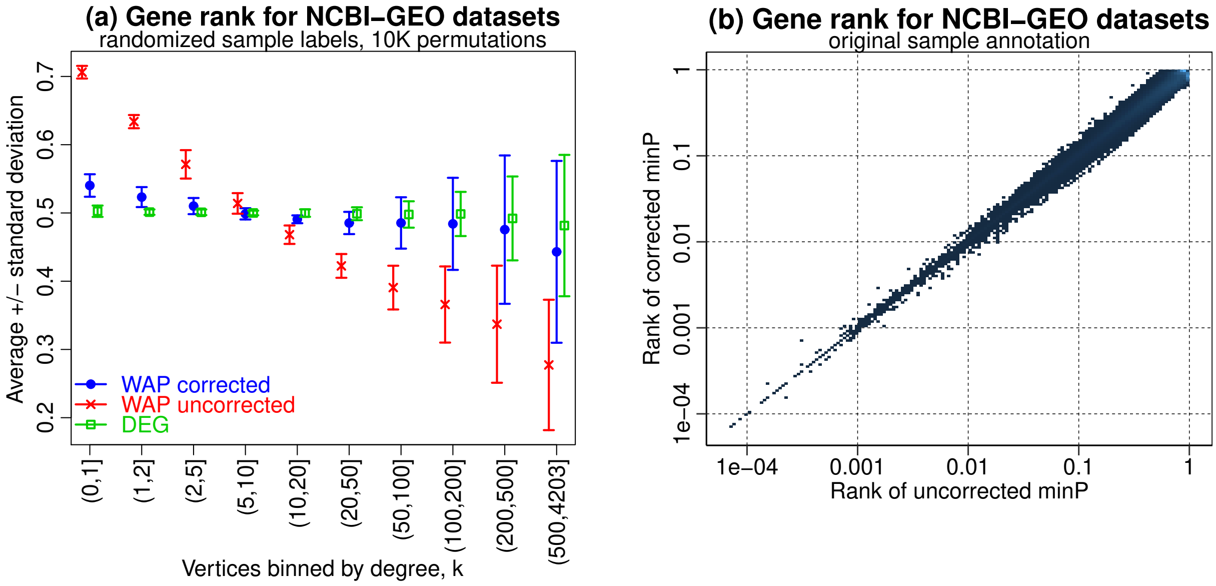

same order regardless of whether corrected or uncorrected $\min_i P_i$ are used to sort them.

4 Systemic Sclerosis data sets

Four published studies, where gene expression in skin samples is compared between Systemic Sclerosis (SSc) patients and non-SSc subjects, were utilized: GSE58095 [1] (1), GSE32413 [25] (2), GSE9285 [23] (3) and GSE45485 [15] (4).

4.1 Overlap statistics for WAP scores and DEG scores

Overlap statistics maxR and AUC for DEG scores, WAP scores and WAP scores with randomly rewired networks are displayed in Figure 10 for the six resulting pairs of data sets. Both statistics maxR and AUC are higher with WAP scores as compared to DEG scores. WAP scores based on randomly rewired networks tend to have lower reproducibility than DEG scores.

Overlap profiles over the six data set pairs are displayed in Figure 11. Solid lines correspond to DEGs ordered by descending values of absolute t-statistic (SSc versus control). Reproducibility of WAP scores (blue) is higher than that of DEG scores. Reproducibility of WAP scores obtained with randomly rewired protein networks (brown) tends to be lower than that of DEG scores. Dashed lines correspond to DEGs ordered by the average of two ranks: that of the absolute value of the t-statistic and that of the absolute value of the log-ratio (fold change) between the two group means.

Figure 10: Overlap statistics maxR and AUC for WAP scores, DEG scores and WAP scores with randomly rewired networks and over six pairs of data sets (four SSc studies).

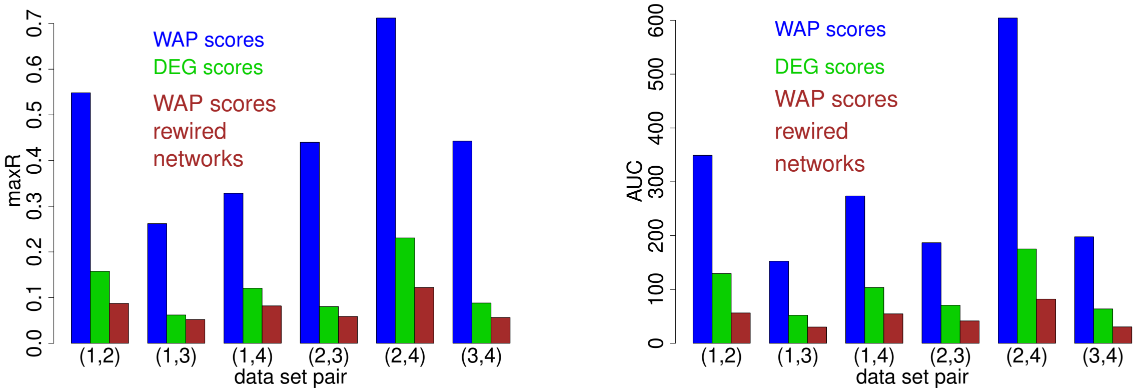

As was already shown in [22], taking into account fold change tends to improve reproducibility of DEG scores: green dashed lines tend to be above solid ones. Notice that, qualitatively, WAP scores also tend to become more reproducible when taking into account fold change (blue dashed lines), and their reproducibility remains higher than that of DEGs.

Figure 11: Overlap profiles for six pairs of SSc data sets. Blue is for WAP scores, green for DEG scores and brown for WAP scores with randomly rewired protein networks. Solid lines correspond to DEGs ranked by the absolute value of a t-statistic for SSc versus control. Dashed lines correspond to DEGs ordered by the average of absolute t-statistic value and fold change ranks.

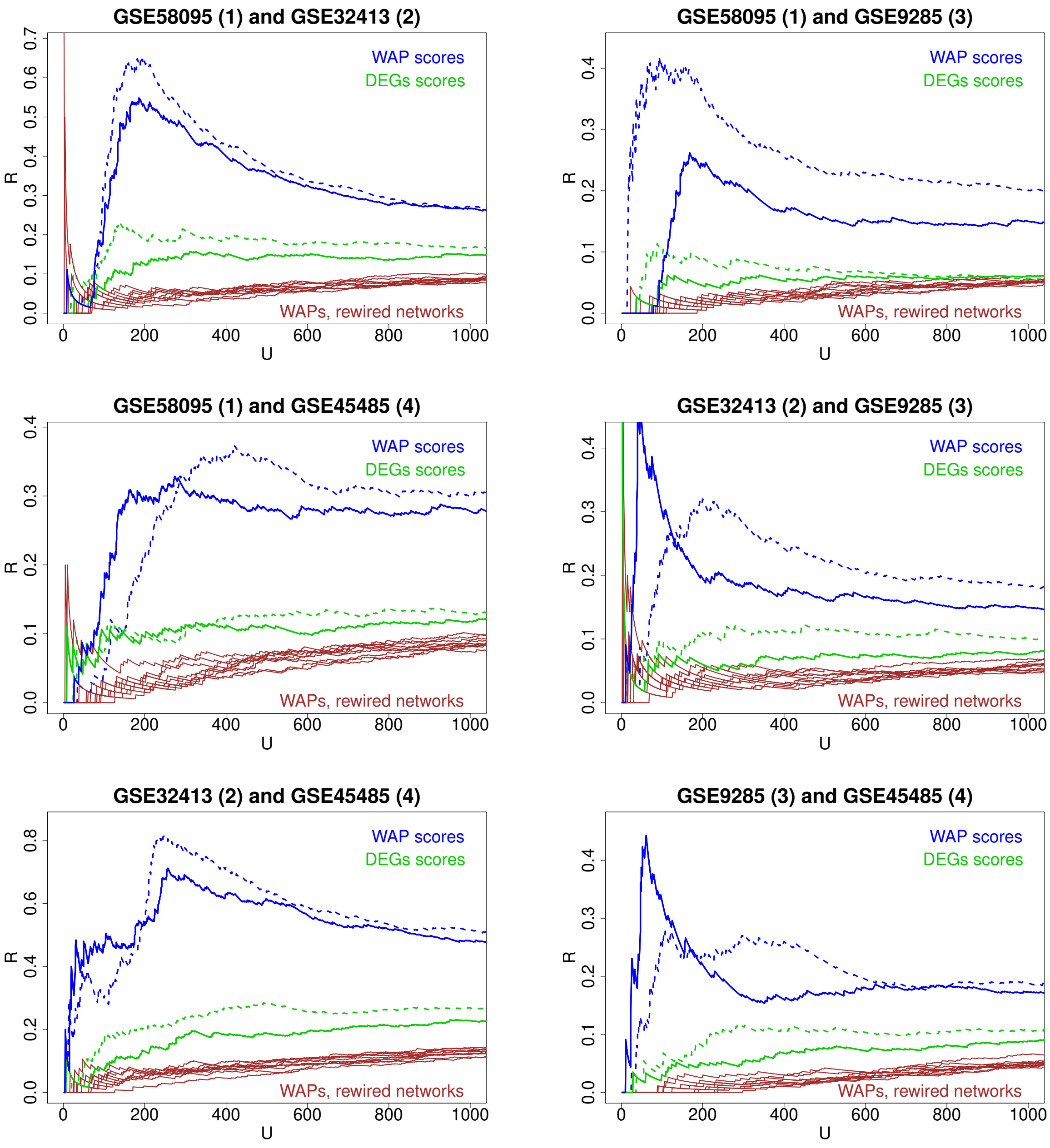

4.2 Effect of edge confidence level

Figure 12: Overlap statistic maxR for WAP (blue) and DEG (green) scores as a function of lower bound θ on edge-confidence level c of utilized STRING edges. Results are displayed for six pairs of SSc data sets.

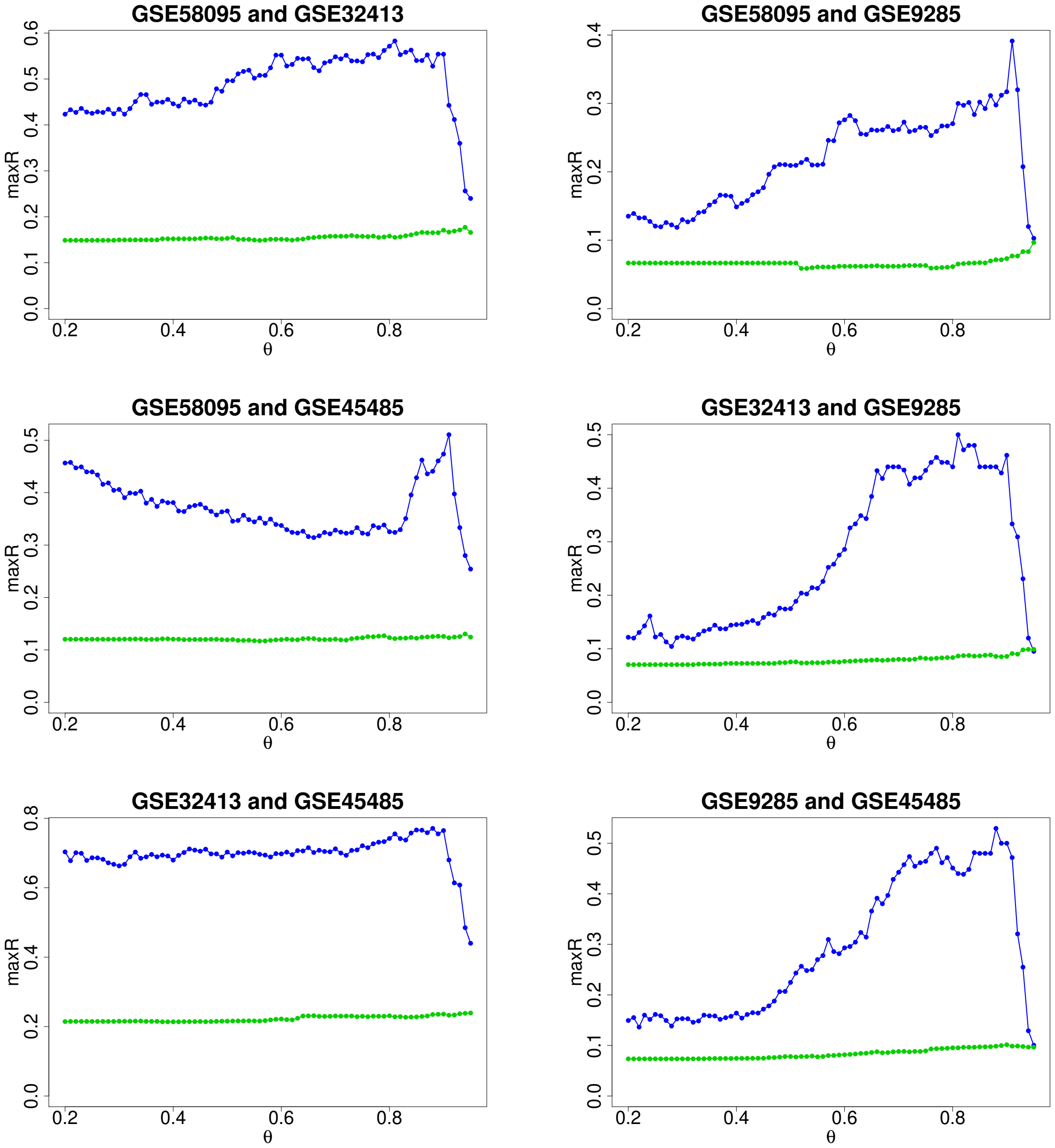

Interactions in STRING [13, 39] are provided with a confidence level $0.15 \leq c \leq 1$ [41], where 1 corresponds to the highest likelihood of an interaction. The default utilized protein network is extracted from STRING by keeping only interactions with $c \geq \theta$ and $\theta = 0.7$. Figures 12 (statistic maxR) and 13 (statistic AUC) show that superior robustness of WAP scores versus DEG scores is robust to changing the value of θ, for the considered six pairs of SSc data sets.

The dependency of reproducibility metrics maxR and AUC on the value of θ used as a threshold

Figure 13: Overlap statistic AUC for WAP (blue) and DEG (green) scores as a function of lower bound θ on edge-confidence level c of utilized STRING edges. Results are displayed for six pairs of SSc data sets.

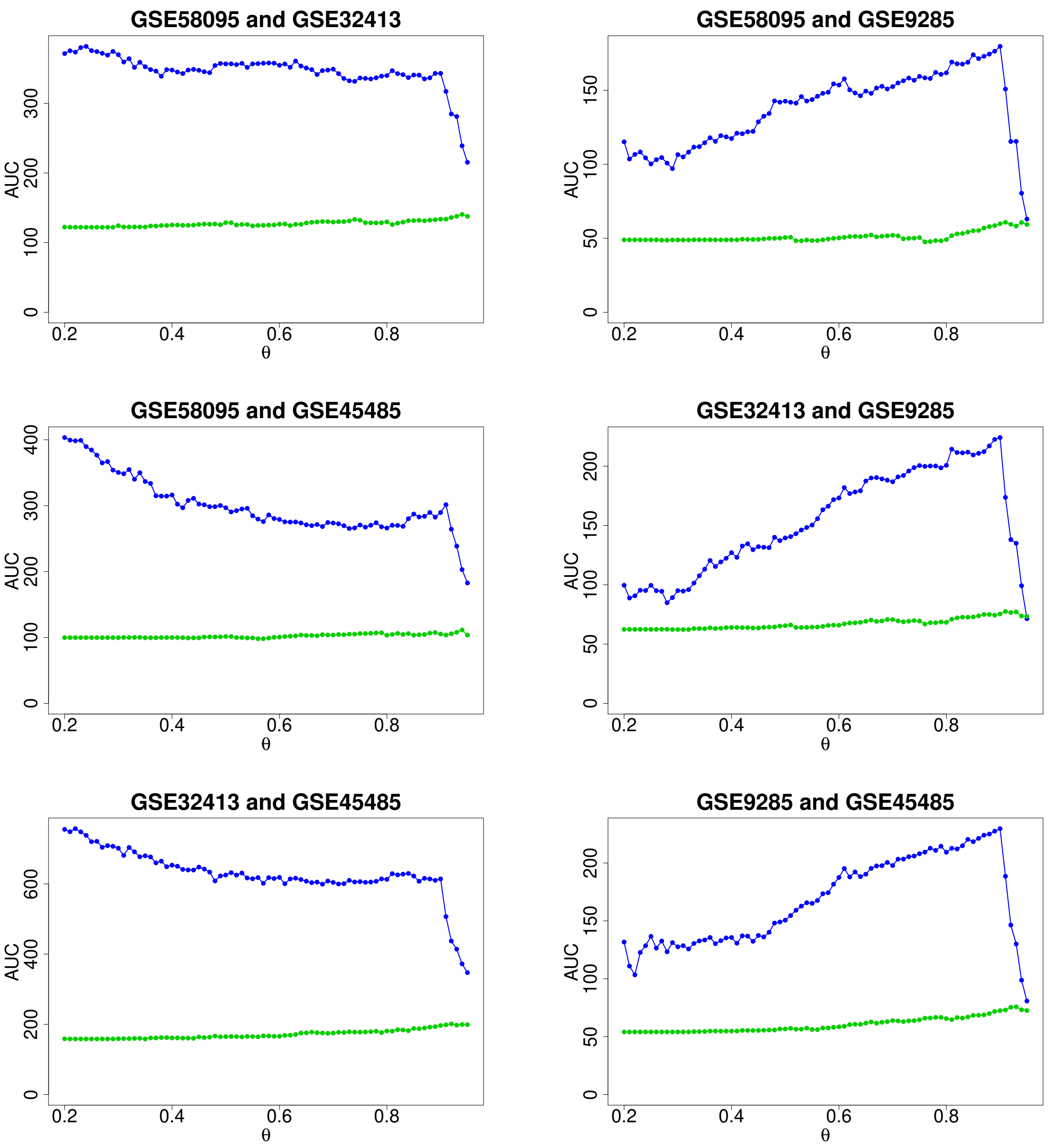

appears to be dependent on the datasets compared. For instance, for the three comparisons involving dataset GSE9285 (right columns in Figures 12 and 13) the values of both AUC and maxR increase with the increase in θ from about 0.2 to about 0.8, while for the remaining three pairs of datasets (left columns in Figures 12 and 13) the corresponding change is less pronounced or in the opposite direction. (The drastic decrease in AUC and maxR values at the highest end of the θ values regardless of the datasets involved likely reflects rapid change of the network size where change from $\theta = 0.9$ to 0.95 corresponds to approximately five-fold decrease in the number of edges in the graph – from about 200K to 40K edges respectively.)

4.3 Study of co-expression effect on WAP scores and their reproducibility

4.3.1 Introduction

Part of the evidence accumulated for interactions represented in STRING [41] is based on co-expression of genes across gene-expression data sets deposited in the Gene Expression Omnibus database [2]. It is informative to examine whether such information plays a crucial role in enhancing reproducibility of WAP scores versus DEG scores or not. It is shown below that, while co-expression is likely to contribute to WAP score robustness, it is unlikely to be a determinant factor. Evidence is provided by utilizing data set GSE58095 [1]: comparison of skin samples of SSc patients and non-SSc subjects.

4.3.2 Co-expressed gene pairs and edges in STRING

The utilized STRING network is that with edges of confidence level $c \geq 0.7$. Genes are restricted to those represented both in GSE58095 and the protein network. The resulting network has $n = 14,439$ gene products and $m = 292,128$ edges. All $n(n-1)/2$ gene pairs are ordered by decreasing absolute value of covariance across GSE58095.

Figure 14: Representation of STRING edges among the most co-expressed gene pairs in GSE58095. (a) Representation of any STRING edge. (b) Edges are restricted to those of the top 418 WAPs in GSE58095 (PFP < 0.1).

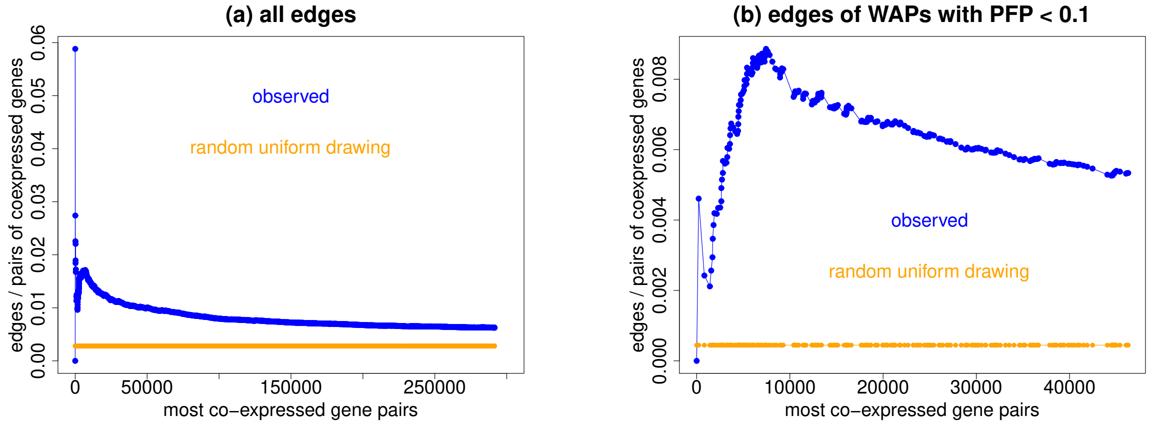

Panel (a) of Figure 14 shows that ordering gene pairs by decreasing covariance (blue) yields higher representation of STRING edges than just choosing random pairs of genes (orange). This representation is however in general below 2%. Note that the maximum value of 6% is actually obtained with the first 17 most co-expressed pairs which overlap with 1 edge of STRING ($1/17 \simeq 0.06$). Overall, one can state that the most co-expressed pairs of genes in GSE58095 have low representation as interactions in STRING (below 2%). In panel (b) of Figure 14, STRING edges are restricted to those involving any of the top 418 WAPs for SSc vs. control in GSE58095 (PFP values less than 0.1). The most co-expressed pairs of genes (blue) have higher representation in the edge set than random pairs of genes (orange). But again, this representation is low: less than 0.9%.

Figure 15: Distributions of overlap statistic maxR over 1,000 random partitions of GSE58095 in two data sets and after removing the top x% edges of STRING ordered by decreasing co-expression in GSE58095. Blue if for WAP scores and green for DEG scores. Vertical bars show 99% bootstrap confidence intervals for median values of maxR.

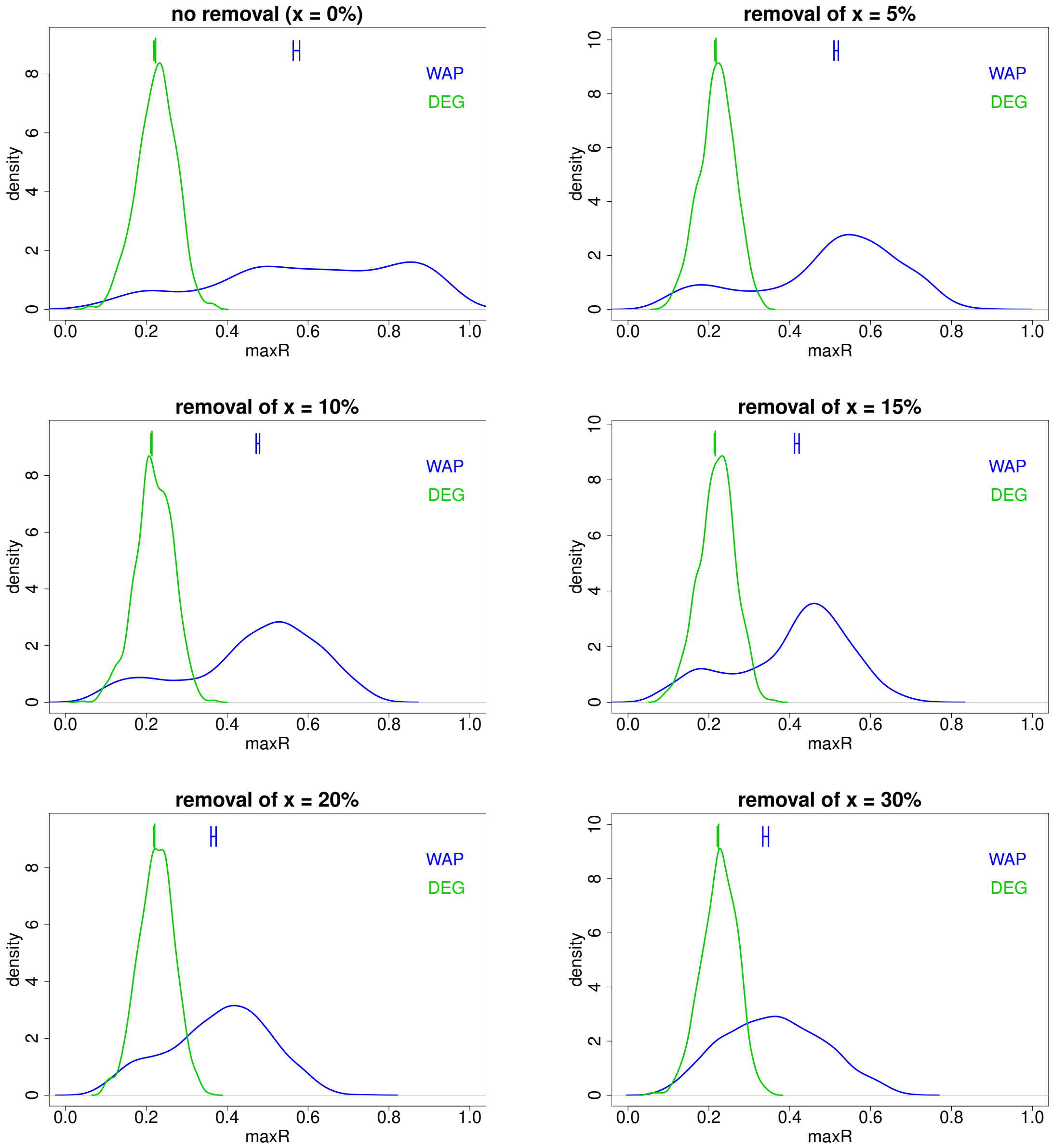

4.3.3 Removal of edges having highly co-expressed genes

In order to test the effect of co-expression in GSE58095 on higher reproducibility of WAP scores versus DEG scores, edges of the protein network are ranked by decreasing co-expression (absolute value of covariance over GSE58095) and the top x% edges are removed. Reproducibility of DEG and WAP scores is assessed by the distributions of overlap statistics over 1,000 random partitions of GSE58095 in two data sets. Results are presented in Figure 15 for statistic maxR and in Figure 16 for statistic AUC. Vertical bars display 99% confidence intervals of median values (maxR or AUC),

Figure 16: Distributions of overlap statistic AUC over 1,000 random partitions of GSE58095 in two data sets and after removing the top $x\%$ edges of STRING ordered by decreasing co-expression in GSE58095. Blue if for WAP scores and green for DEG scores. Vertical bars show 99% bootstrap confidence intervals for median values of AUC.

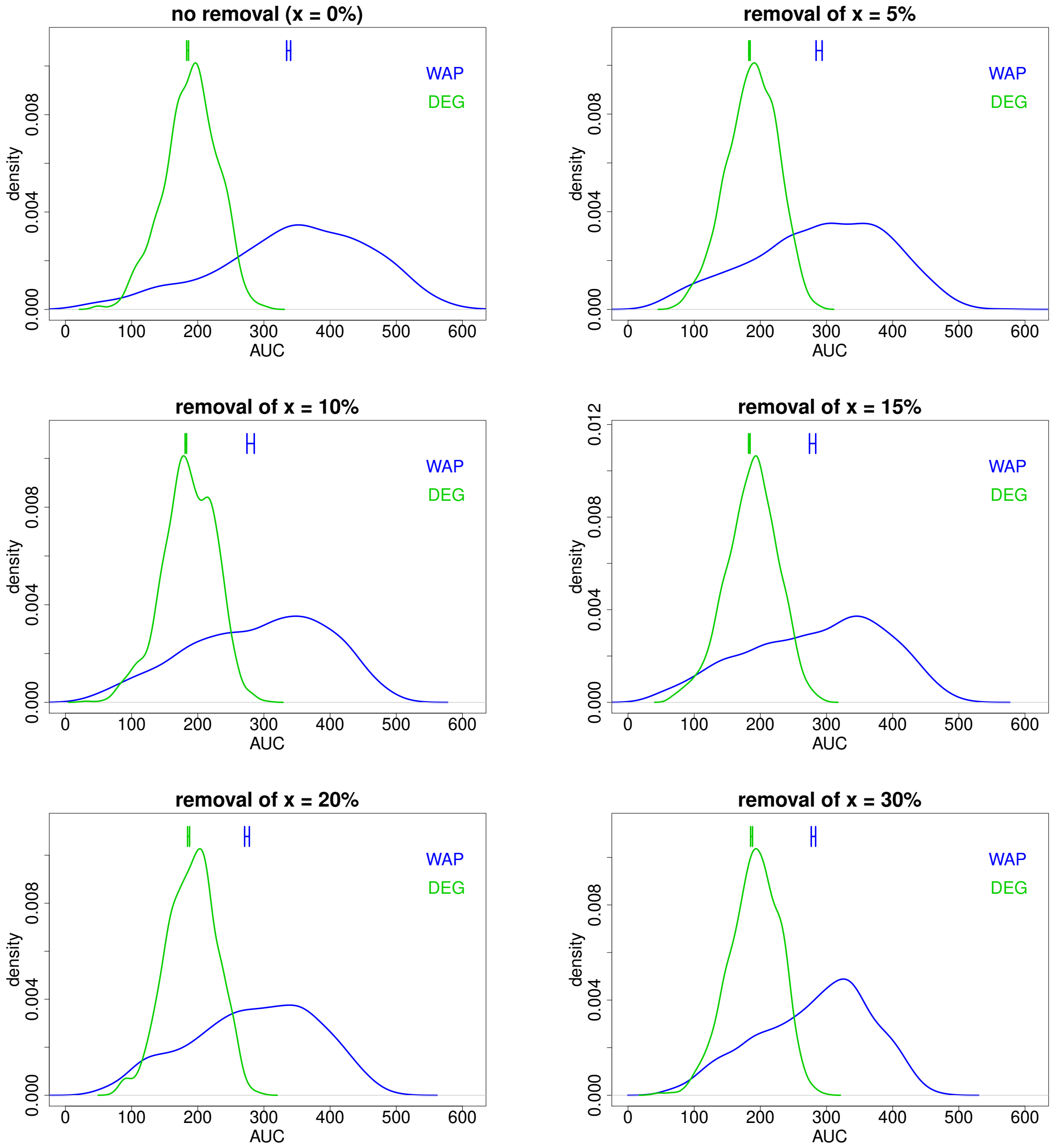

as estimated by bootstrapping with 10^6 samples. One can see that while removing the most co-expressed network edges reduces reproducibility of WAP scores, it still remains on average higher than that of DEG scores, even after removing almost 10^5 edges ($x = 30\%$).

4.3.4 Conclusions

Pairs of genes which are co-expressed across GSE58095 represent only a small fraction (less than 2%) of STRING edges and an even smaller fraction (less than 0.9%) of edges of significant WAPs for SSc

versus control in this data set. It is therefore unlikely that co-expression over GSE58095 explains the observed large proportion of significant WAP scores. Likewise, while removing the most co-expressed pairs of genes from the protein network decreases the average WAP score reproducibility, it still remains higher than that of DEG scores. Robustness of WAP scores is therefore likely to result from the combination of several types of functional relationships within the protein network, co-expression being only one of these.

4.4 Partitions of GSE58095

Reproducibility of DEG and WAP score rankings between random partitions of GSE58095 dataset in two was evaluated for original and rewired STRING network as well as upon random addition or removal of network edges and for the network filtered by edge confidence level. Figures 17 a) and b) display distributions of overlap statistics maxR, left panel a), and AUC, right panel b), for WAP scores (blue), DEG scores (green), WAP scores sorted by their PFP values (pink) and WAP scores with randomly rewired networks (brown), as estimated with 1,000 random partitions of GSE58095 in two data sets. For randomly rewired networks (brown) different styles of the curves represent different extents of rewiring. Solid brown curves (10x) correspond to the WAPs obtained for completely rewired protein interaction networks (that according to Figure 1 show negligible edge overlap with the edges in the original network after $10m$ attempts at rewiring edge pairs, where m is the total number of edges in the network, i.e. the size of the graph). Brown curves formed by the long dashes, short dashes and dots depict reproducibility results for WAPs obtained on less extensively rewired protein interaction networks, that on average preserve 20%, 50% and 80% of the original edges respectively (and corresponding to the numbers of attempted rewiring of edge pairs as indicated in Figure 1 by long dashes, short dashes and dotted lines of varying shades of blue). Such limited amount of rewiring can be seen as an approximation for simultaneous introduction of controlled fraction of false negatives (removed original edges) and false positives (newly formed interactions between randomly chosen nodes in the network) in the protein interaction network.

On average, WAP scores yield better (larger) overlap statistics than DEG scores, and WAP scores with completely rewired networks (10x, solid brown curves) yield low reproducibility, lower than that obtained with DEG scores. On the other hand, WAPs obtained for the networks with moderate amount of rewiring (as an approximation for simultaneous introduction of randomly chosen false negative and false positive edges in protein interaction graph) appear to be fairly resilient to this kind of limited perturbation. When on average 80% of edges are preserved upon rewiring (brown dotted curves) the reproducibility of the resulting WAPs is roughly comparable to that of the WAPs computed on the original network. For the rewiring that preserves on average 50% of the original edges (that can be seen as replacing half of the randomly chosen edges in the network with same number of randomly chosen false positive connections) – brown curves formed by the short dashes – the reproducibility of the resulting WAPs is qualitatively better than that of the DEGs and worse than that of WAPs calculated on the original network. And once only 20% of the original edges remain intact upon rewiring (long brown dashes) the reproducibility becomes approximately comparable to or slightly worse than that of DEGs and marginally better than that of the WAPs obtained on fully rewired network. Lastly, ordering WAPs by their PFP values in the examples shown in Figure 17 panels a) and b) (pink curves) virtually did not impact their reproducibility as quantified by AUC (right panel) and moderately decreased it when quantified by maxR (left panel).

While partial rewiring of pathway network can be viewed as adding controlled amount of noise to the network by simultaneously introducing approximately the same amount of false negatives (by breaking existing pairs of edges selected at random) and false positives (by reconnecting resulting

Figure 17: Distributions of maxR (left) and AUC (right) overlap statistics over 1,000 random partitions of GSE58095 in two data sets. (a)-(b): reproducibility of DEG and WAP score rankings for original and rewired STRING networks. (c)-(d): medians and 99% confidence intervals of overlap statistics for STRING network with randomly added (FP) and removed (FN) edges. (e)-(f): medians and 99% confidence intervals of overlap statistics for STRING network filtered by edge confidence level, θ. See text for further details.

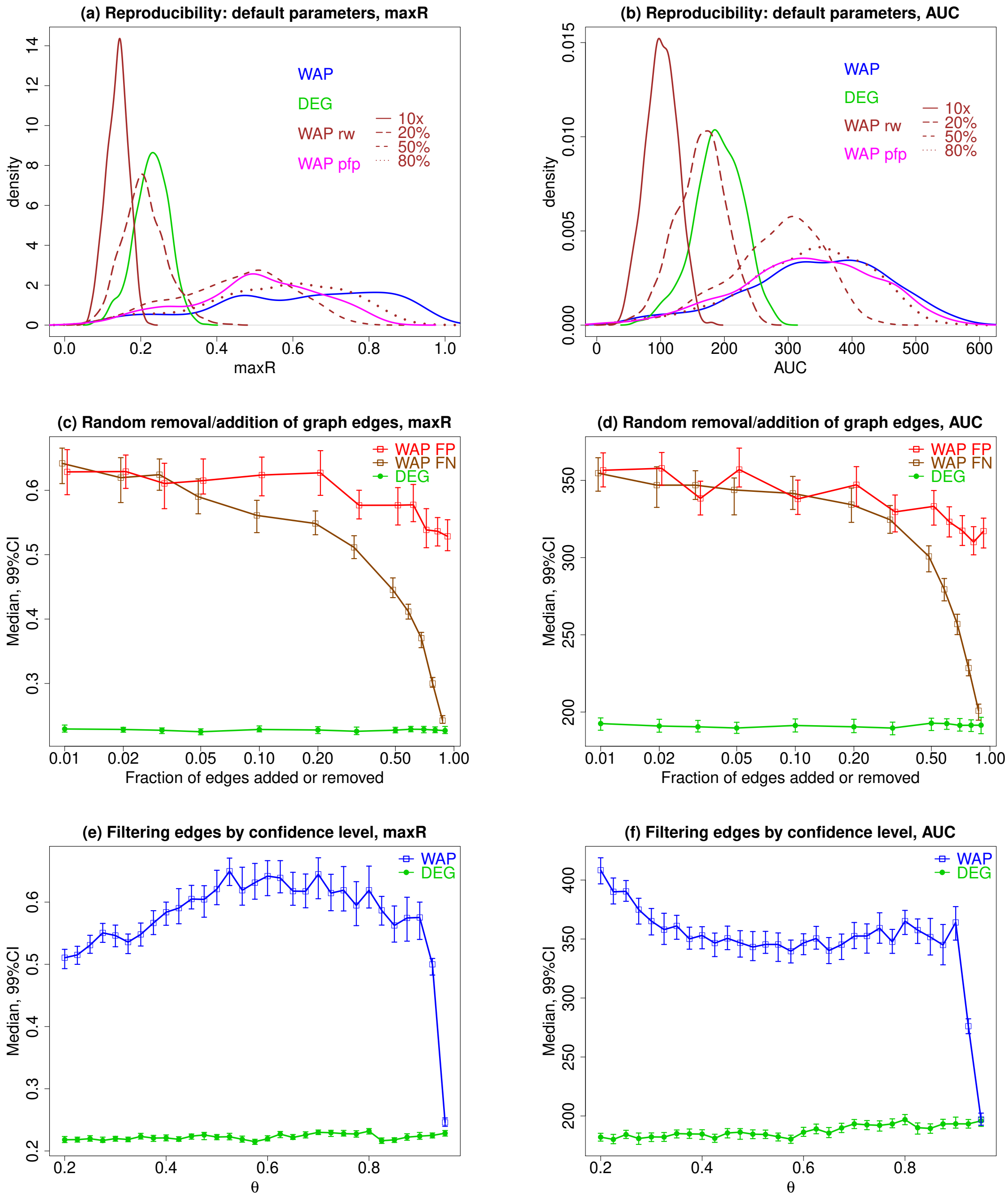

stubs to form new edges not existing in the network yet), it is also instructive to compare reproducibility of the WAP scores' rankings when only false positives (new edges between randomly

chosen vertices) or only false negatives (breaking randomly selected edges) are introduced. Panels c) and d) in Figure 17 summarize these results for 1,000 random partitions of GSE58095 into two sets of samples as described above. For every partition of this dataset in two, random sets of edges were added to or removed from the STRING network and the result was used to obtain WAP scores and rank network vertices for each of the two subsets of the data. Reproducibility of the resulting two lists of genes ranked by their WAP scores has been assessed by maxR, Figure 17 c), and AUC, Figure 17 d). Horizontal axis represents fraction of edges randomly added to or removed from the STRING network. Overlap between gene lists sorted by their DEG scores are shown for reference (green). Consistently with the results presented above in Figure 17, panels a) and b), they display lower overlap than when scored by WAP and, as expected, do not change with the fraction of the edges added to or removed from the network.

Interestingly, the reproducibility of the WAPs trends differently with the fraction of edges added at random (false positives, FP, red in Figure 17 c) and d)) from how it changes with the fraction of edges randomly removed from the network (false negatives, FN, brown in Figure 17 c) and d)). Although median (over 1,000 permutations, error bars represent 99% confidence intervals estimated by 10^4 bootstraps) values of both maxR and AUC decrease with the increase of noise in the network both when false positives and false negatives are introduced to the network, such decrease is far more profound for the increase in the fraction of false negatives (randomly removed edges) as compared to that due to false positives (randomly added edges). This suggests that reproducibility of WAP observations is less sensitive to the amount of false positives than to the amount of false negatives introduced to the network and is consistent with the results reported in the Section 6 below where survey of 23 pathway networks provided by [17] shows on average greater reproducibility of WAP findings for the larger networks.

Random partitions of GSE58095 were further used to assess the impact of edge confidence level, θ on the reproducibility of the rankings of WAP scores. Figures 17 e) and f) represents (blue color) median and 99% CI of maxR and AUC respectively for the overlap between WAP findings in 1,000 random partitions of GSE58095 as function of confidence threshold θ used to subset edges in STRING network. For reference, the reproducibility of DEG scores as quantified by maxR and AUC for random partitions of GSE58095 is shown in Figures 17 e) and f) as well (green). Values of maxR achieve broad maximum for values of $0.5 \lesssim \theta \lesssim 0.7$ while AUC values gradually increase with the decrease in θ (and increase in the resulting network size) at the lower end of the range of the values of θ. For both maxR and AUC the differences between their highest and lowest values across the range of edge confidence scores evaluated here (except for the high end of θ resulting in markedly sparse networks as explained above in Section 4.2) are small comparing to the difference between their values for WAPs and DEGs. Depending on the metric selected for quantifying reproducibility of the WAP score rankings, such evaluation may suggest either thresholding edge confidence score at the level corresponding to the maximum of maxR (e.g. $\theta \approx 0.6$) or using the entire set of edges in the network regardless of their confidence score as maximizing AUC for random partitions of the data. Such an assessment might be useful to conduct when presented with a new dataset from a transcriptional profiling study and/or a new compendium of PPI data that includes quantitative measure of edge confidence/reliability/etc.

5 Cancer, endometriosis and psoriasis data sets

Figures 18, 19, 20, 21, 22, and 23 display distributions of overlap statistics maxR and AUC over 1,000 random partitions of each data set in two. WAP scores for disease versus control (blue) are on average more reproducible than DEG scores (green) and than WAP scores obtained with randomly

rewired networks (brown). Brief descriptions of the eighteen data sets are as follows.

1. GSE41258 [35]: colon cancer, 53 normal samples and 55 tumors
2. GSE44076 [9]: colon cancer, 97 normal samples and 97 tumors
3. GSE44861 [33]: colorectal cancer, 50 normal samples and 48 tumors. Notice that, for this data set WAP scores with the actual protein network yield reproducibility similar to that of WAP scores with randomly rewired networks. Reproducibility of DEG scores is also very low (maxR $\simeq 0.1$)
4. GSE51981 [40]: 72 endometriosis samples and 33 control samples
5. GSE13195 [43]: gastric cancer, 25 normal samples and 25 tumors
6. GSE19826 [42]: gastric cancer, 12 normal samples and 12 tumors
7. GSE27342 [11]: gastric cancer, 75 normal samples and 75 tumors
8. GSE30727 [14]: gastric cancer, 30 normal samples and 30 tumors
9. GSE63089 [44]: gastric cancer, 45 tumors and 43 normal samples
10. GSE79973 [34]: gastric cancer, 10 normal samples and 10 tumors
11. GSE36376 [21]: hepatocellular carcinoma, 193 normal liver samples and 240 tumors
12. GSE19188 [16]: non-small-cell lung carcinoma, 15 normal samples and 17 tumors
13. GSE43458 [19]: lung adenocarcinoma, 38 tumors and 29 normal samples
14. GSE30784 [6]: oral squamous cell carcinoma, 165 tumors and 35 control samples
15. GSE13355 [38]: psoriasis, skin samples, 120 no-lesion samples and 58 lesions
16. GSE30999 [37]: psoriasis, skin samples, 81 no-lesion samples and 83 lesions
17. GSE34248 [4]: psoriasis, skin samples, 14 no-lesion samples and 14 lesions
18. GSE41662 [5]: psoriasis, skin samples, 22 no-lesion samples and 24 lesions

Figure 18: Distributions of maxR (left) and AUC (right) overlap statistics over 1,000 random partitions of a data set in two. Green is for DEG scores, blue for WAP scores, brown for WAP scores with randomly rewired protein networks and pink for WAP PFP values. Vertical lines display 99% confidence intervals (bootstrap) of median values. Dashes represent results for protein network randomly rewired to preserve on average approximately 50% of the original edges.

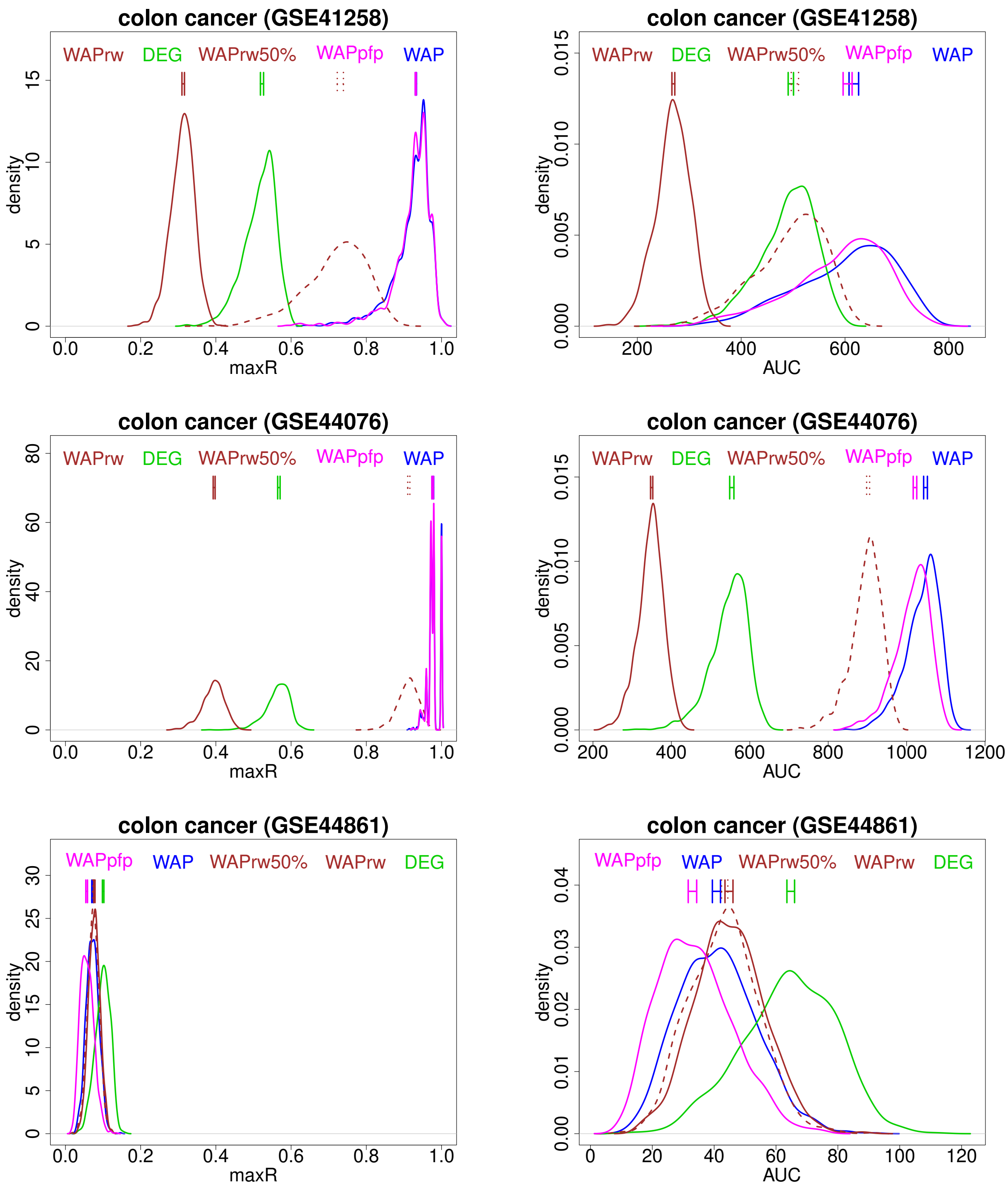

Figure 19: Distributions of maxR (left) and AUC (right) overlap statistics over 1,000 random partitions of a data set in two. Green is for DEG scores, blue for WAP scores, brown for WAP scores with randomly rewired protein networks and pink for WAP PFP values. Vertical lines display 99% confidence intervals (bootstrap) of median values. Dashes represent results for protein network randomly rewired to preserve on average approximately 50% of the original edges.

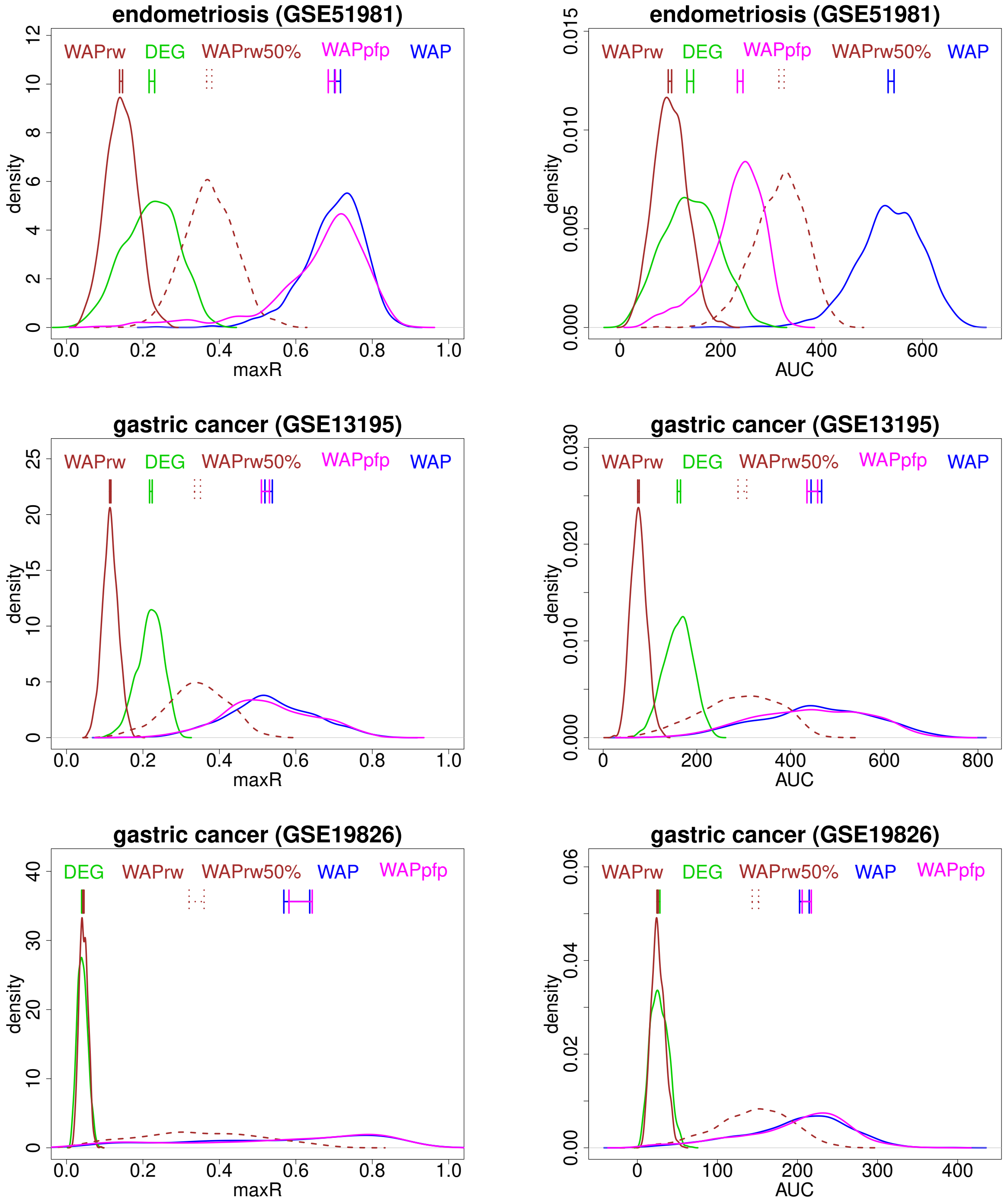

Figure 20: Distributions of maxR (left) and AUC (right) overlap statistics over 1,000 random partitions of a data set in two. Green is for DEG scores, blue for WAP scores, brown for WAP scores with randomly rewired protein networks and pink for WAP PFP values. Vertical lines display 99% confidence intervals (bootstrap) of median values. Dashes represent results for protein network randomly rewired to preserve on average approximately 50% of the original edges.

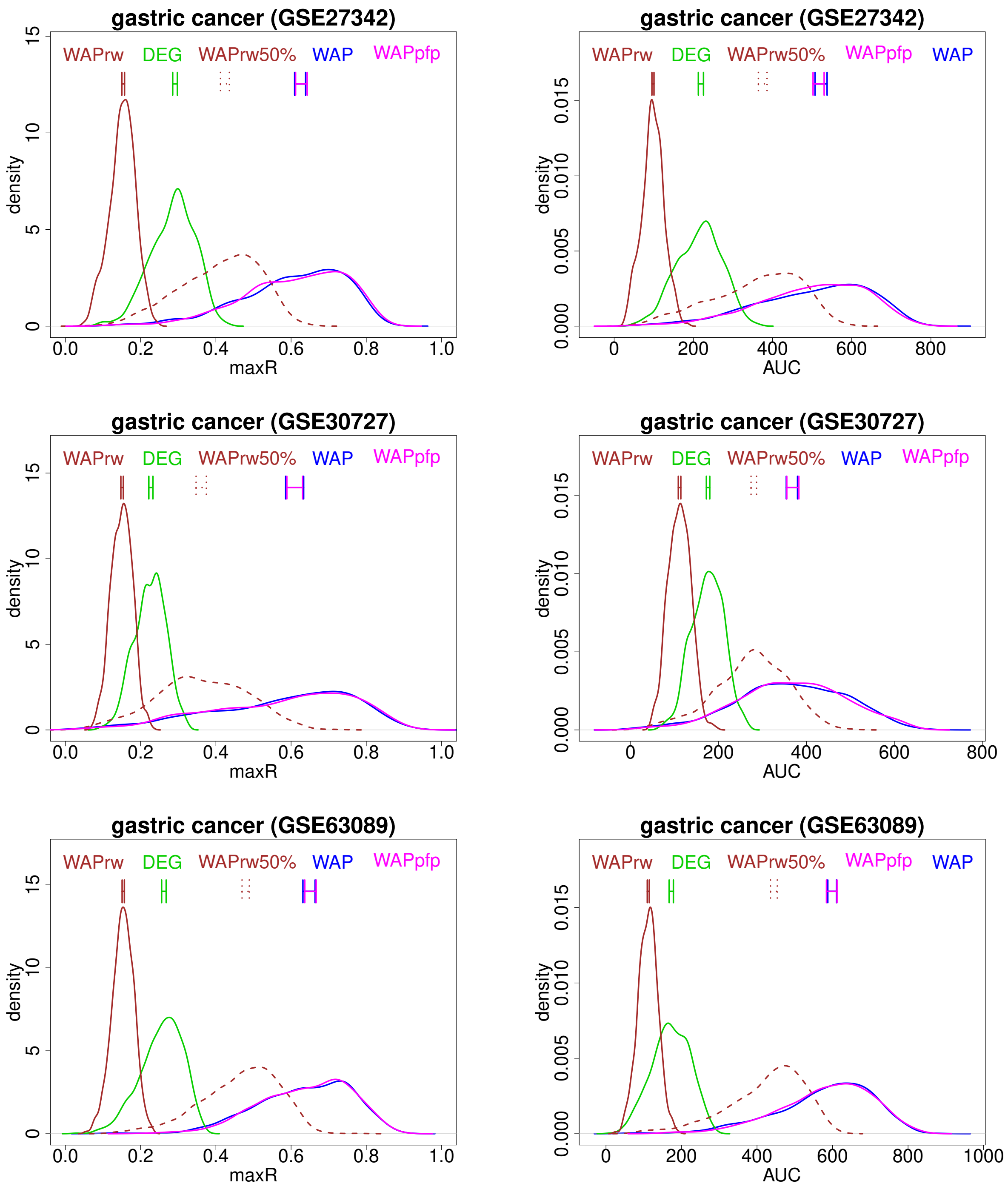

Figure 21: Distributions of maxR (left) and AUC (right) overlap statistics over 1,000 random partitions of a data set in two. Green is for DEG scores, blue for WAP scores, brown for WAP scores with randomly rewired protein networks and pink for WAP PFP values. Vertical lines display 99% confidence intervals (bootstrap) of median values. Dashes represent results for protein network randomly rewired to preserve on average approximately 50% of the original edges.

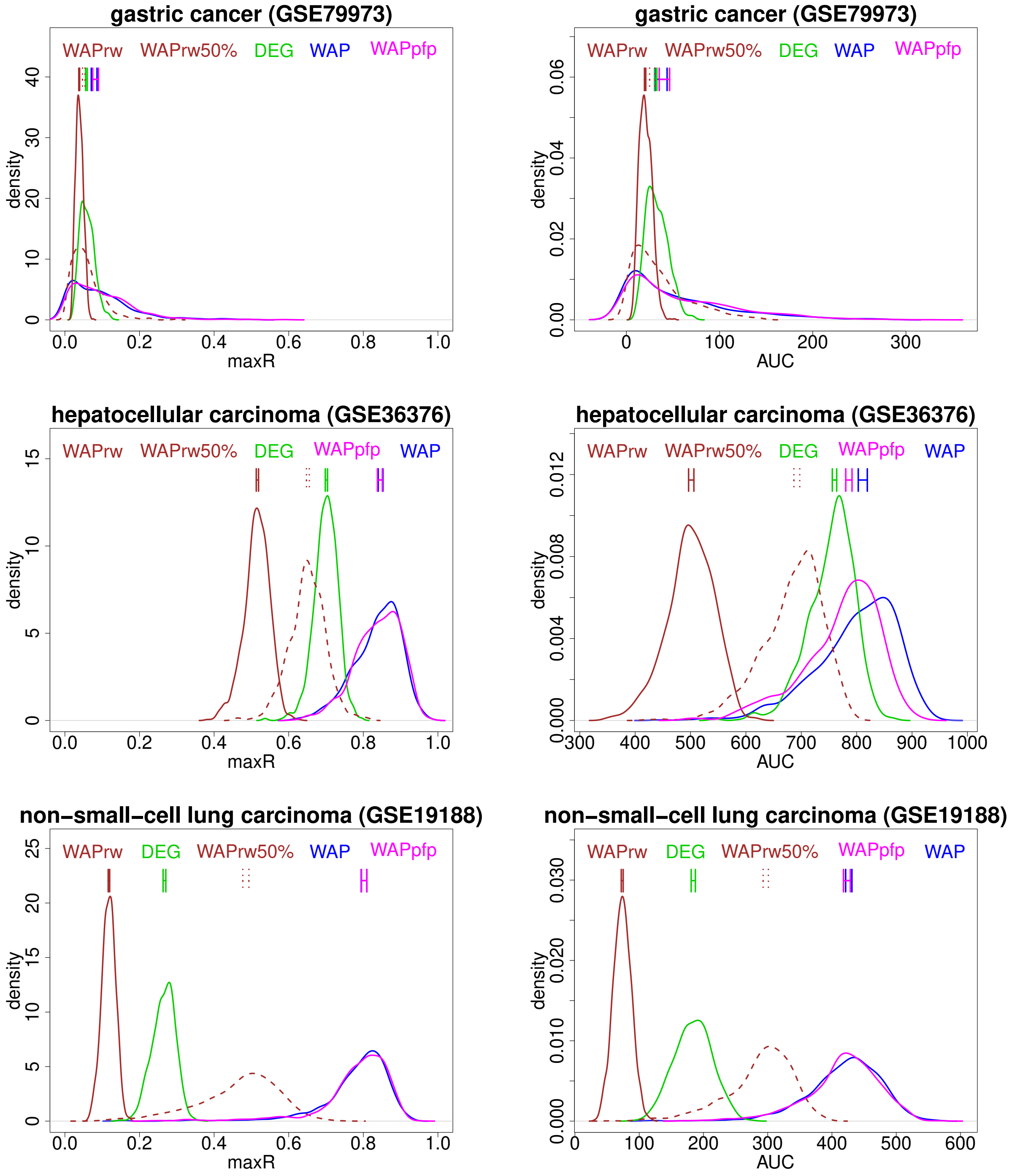

Figure 22: Distributions of maxR (left) and AUC (right) overlap statistics over 1,000 random partitions of a data set in two. Green is for DEG scores, blue for WAP scores, brown for WAP scores with randomly rewired protein networks and pink for WAP PFP values. Vertical lines display 99% confidence intervals (bootstrap) of median values. Dashes represent results for protein network randomly rewired to preserve on average approximately 50% of the original edges.

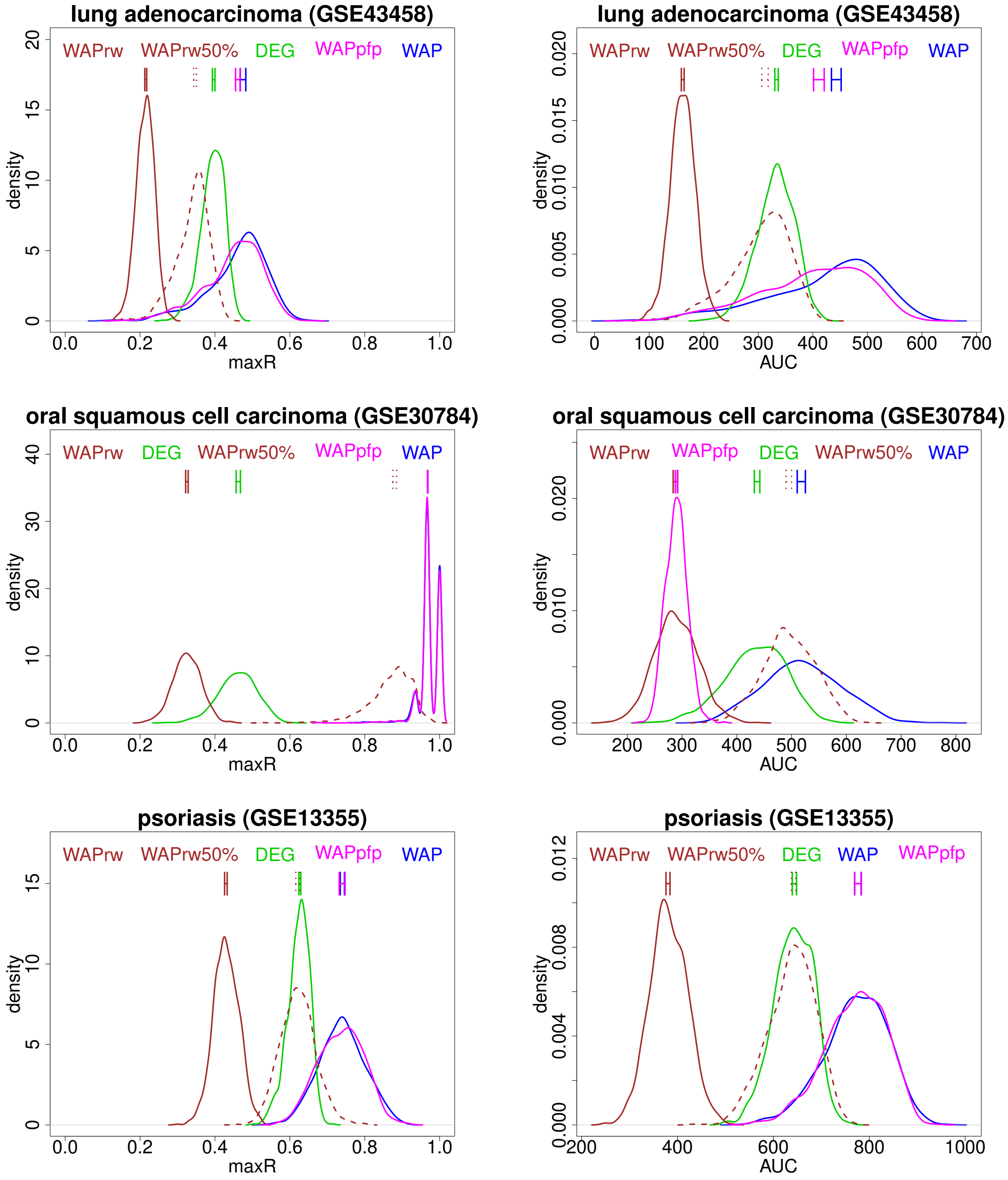

Figure 23: Distributions of maxR (left) and AUC (right) overlap statistics over 1,000 random partitions of a data set in two. Green is for DEG scores, blue for WAP scores, brown for WAP scores with randomly rewired protein networks and pink for WAP PFP values. Vertical lines display 99% confidence intervals (bootstrap) of median values. Dashes represent results for protein network randomly rewired to preserve on average approximately 50% of the original edges.

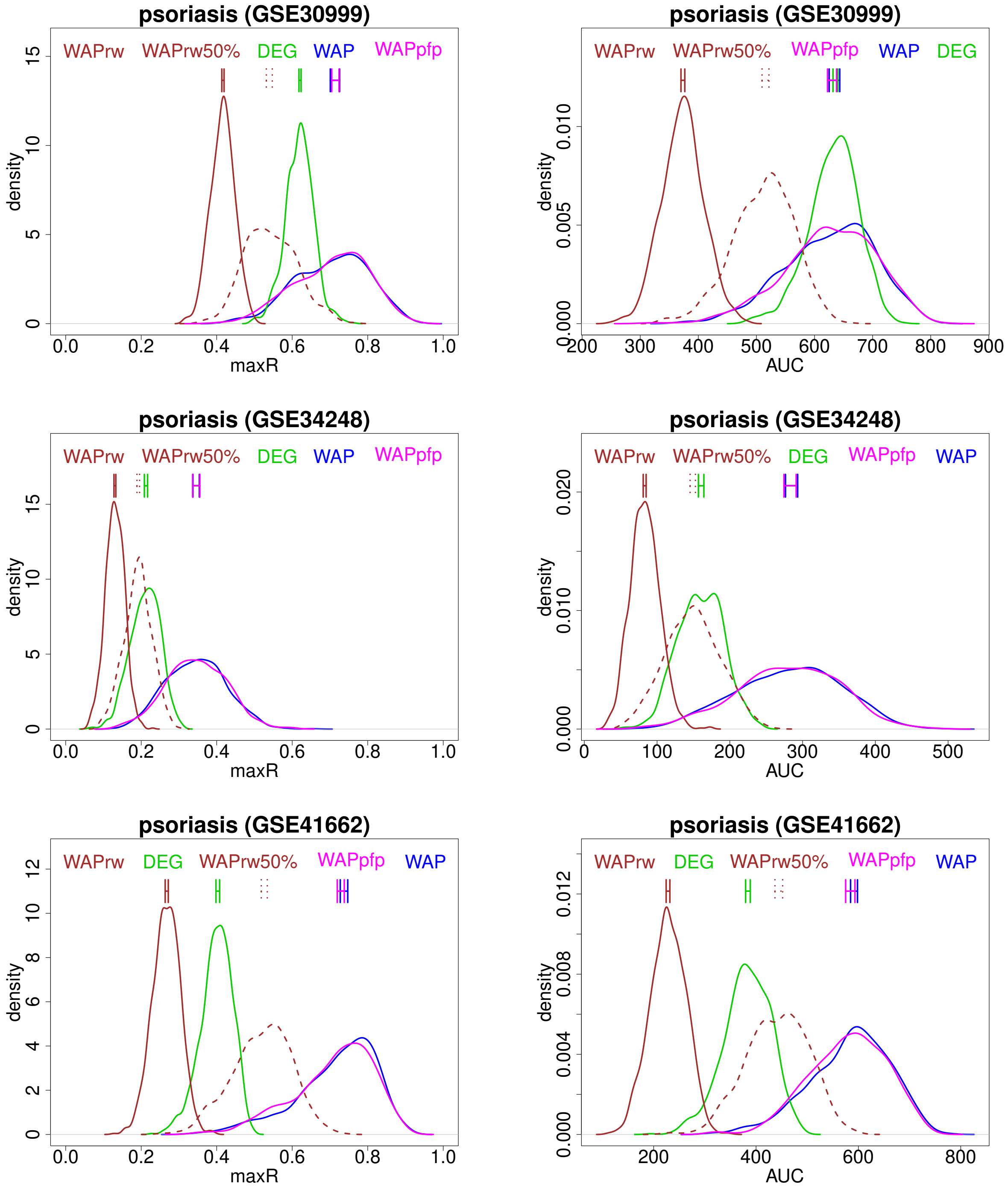

Figure 24: Summary of distributions of overlap statistics maxR (top) and AUC (bottom) differences between WAP and DEG scores over 1,000 random partitions and for 18 data sets. Paired values (WAP - DEG) over each partition were utilized to estimate distributions.

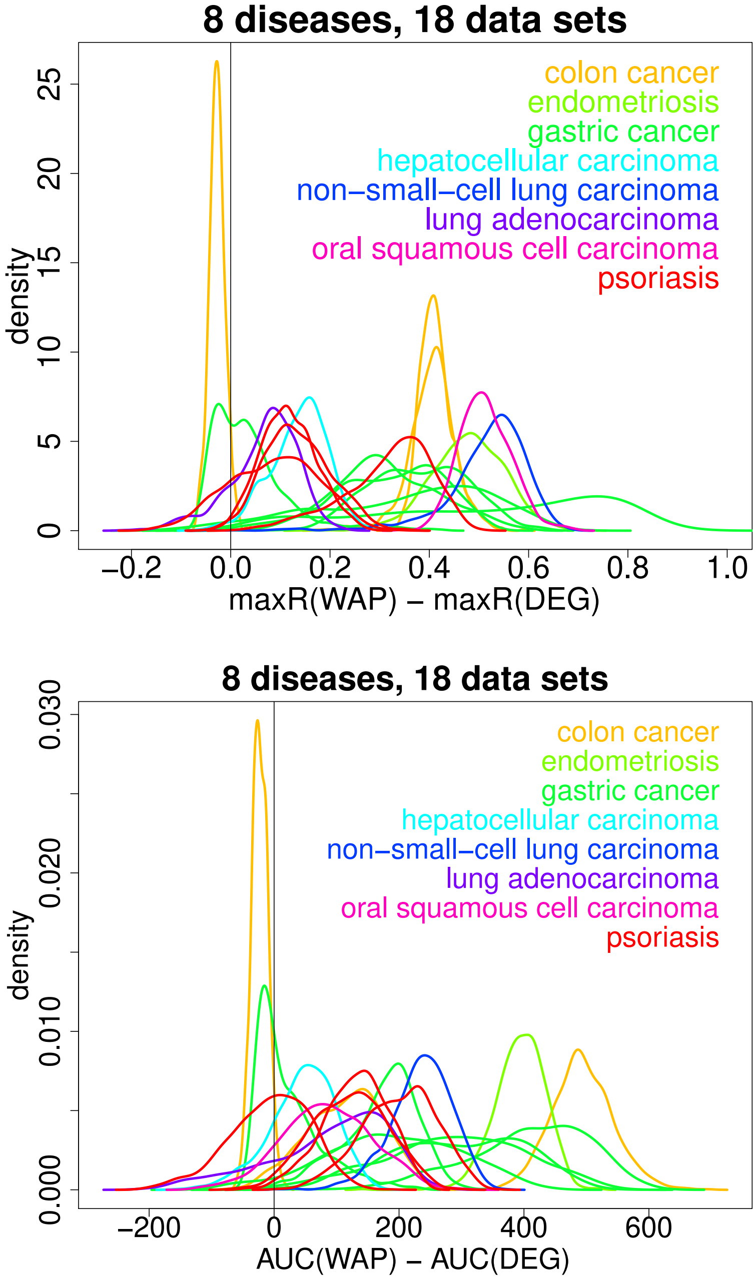

6 Reproducibility of WAP findings across pathway networks

Recent publication by Huang et al. [17] made several datasets representing PPI readily available through NDEx [30, 26, 29] that were used to evaluate reproducibility of the WAP findings across wide variety of PPI network sizes and contents. Specifically, from the NCBI-GEO datasets described above, the following have been selected as multiple representations of the same disease vs. healthy comparison:

- colon cancer: GSE41258 and GSE44076
- gastric cancer: GSE13195, GSE19826, GSE27342, GSE30727, GSE63089 and GSE79973
- psoriasis: GSE13355, GSE30999, GSE34248 and GSE41662
- systemic sclerosis: GSE58095, GSE32413, GSE9285 and GSE45485

For each of these four sets of NCBI-GEO datasets, genes were ranked by their DEG and WAP scores (prior to the correction for the degree bias) for each of the networks deposited to the NDEx by Huang et al. (as listed under "Deposited Data" in "Key Resources Table" in [17]). Then, for each of these four diseases, for each pair of the datasets representing the same disease the reproducibility of the gene ranking by their DEG and WAP scores was compared for the subset of the genes present in both datasets and on a given network using maxR and AUC metrics.

Results of this assessment are summarized in Figure 25 as average difference between maxR for DEGs and WAPs: $maxR\,(WAP) - maxR\,(DEG)$ (and similarly, $AUC\,(WAP) - AUC\,(DEG)$) for each of the four diseases evaluated herein per each PPI network analyzed. To account for a wide range of the number of edges spanned by these networks: a) the values of maxR and AUC were calculated over top 5% of the most highly scoring WAPs and DEGs in each case, and b) in case of AUC, the overlap statistic was normalized to the number of genes involved in calculation so that it assumes values within [0,1] interval. Horizontal dashes in the figure correspond to no difference in maxR (or AUC) values between WAP and DEG score rankings.

Overall, across 16 datasets, four diseases and 23 PPI networks evaluated in this assessment reproducibility of gene rankings by WAP scores tends to be higher than by DEG scores with the values plotted spanning greater range of positive as compared to negative values. This tendency is more pronounced for the networks with the larger number of edges. All but one average values of $maxR\,(WAP) - maxR\,(DEG)$ and $AUC\,(WAP) - AUC\,(DEG)$ that are below zero (therefore indicating higher reproducibility of DEG rankings than WAPs) have been observed for the networks with $\lesssim 3 \times 10^5$ edges. This suggests that higher volume of information encoded in PPI networks tends to yield more reproducible findings by WAP methodology. This observation is consistent with: a) the results reported above in Section 4.4 regarding greater impact of false negatives on the reproducibility of WAP observations, and b) the findings in [17] concluding that "general tendency is that performance scales with network size, suggesting that new interaction discovery currently outweighs the detrimental effects of false positives".

Figure 25: Average and standard deviation of the WAP-DEG difference for overlap statistics (AUC – top panel, maxR – bottom panel) among four diseases represented by multiple NCBI-GEO datasets evaluated in this study: colon cancer (Col), gastric cancer (Gas), psoriasis (Pso) and systemic sclerosis (SSc) across pathway networks evaluated and generated (PCNet and FCNet) in [17]. Positive values on the y-axis correspond to higher reproducibility of WAP score rankings between different datasets characterizing the same disease. The results for individual diseases are plotted with a horizontal offset within each network size to decrease overplotting. See text for further details.

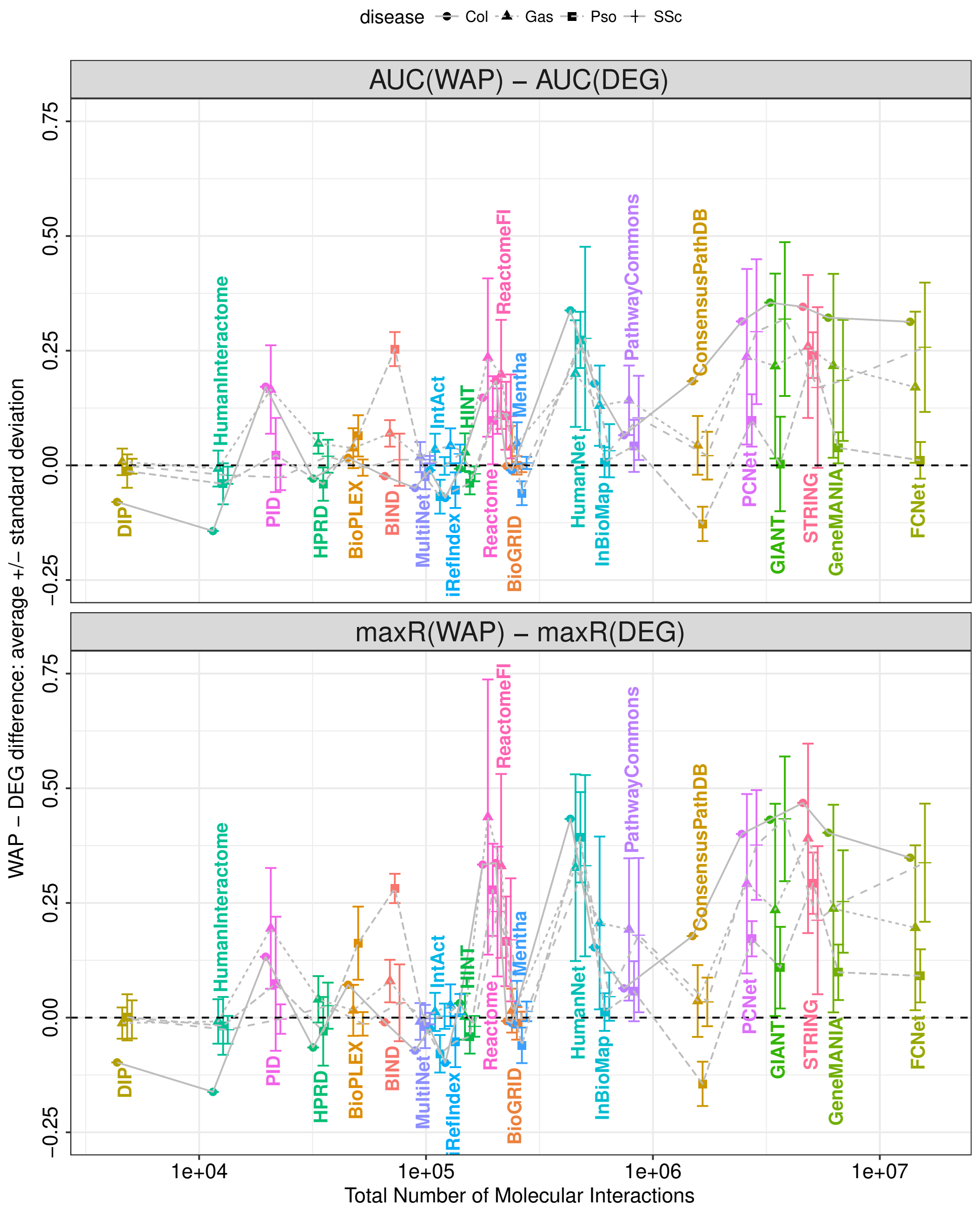

7 Ranking of WAPs and DEGs by differential expression prior

Recent publication by Crow and colleagues [10] has introduced ranking of genes on Affymetrix Human Genome U133 Plus 2.0 Array by their frequency of passing cutoffs for differential expression (BH-FDR, < 0.05; absolute $\log_2$ fold change, > 2) across large compendium of uniformly analyzed gene expression data (> 600 datasets, > 27K samples) that spanned wide variety of biological phenomena. Resulting empirical estimate of the prior probability of differential expression (DE) of individual genes (termed "DE prior" by Crow and colleagues) was highly predictive of genes passing above significance cutoffs in leave-one-out benchmarking performed in [10] yielding mean area under the receiver operating characteristic curve (AUROC) of 0.83 ± 0.1.

In order to evaluate the capacity of DE prior for identifying significant WAPs, DE prior AUROC values have been calculated for the significant WAPs (PFP < 0.05) and compared to the corresponding DE prior AUROC values for the significant DEGs (BH-FDR < 0.05; absolute $\log_2$ fold change > 2). Figure 26 represents graphically the results of this comparison across 22 NCBI-GEO datasets evaluated in this study. Individual gene ranking by DE prior was provided by the supplementary Dataset S2 from [10]. PFP values for WAPs have been calculated as described in the subsection "The concept of well-associated protein" of the "Materials and methods" in the main text. BH-FDR values and log base 2 fold-change for individual genes have been calculated using R/Bioconductor package "limma" [32]. For each dataset only the overlap between genes included in DE prior ranking and those present in this dataset was considered. The calculation of DE prior AUROC values for the significant WAPs or DEGs was performed by R library "ROCR".

Figure 26: DE prior AUROC values for the significant WAPs and DEGs across NCBI-GEO datasets evaluated in this study. Diagonal dashes represent identity $y = x$ line. Three NCBI-GEO datasets (GSE9285, GSE44861 and GSE58095) for which no significant DEGs have been detected are omitted from display. See text for further details.

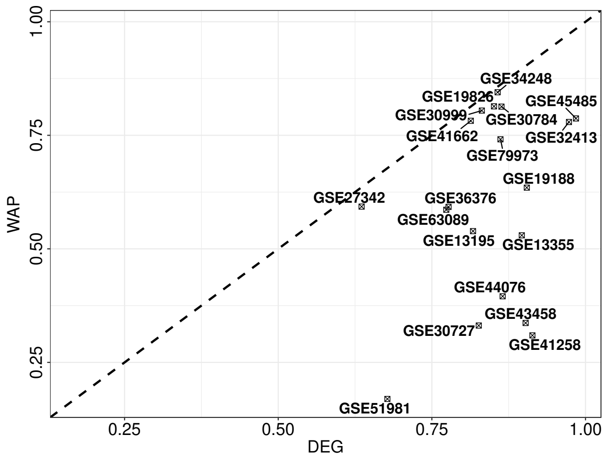

For the 19 out of 22 NCBI-GEO datasets evaluated herein that had greater than zero number of significant DEGs (passing the cutoffs specified above) average prior DE AUROC of DEGs was AUROC(DEG) $= 0.84 \pm 0.02$ consistently with a similarly high value cited above as reported by [10]. This suggests generalizability of DE prior as calculated in [10] for predicting differential gene expression for platforms other than HGU-133 Plus 2.0, as over half of the datasets evaluated here have been characterized using transcriptional profiling approaches other than HGU-133 Plus 2.0 technology.

Values of DE prior AUROC for the significant WAPs for each of these 19 datasets were invariably lower than those for the significant DEGs in the same dataset (average AUROC(WAP) = 0.60±0.05; median AUROC(DEG) − AUROC(WAP) = 0.19, IQR = 0.37). Systematically lower values of DE prior AUROC for the significant WAPs suggest that DE prior is less predictive of the significance of WAPs as compared to its predictive power for the significance of DEGs.

Three remaining NCBI-GEO datasets (GSE9285, GSE44861 and GSE58095) were omitted from Figure 26 as none of the genes in these datasets passed the threshold of log base 2 fold-change > 2 used as described above for the identification of the significant DEGs. Corresponding DE prior AUROC values for the significant WAPs in these three datasets are 0.53, 0.55 and 0.67 respectively, also indicating low information content of DE prior for the detection of the significant WAPs in these three datasets where no significant DEGs have been identified.

Summarily, these results imply that the increased reproducibility of WAPs as compared to that of DEGs for the datasets evaluated herein does not amount to enriching WAPs for the genes ranked highly by DE prior. Therefore, this plausibly reflects relevance of pathway knowledge for reproducibly detecting network neighborhoods significantly perturbed in transcriptional profiling studies comparing disease tissue to healthy controls. Such decrease in predictability of significant WAPs by the DE prior ranking (as compared to its capacity for identifying significant DEGs) suggests the potential of WAPs to yield not only findings that are more reproducible (for within-dataset and within-disease comparisons as illustrated above), but that such WAPs could be also more specific for a given disease than DEGs. More systematic assessment of this possibility across suitably broad collection of transcriptional datasets represents another intriguing opportunity for further research.

8 Necessary conditions for WAP score robustness

8.1 Sampling of perturbed data set pairs

Figure 27: Distance to a uniform distribution over 2×500 points for average PFP values (over the top 200 WAPs) and MVT p-values as a function of perturbation parameter ρ.

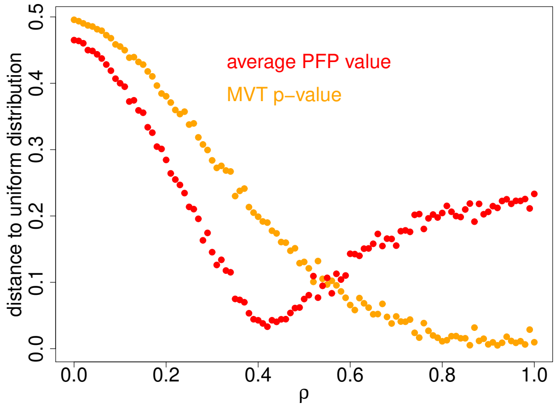

Data set GSE58095 is randomly partitioned in two data sets, each one having half of the SSc samples and half of the control samples. Each data set tends to have high signal for SSc versus control: small average PFP value over the top 200 WAPs and small MVT p-value. In order to sample a wider range of average PFP values or MVT p-values, each data set can be randomly perturbed for its SSc/control composition. Disease labels of samples are copied into an array, the index identifying a sample. Then at each index, a label is randomly selected with probability ρ. If chosen, the label is swapped with another one at a uniformly randomly chosen index. Setting $\rho = 0$ does not perturb disease labels and $\rho = 1$ yields maximal perturbation with this scheme. For each value of ρ, 500 pairs of data sets are generated and distance to a uniform distribution in $[0;1]$ is estimated:

$$d(\mathbf{x}) = \frac{1}{1000} \sum_{i=1}^{1000} \left| x^{(i)} - \frac{i}{1001} \right|,$$

where $x^{(i)}$ is the ith smallest value of average PFP or MVT p-value.

Figure 27 shows that, as one expects because the MVT p-value is estimated via permutation testing on SSc/control, uniform sampling of MVT p-values is best achieved by setting $\rho = 1$ (orange dots). For the average PFP value (red dots), close to uniform sampling is achieved by setting $\rho = 0.41$.

A total of 2×10^5 pairs of data sets are generated: 2×10^4 with $\rho = 0$, 9×10^4 with $\rho = 0.41$ for uniform sampling of average PFP values and 9×10^4 with $\rho = 1$ for uniform sampling of MVT p-values. Estimated distributions of overlap statistics maxR and AUC, for DEG and WAP scores are presented in Figures 28, 29 and 30 as functions of characteristics of data set pairs (average PFP values or MVT p-values).

8.2 Overlap statistics as a function of data set pair characteristics

Figure 28: Average overlap statistics (maxR and AUC) of DEG and WAP scores over 2×10^5 partitions of GSE58095 in two data set, and as function of the average PFP value in each data set.

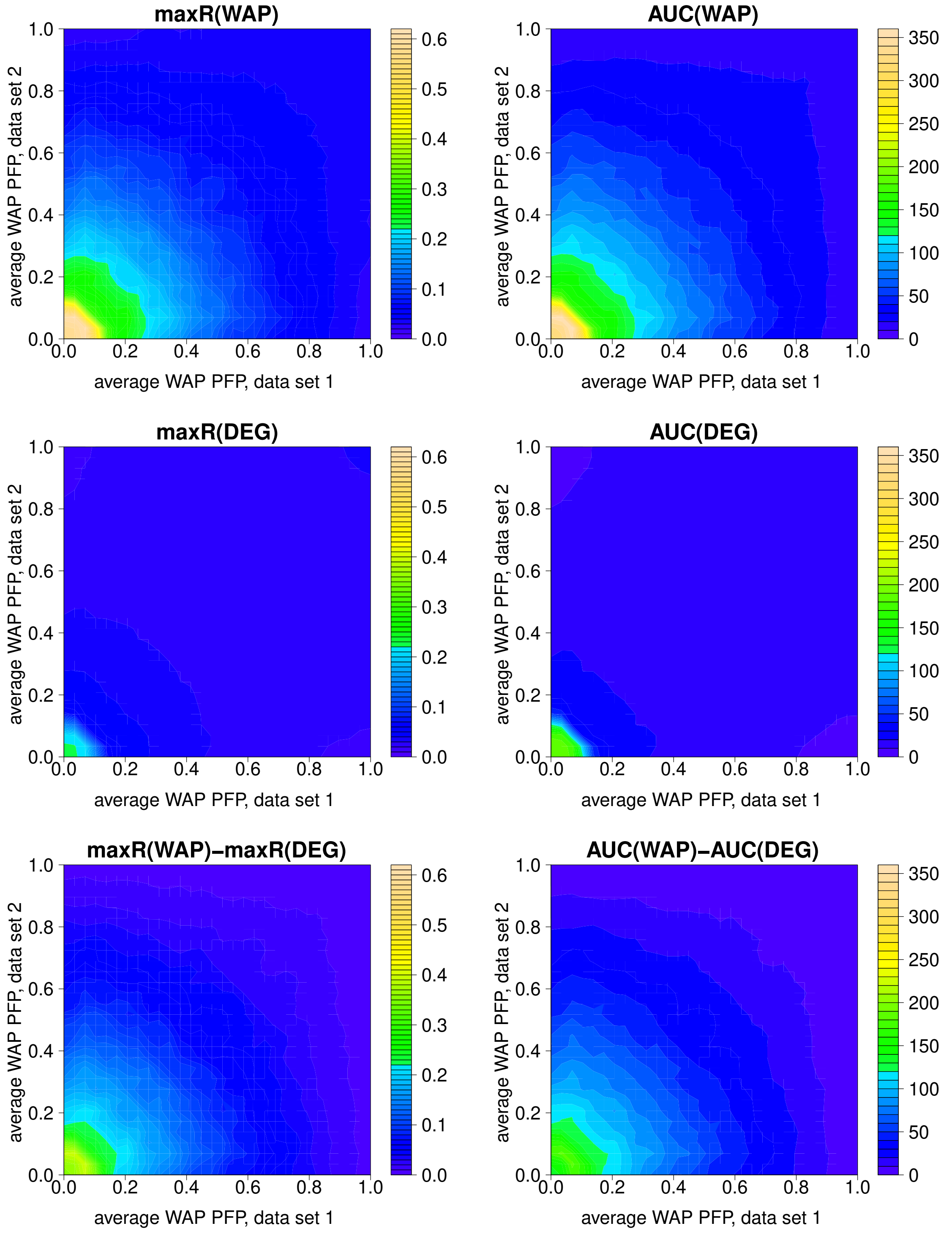

Figure 29: Average overlap statistics (maxR and AUC) of DEG and WAP scores over 2×10^5 partitions of GSE58095 in two data set, and as function of the MVT p-value in each data set.

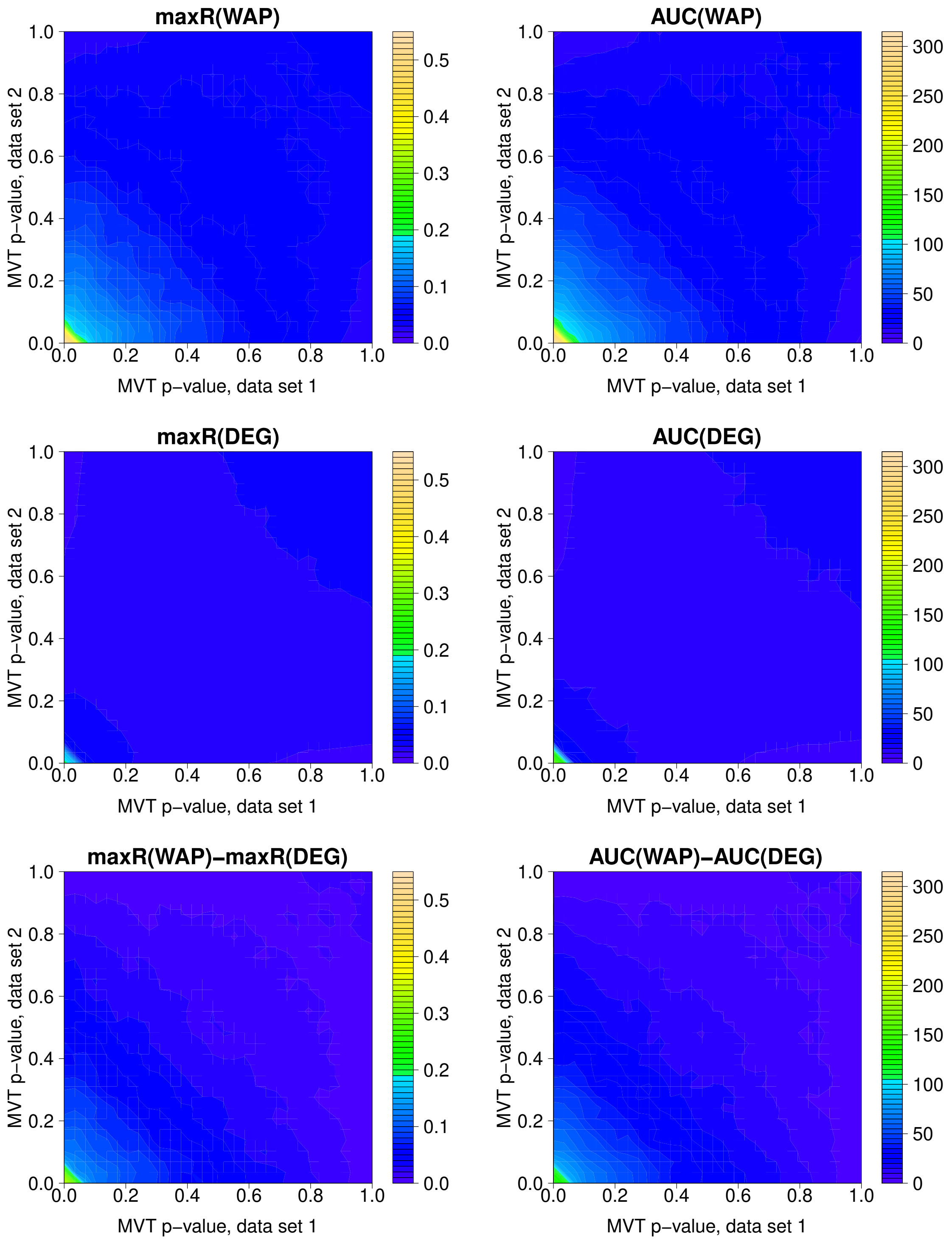

Figure 30: Average overlap statistics (maxR and AUC) of DEG and WAP scores over 2×10^5 partitions of GSE58095 in two data set, and as function of the MVT p-value in one data set and the average PFP value in the other.

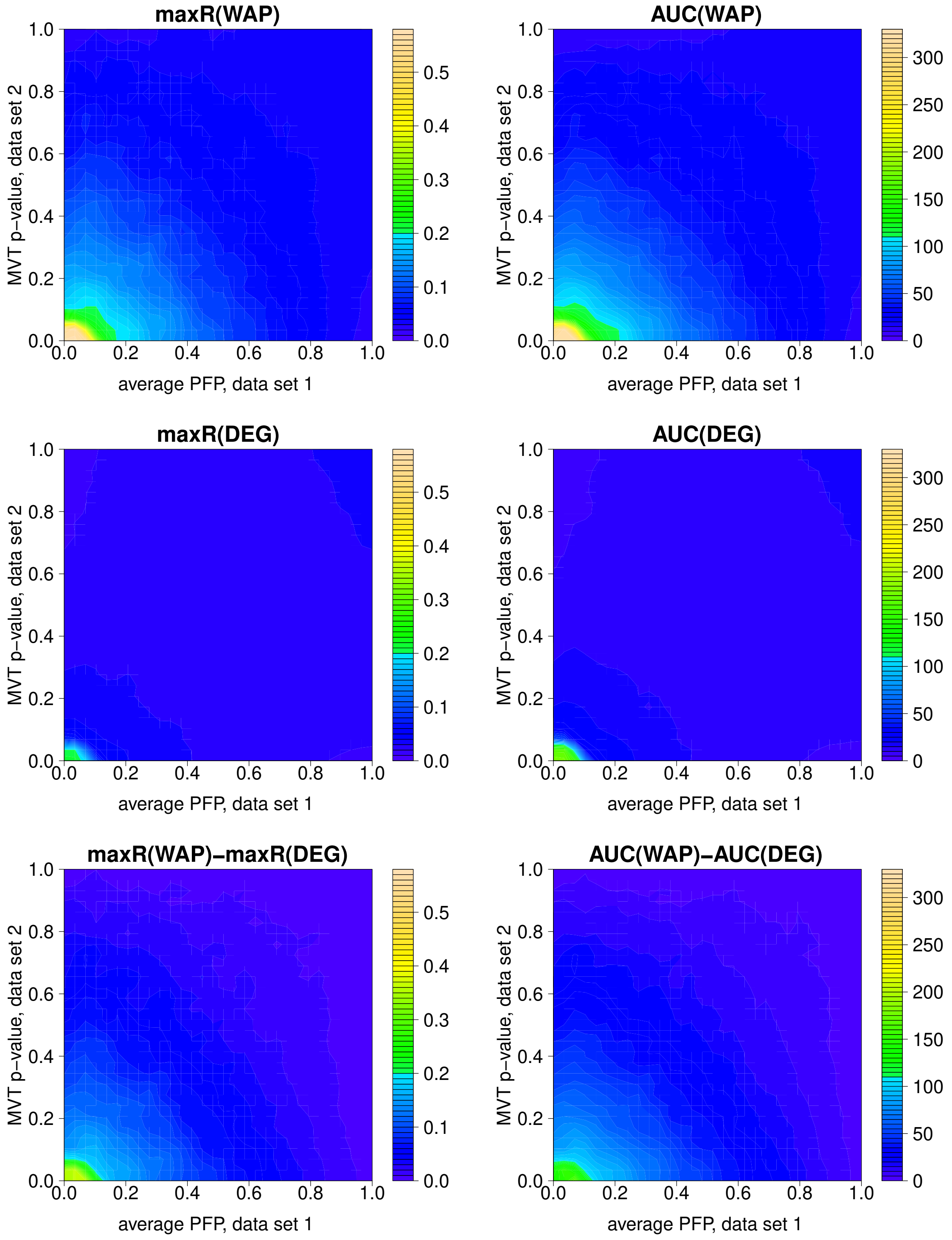

Tables 2, 3 and 4 display summary statistics for matrices presented in Figures 28, 29 and 30. Notice

Table 2: Summary statistics for matrices of Figure 28.

matrix	maximum	average	maximum	average	matrix
maxR WAP	0.611	0.089	360	49	AUC WAP
maxR DEG	0.222	0.030	185	16	AUC DEG
maxR(WAP) - maxR(DEG)	0.400	0.059	183	33	AUC(WAP) - AUC(DEG)

Table 3: Summary statistics for matrices of Figure 29.

matrix	maximum	average	maximum	average	matrix
maxR WAP	0.547	0.055	311	29	AUC WAP
maxR DEG	0.202	0.028	167	14	AUC DEG
maxR(WAP) - maxR(DEG)	0.345	0.027	144	15	AUC(WAP) - AUC(DEG)

that the average maxR or AUC statistics for differences (WAP - DEG) are always, by definition, equal to the difference of individual estimations (WAP or DEG) of average maxR or AUC values. This is not necessarily true for maximum values of maxR(WAP)-maxR(DEG) (or AUC(WAP)-AUC(DEG)) because maximum values of these differential variables do not necessarily coincide with maximum values of the individual ones.

8.3 Correlation between MVT p-value and average gene FDR value

Call d_{ij} the Pearson dissimilarity between two samples i and j, based on all array spots. Values of d_{ij} close to 0 mean that the two samples have similar expression values across all genes, and values close to 1 indicate dissimilarity. Call $\mathcal{D}$ and $\mathcal{C}$ disease and control groups of samples. The Multi-Variate T statistic [12, 36], is defined by

$$\text{mvt}\left(\mathcal{D},\mathcal{C}\right) = \frac{d\left(\mathcal{D},\mathcal{C}\right)}{s\left(\mathcal{D}\right)+s\left(\mathcal{C}\right)} \quad \text{with} \quad d\left(\mathcal{D},\mathcal{C}\right) = \frac{\sum_{i\in\mathcal{D},j\in\mathcal{C}} d_{ij}}{|\mathcal{D}|\,|\mathcal{C}|} \quad \text{and} \quad s\left(\mathcal{D}\right) = \frac{2\sum_{i<j\in\mathcal{D}} d_{ij}}{|\mathcal{D}|\left(|\mathcal{D}|-1\right)}. \tag{11}$$

Statistic, mvt is the ratio of difference between groups (d) to their spread (s). Large values of mvt therefore indicate separation of the two groups. To define what large is, permutation testing is utilized. The observed value (mvt) is compared to values (MVT) obtained when randomly shuffling samples between groups $\mathcal{D}$ and $\mathcal{C}$, and the p-value

$$p = \Pr\left(\text{MVT} \geq \text{mvt}\right) \tag{12}$$

is small compared to 1 if the two groups are markedly different.

Figure 31 shows that MVT p-values are relatively well correlated with average DEG FDR values, FDR values being estimated as in [3]. One thousand (200 now) perturbed data sets were sampled from GSE58095, with perturbation parameter ($\rho = 0.3$) for SSc/control skin samples. For each perturbed data set the MVT p-value was estimated via permutation testing and FDR values (on two-sided t-test p-values) were estimated for all genes as in [3]. Figure 31 displays, in log scale, scatterplots of MVT p-value and average FDR value over the top n smallest ones. Correlation between the two variables tends to increase with n. Applying a two-sided Spearman correlation test yields the following estimates s: $s = 0.934$ (A), $s = 0.949$ (B), $s = 0.958$ (C) and $s = 0.961$ (D). Given the large number of pairs (1000) all estimated p-values are reported as 0.

Table 4: Summary statistics for matrices of Figure 30.

matrix	maximum	average	maximum	average	matrix
maxR WAP	0.574	0.068	327	37	AUC WAP
maxR DEG	0.216	0.028	179	14	AUC DEG
maxR(WAP) - maxR(DEG)	0.364	0.040	170	22	AUC(WAP) - AUC(DEG)

Figure 31: Correlation between MVT p-value and average FDR value over the top n genes. One thousand perturbed data sets ($\rho = 0.3$) were sampled from GSE58095 (SSc skin samples versus control samples). A: $n = 200$. B: $n = 500$. C: $n = 1000$. D: $n = 5000$.

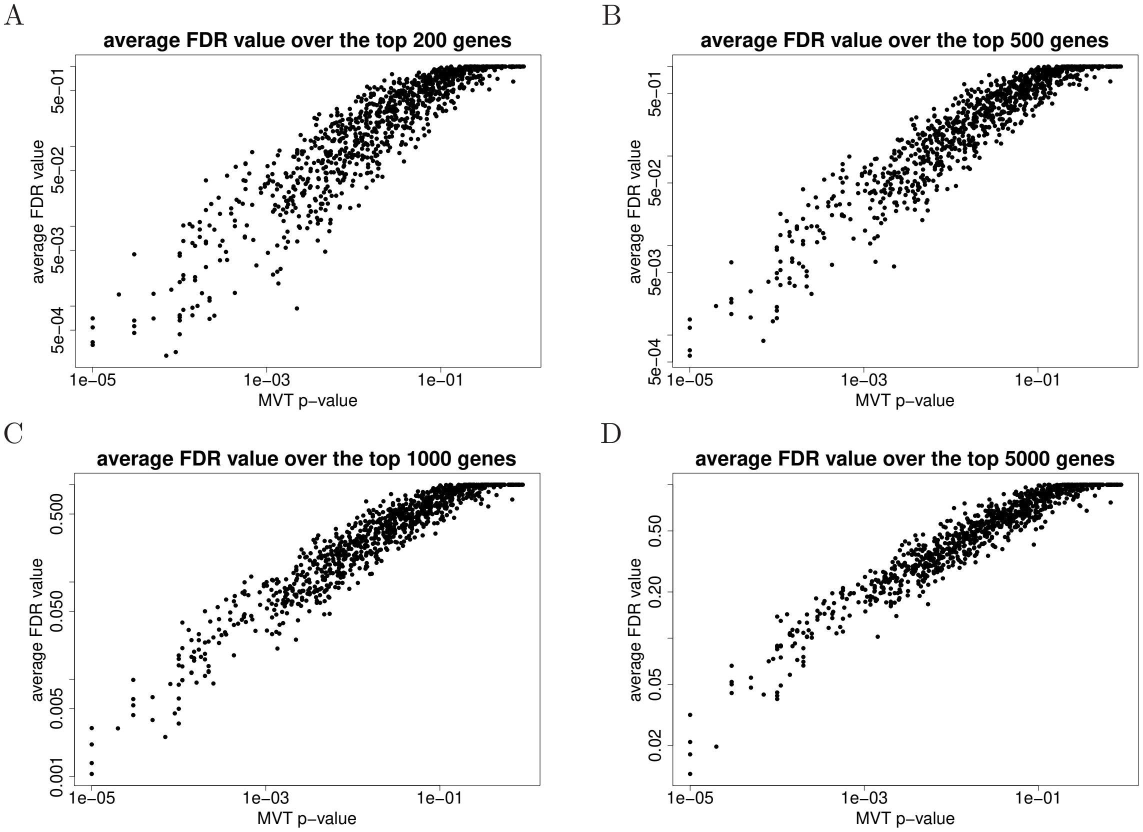

References

[1] S Assassi, WR Swindell, M Wu, FD Tan, D Khanna, DE Furst, DP Tashkin, RR Jahan-Tigh, MD Mayes, JE Gudjonsson, and JT Chang. Dissecting the heterogeneity of skin gene expression patterns in systemic sclerosis. *Arthritis Rheumatol*, 67(11):3016–26, 2015.

[2] T Barrett, SE Wilhite, P Ledoux, C Evangelista, IF Kim, M Tomashevsky, KA Marshall, KH Phillippy, PM Sherman, M Holko, A Yefanov, H Lee, N Zhang, CL Robertson, N Serova, S Davis, and A Soboleva. NCBI GEO: archive for functional genomics data sets – update. *Nucleic Acids Res*, 41(Database issue), 2013.

[3] Yoav Benjamini and Yosef Hochberg. Controlling the false discovery rate: a practical and powerful approach to multiple testing. *Journal of the Royal statistical society: series B (Methodological)*, 57(1):289–300, 1995.

[4] J Bigler, HA Rand, K Kerkof, M Timour, and CB Russell. Cross-study homogeneity of psoriasis gene expression in skin across a large expression range. *PLoS One*, 8(1):e52242, 2013.

[5] J Bigler, HA Rand, K Kerkof, M Timour, and CB Russell. Cross-study homogeneity of psoriasis gene expression in skin across a large expression range. *PLoS One*, 8(1):e52242, 2013.

[6] C Chen, E Mendez, J Houck, W Fan, P Lohavanichbutr, D Doody, B Yueh, ND Futran, M Upton, DG Farwell, SM Schwartz, and LP Zhao. Gene expression profiling identifies genes predictive of oral squamous cell carcinoma. *Cancer Epidemiol Biomarkers Prev*, 17(8):2152–62, 2008.

[7] F Chung and L Lu. The average distances in random graphs with given expected degrees. *Proc Natl Acad Sci U S A*, 99(25):15879–82, 2002.

[8] WS Cleveland, E Grosse, and WM Shyu. Local regression models. In JM Chambers and TJ Hastie, editors, *Statistical Models in S*, chapter 8, pages 309–376. Chapman & Hall/CRC, 1992.

[9] D Cordero, X Sole, M Crous-Bou, R Sanz-Pamplona, L Pare-Brunet, E Guino, D Olivares, A Berenguer, C Santos, R Salazar, S Biondo, and V Moreno. Large differences in global transcriptional regulatory programs of normal and tumor colon cells. *BMC Cancer*, pages 14–708, 2014.

[10] Megan Crow, Nathaniel Lim, Sara Ballouz, Paul Pavlidis, and Jesse Gillis. Predictability of human differential gene expression. *Proceedings of the National Academy of Sciences*, 116(13):6491–6500, 2019.

[11] J Cui, F Li, G Wang, X Fang, JD Puett, and Y Xu. Gene-expression signatures can distinguish gastric cancer grades and stages. *PLos One*, 6(3):e17819, 2011.

[12] JS D'Alessandro, J Duffner, J Pradines, I Capila, K Garofalo, G Kaundinya, BM Greenberg, D Kantor, and TC Ganguly. Equivalent Gene Expression Profiles between Glatopa™and Copaxone©. *PLoS One*, 10(10):e0140299, 2015.

[13] A Franceschini, D Szklarczyk, S Frankild, M Kuhn, M Simonovic, A Roth, J Lin, P Minguez, P Bork, C von Mering, and LJ Jensen. STRING v9.1: protein-protein interaction networks, with increased coverage and integration. *Nucleic Acids Res*, 41(Database issue):D808–15, 2013.

[14] S Goh, I Choi, and N Kim. Comparison of exon-wise expression profiling between normal and cancer tissues of human stomach. *Gene Expression Omnibus*, GSE30727, 2014.

[15] M Hinchcliff, CC Huang, TA Wood, Mahoney Matthew, V Martyanov, S Bhattacharyya, Z Tamaki, J Lee, M Carns, S Podlusky, A Sirajuddin, SJ Shah, RW Chang, R Lafyatis, J Varga, and ML Whitfield. Molecular signatures in skin associated with clinical improvement during mycophenolate treatment in systemic sclerosis. *J Invest Dermatol*, 133(8):1979–89, 2013.

[16] J Hou, J Aerts, B den Hamer, W van Ijcken, M den Bakker, P Riegman, C van der Leest, P van der Spek, JA Foekens, HC Hoogsteden, F Grosveld, and S Philipsen. Gene expression-based classification of non-small cell lung carcinomas and survival prediction. *PLos One*, 5(4):e10312, 2010.

[17] Justin K Huang, Daniel E Carlin, Michael Ku Yu, Wei Zhang, Jason F Kreisberg, Pablo Tamayo, and Trey Ideker. Systematic evaluation of molecular networks for discovery of disease genes. *Cell systems*, 6(4):484–495, 2018.

[18] S Itzkovitz, R Milo, N Kashtan, G Ziv, and U Alon. Subgraphs in random networks. *Phys Rev E Stat Nonlin Soft Matter Phys*, 68(2 Pt 2):026127, 2003.

[19] M Kabbout, MM Garcia, J Fujimoto, DD Liu, D Woods, CW Chow, G Mendoza, AA Momin, BP James, L Solis, C Behrens, JJ Lee, II Wistuba, and H Kadara. Ets2 mediated tumor suppressive function and met oncogene inhibition in human non-small cell lung cancer. *Clin Canc Res*, 19(13):3383–95, 2013.

[20] L Le Cam. An approximation theorem for the Poisson binomial distribution. *Pacif J Math*, 10:1181–97, 1960.

[21] HY Lim, I Sohn, S Deng, J Lee, SH Jung, M Mao, J Xu, K Wang, S Shi, JW Joh, and CK Choi, YLand Park. Prediction of disease-free survival in hepatocellular carcinoma by gene expression profiling. *Ann Surg Oncol*, 20(12):3747–53, 2013.

[22] Consortium MAQC. The MicroArray Quality Control (MAQC) project shows inter- and intraplatform reproducibility of gene expression measurements. *Nat Biotechnol*, 24(9):1151–61, 2006.

[23] A Milano, SA Pendergrass, JL Sargent, LK George, TH McCalmont, MK Connolly, and ML Whitfield. Molecular subsets in the gene expression signatures of scleroderma skin. *PLoS One*, 3(7):e2696, 2008.

[24] R Milo, S Shen-Orr, S Itzkovitz, N Kashtan, D Chklovskii, and U Alon. Network motifs: simple building blocks of complex networks. *Science*, 298(5594):824–7, 2002.

[25] SA Pendergrass, R Lemaire, IP Francis, JM Mahoney, R Lafyatis, and ML Whitfield. Intrinsic gene expression subsets of diffuse cutaneous systemic sclerosis are stable in serial skin biopsies. *J Invest Dermatol*, 132(5):1363–73, 2012.

[26] Rudolf T Pillich, Jing Chen, Vladimir Rynkov, David Welker, and Dexter Pratt. Ndex: a community resource for sharing and publishing of biological networks. In *Protein Bioinformatics*, pages 271–301. Springer, 2017.

[27] J Pradines, V Dancik, A Ruttenberg, and V Farutin. Connectedness profiles in protein networks for the analysis of gene expression data. *Lecture Notes in Bioinformatics*, 4453(RECOMB2007):296–310, 2007.

[28] JR Pradines, V Farutin, S Rowley, and V Dancik. Analyzing protein lists with large networks: edge-count probabilities in random graphs with given expected degrees. *J Comput Biol*, 12(2):113–28, 2005.

[29] Dexter Pratt, Jing Chen, Rudolf Pillich, Vladimir Rynkov, Aaron Gary, Barry Demchak, and Trey Ideker. Ndex 2.0: a clearinghouse for research on cancer pathways. *Cancer research*, 77(21):e58–e61, 2017.

[30] Dexter Pratt, Jing Chen, David Welker, Ricardo Rivas, Rudolf Pillich, Vladimir Rynkov, Keiichiro Ono, Carol Miello, Lyndon Hicks, Sandor Szalma, et al. Ndex, the network data exchange. *Cell systems*, 1(4):302–305, 2015.

[31] R Core Team. *R: A Language and Environment for Statistical Computing*. R Foundation for Statistical Computing, Vienna, Austria, 2017.

[32] Matthew E Ritchie, Belinda Phipson, Di Wu, Yifang Hu, Charity W Law, Wei Shi, and Gordon K Smyth. limma powers differential expression analyses for rna-sequencing and microarray studies. *Nucleic acids research*, 43(7):e47–e47, 2015.

[33] BM Ryan, KA Zanetti, AI Robles, AJ Schetter, J Goodman, RB Hayes, WY Huang, MJ Gunter, M Yeager, L Burdette, SI Berndt, and CC Harris. Germline variation in ncf4, an innate immunity gene, is associated with an increased risk of colorectal cancer. *Int J Cancer*, 134(6):1399–407, 2014.

[34] Q Shao, H Yao, J He, and Y Jin. Expression data from gastric cancer and paired normal tissues. *Gene Expression Omnibus*, GSE79973, 2016.

[35] M Sheffer, MD Bacolod, O Zuk, SF Giardina, H Pincas, F Barany, PB Paty, WL Gerald, DA Notterman, and E Domany. Association of survival and disease progression with chromosomal instability: a genomic exploration of colorectal cancer. *Proc Natl Acad Sci U S A*, 106(17):7131–6, 2009.

[36] O Sobolev, E Binda, S O'Farrell, A Lorenc, J Pradines, H Huang, J Duffner, R Schulz, J Cason, M Malim, A Cope, M Peakman, I Capila, G Kaundinya, and A Hayday. Adjuvanted influenza-H1N1 vaccination reveals lymphoid signatures of age-dependent early responses and of clinical adverse events. *Nature Immunology*, 17(2):204–13, 2016.

[37] M Suarez-Farinas, K Li, J Fuentes-Duculan, K Hayden, C Brodmerkel, and JG Krueger. Expanding the psoriasis disease profile: interrogation of the skin and serum o patients with moderate-to-severe psoriasis. *J Invest Dermatol*, 132(11):2552–64, 2012.

[38] WR Swindell, A Johnston, S Carbajal, G Han, C Wohn, J Lu, X Xing, RP Nair, JJ Voorhees, JT Elder, XJ Wang, S Sano, EP Prens, J DiGiovanni, MR Pittelkow, NL Ward, and JE Gudjonsson. Genome-wide expression profiling of five mouse models identifies similarities and differences with human psoriasis. *PLoS One*, 6(4):e18266, 2011.

[39] D Szklarczyk, A Franceschini, S Wyder, K Forslund, D Heller, J Huerta-Cepas, M Simonovic, A Roth, A Santos, KP Tsafou, M Kuhn, P Bork, LJ Jensen, and C von Mering. STRING v10: protein-protein interaction networks, integrated over the tree of life. *Nucleic Acids Res*, 43(Database issue):D447–52, 2015.

[40] JS Tamaresis, JC Irwin, GA Goldfien, JT Rabban, RO Burney, C Nezhat, LV DePaolo, and LC Giudice. Molecular classification of endometriosis and disease stage using high-dimensional genomic data. *Endocrinology*, 155(12):4986–99, 2014.

[41] C von Mering, LJ Jensen, B Snel, SD Hooper, M Krupp, M Foglierini, N Jouffre, MA Huynen, and P Bork. STRING: known and predicted protein-protein associations, integrated and transferred across organisms. *Nucleic Acids Res*, 33(Database issue):D433–7, 2005.

[42] Q Wang, YG Wen, DP Li, J Xia, CZ Zhou, DW Yan, HM Tang, and ZH Peng. Upregulated inhba expression is associated with poor survival in gastric cancer. *Med Oncol*, 29(1):77–83, 2012.

[43] Y Yang, W Zhang, H Gao, and Q Zhang. Gene expression and alternative splicing in human gastric cancer. *Gene Expression Omnibus*, GSE13195, 2009.

[44] X Zhang, Z Ni, Z Duan, and Z et al Xin. Overexpression of e2f mrnas associated with gastric cancer progression identified by the transcription factor and mirna co-regulatory network analysis. *PLoS One*, 10(2):e0116979, 2015.
